# Cascade Metathesis Reactions for the Synthesis of Taxane and Isotaxane Derivatives

**DOI:** 10.1002/chem.201600592

**Published:** 2016-04-08

**Authors:** Cong Ma, Aurélien Letort, Rémi Aouzal, Antonia Wilkes, Gourhari Maiti, Louis J. Farrugia, Louis Ricard, Joëlle Prunet

**Affiliations:** ^1^ WestCHEMSchool of ChemistryUniversity of Glasgow, Joseph Black BuildingUniversity AvenueGlasgowG12 8QQUK; ^2^ Laboratoire de Synthèse OrganiqueCNRS UMR 7652Ecole Polytechnique, DCSO91128PalaiseauFrance; ^3^ Laboratoire de Chimie MoléculaireCNRS UMR 9168Ecole Polytechnique, LCM91128PalaiseauFrance

**Keywords:** cyclooctenes, enyne, metathesis, synthesis design, taxanes

## Abstract

Tricyclic isotaxane and taxane derivatives have been synthesized by a very efficient cascade ring‐closing dienyne metathesis (RCDEYM) reaction, which formed the A and B rings in one operation. When the alkyne is present at C13 (with no neighboring *gem*‐dimethyl group), the RCEDYM reaction leads to 14,15‐isotaxanes **16 a**,**b** and **18 b** with the *gem*‐dimethyl group on the A ring. If the alkyne is at the C11 position (and thus flanked by a *gem*‐dimethyl group), RCEDYM reaction only proceeds in the presence of a trisubstituted olefin at C13, which disfavors the competing diene ring‐closing metathesis reaction, to give the tricyclic core of Taxol **44**.

## Introduction

**Scheme 1:**
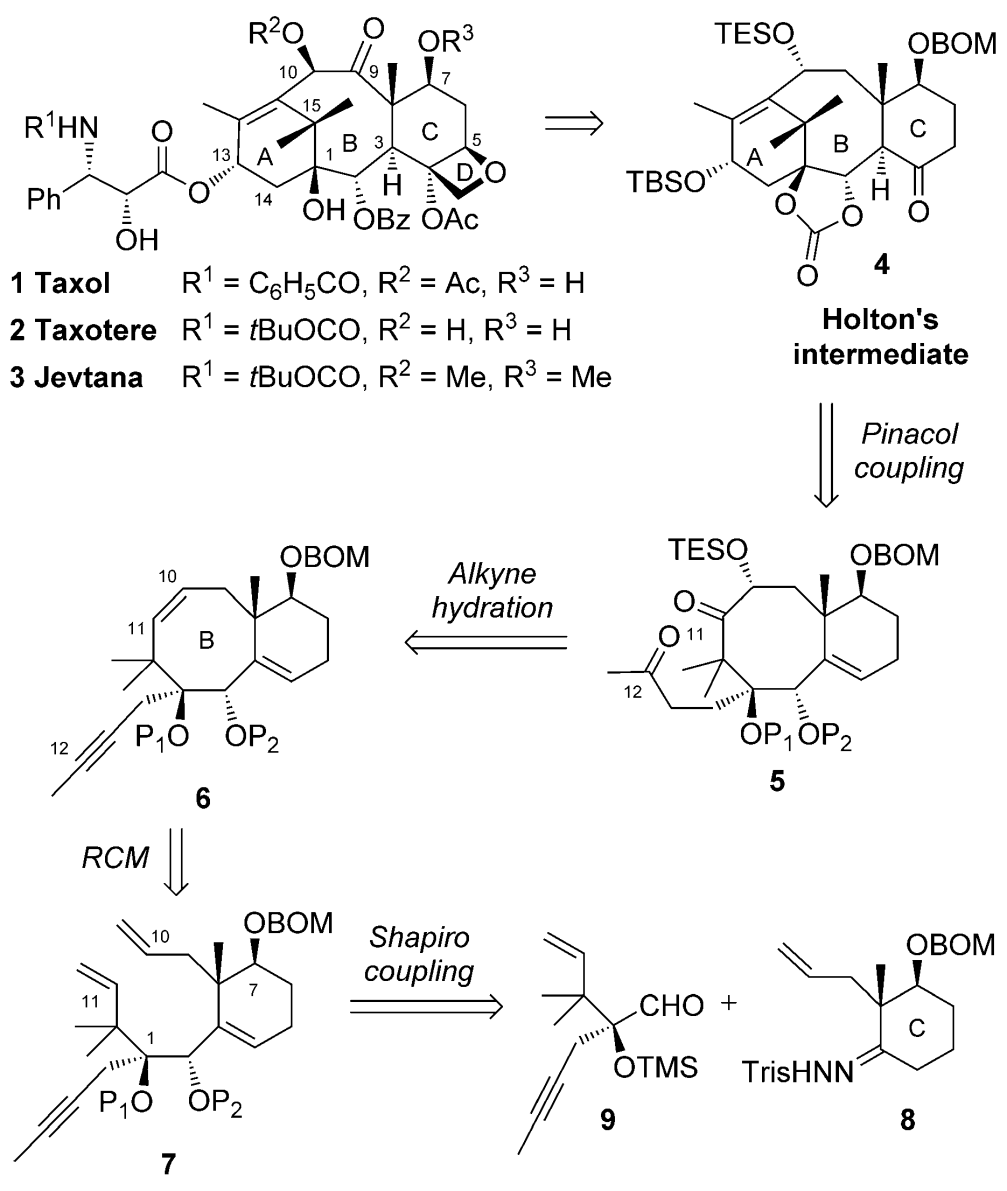
Taxol and derivatives; retrosynthesis of the ABC tricycle of Taxol featuring a ring‐closing metathesis (RCM).

Taxol^®^ (paclitaxel), together with its derivatives Taxotere^®^ (docetaxel) and Jevtana^®^ (cabazitaxel) are the largest selling anticancer drugs of all time, with sales of over three billion USD per year for Taxotere alone in 2010.^1^ Originally indicated for the treatment of ovarian and breast cancers, they are now widely prescribed to treat a broad range of malignancies.^2^ The structures of these three compounds only differ in terms of the functionalization of the amine on the side chain and the hydroxyl groups at C10 and C7 (Scheme 1). Taxol is currently being manufactured through plant‐cell fermentation by Phyton Biotech, LLC, a DFB Pharmaceuticals Company for Bristol–Myers Squibb, whilst Taxotere and Jevtana are produced by semisynthesis from 10‐deacetylbaccatin III by Sanofi, which still requires an expensive extraction process of natural resources. There have been six total syntheses of Taxol by the groups of Holton,^3^ Nicolaou,^4^ Danishefsky,^5^ Wender,^6^ Mukaiyama^7^ and Kuwajima,^8^ as well as three formal syntheses by the groups of Takahashi,^9^ Nakada^10^ as well as Sato and Chida,^11^ but they all comprise at least 37 steps.^12^ An efficient synthesis of active taxoid analogues has yet to be achieved, because of the sterically hindered, complex and highly functionalized structure of these compounds.1

A rapid synthesis of the tricyclic core of Taxol where all of the functional groups required for activity are present or in a latent form would facilitate access to a diverse array of novel taxoids with potential anticancer activity. We report here a synthetic strategy featuring a cascade ring‐closing dienyne metathesis (RCDEYM) reaction that allows access to the ABC tricyclic ring system of taxanes as well as taxane analogues that possess a novel skeleton and cannot be prepared by semi‐synthesis.^13^

## Results and Discussion

Our initial retrosynthesis is outlined in Scheme 1. We aimed for a formal synthesis of Taxol, so we chose the intermediate **4** described by Holton during his synthesis of this natural product as our primary target.^3^ The A ring would be closed by a pinacol coupling between the ketones at C11 and C12 in compound **5**, as previously described by Mukaiyama on a similar substrate.^7^ The ketone at C12 would be installed by hydration of alkyne **6**. The eight‐membered B ring would be formed by a ring‐closing metathesis (RCM) reaction^14^ between the alkenes at C10 and C11 in compound **7**. This key step was successful in our synthesis of model BC bicyclic systems of Taxol (with no hydroxyl group at C7 and a butyl side chain at C1).^15^ Finally, the metathesis precursor **7** would be assembled by a Shapiro reaction between hydrazone **8** and aldehyde **9**. This coupling reaction has proved to be very diastereoselective on similar substrates during our previous approaches to taxoids.^16^

**Scheme 2:**
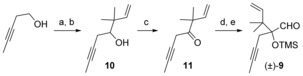
Synthesis of aldehyde (±)‐**9**. a) DMP, CH_2_Cl_2_; b) Zn, NH_4_Cl, prenyl bromide, 99 % (over 2 steps); c) a) DMP, CH_2_Cl_2_, 95 %; d) TMSCN, DABCO, CH_2_Cl_2_; e) DIBAL‐H, CH_2_Cl_2_; SiO_2_ (58 % over 2 steps). DMP=Dess–Martin periodinane, TMS=trimethylsilyl, DABCO=1,4‐diazabicyclo[2.2.2]octane, DIBAL‐H=diisobutylaluminium hydride.

Our synthesis commenced with the preparation of aldehyde **9** (Scheme 2). Commercially available 3‐pentyn‐1‐ol was oxidized with the Dess–Martin periodinane^17^ (DMP) and the resulting aldehyde was subjected to a Barbier reaction with prenyl bromide under the Luche conditions^18^ to furnish alcohol **10** in excellent yield. Oxidation of alcohol **10** gave the corresponding ketone **11**, which was submitted to trimethylsilyl cyanide in the presence of a the tertiary amine 1,4‐diazabicyclo[2.2.2]octane (DABCO) as a catalyst. The resulting cyanohydrin was reduced to the intermediate imine, which was hydrolyzed to give the racemic aldehyde (±)‐**9** by exposure to silica gel. Optically active aldehyde **9** was also prepared in 99 % *ee* in a similar fashion^19^ using a chiral amine base for the cyanation reaction,^20^ but we chose to pursue the synthesis of the metathesis precursors with the racemic aldehyde to widen the array of taxoids generated, and to study the influence of the stereochemistry of the precursor on the RCM reaction outcome.2

**Scheme 3:**
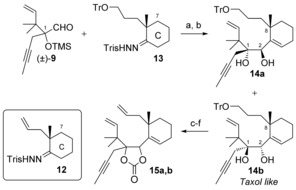
Synthesis of metathesis precursors **15 a**,**b**. a) *t*BuLi, CeCl_3_, THF, −78 °C; b) 1 N aq. HCl, **14 a** 45 % (over 2 steps), **14 b** 40 % (over 2 steps); c) Amberlyst H‐15, MeOH; d) *o*‐NO_2_C_6_H_4_SeCN, PBu_3_, THF; e) Im_2_CO, toluene, 110 °C; f) (NH_4_)_6_Mo_7_O_24_⋅4 H_2_O, H_2_O_2_, H_2_O, **15 a**, 75 % (over 4 steps), **15 b** 65 % (over 4 steps). THF=tetrahydrofuran, Im=imidazolyl.

In order to test the key metathesis reaction, we decided to use a 7‐deoxy C ring as a coupling partner in the Shapiro reaction. It is worth noting that removal of the functional group at C7 in Taxol did not result in a significant loss of bioactivity.^21^ When hydrazone **12** (Scheme 3), prepared in 76 % yield from the corresponding known ketone,^22^ was submitted to *t*BuLi for the Shapiro coupling, only degradation was observed.^23^ We surmised that this was due to the deprotonation at the allylic position, and thus the alkene was masked as a protected primary alcohol. Enantiopure hydrazone **13** 15b was treated with aldehyde (±)‐**9** using conditions we had developed previously.15b To our surprise, the reaction only proceeded in 20 % yield. Several additives were screened. Addition of MgBr_2_ and ZnCl_2_ did not lead to any of the desired product, but we observed a dramatic increase in yield when dry CeCl_3_ was stirred for 30 min with the vinyllithium reagent derived from hydrazone **13** before addition of aldehyde (±)‐**9**, and diols **14 a**,**b** were obtained in 85 % combined yield after hydrolysis of the TMS ether. The reason for this difference in reactivity between the model aldehyde (butyl side chain at C1) and (±)‐**9**(2‐butynyl side chain at C1) is unclear.15b As had been observed previously for the model aldehydes, this reaction was highly diastereoselective, giving the *trans* diol compounds^24^ **14 a** and **14 b** after hydrolysis of the trimethylsilyl ether. The stereochemistry of **14 a** and **14 b** was assigned by comparing their proton NMR spectra with those of the corresponding model Shapiro adducts possessing a butyl side chain at C1.^25^ Diols **14 a** and **14 b** were then submitted separately to trityl ether hydrolysis, elimination of the resulting primary alcohol using the Grieco protocol^26^ and protection of the C1‐C2 diol as the cyclic carbonate ester to furnish the metathesis precursors **15 a** and **15 b** in 75 % and 65 % overall yields for the four steps, respectively. No intermediate purification was required for these transformations.3

**Scheme 4:**
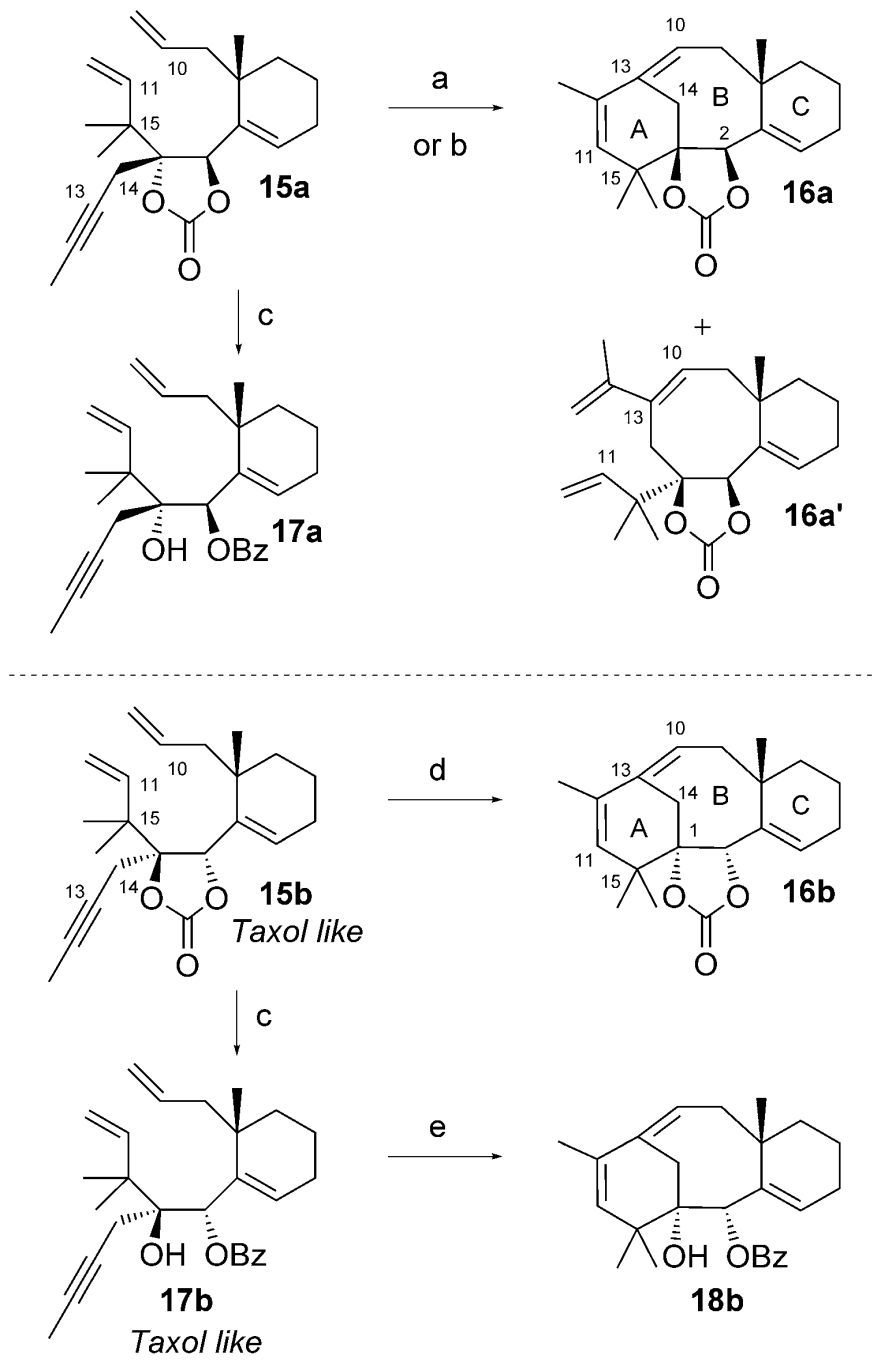
Attempts at metathesis and synthesis of isotaxanes. a) 5 mol % Grubbs 2, toluene, 110 °C, 48 h, 1:1 **16 a/16 a’** 30 %; b) 10 mol % Grubbs 2, toluene, 110 °C, 24 h, **16 a** 62 %; c) PhLi, THF, −78 °C, **17 a** 54 %, **17 b** 70 %; d) 5 mol % Grubbs 2, toluene, 80 °C, 2 h, 91 %; e) 5 mol % Grubbs 2, toluene, 110 °C, 1 h, 57 %. For the structure of Grubbs 2, see Table 1.

We first tried out the key RCM reaction on carbonate **15 a**, which possesses the opposite configuration at C1 and C2 compared to Taxol. Treatment of this compound with 10 mol % of the second‐generation Grubbs precatalyst in toluene at reflux for 24 h did not lead to the desired cyclooctene, but gave tricyclic derivative **16 a** instead (Scheme 4). This product resulted from an enyne metathesis reaction between the alkene at C10 and the alkyne at C13, furnishing the intermediate bicycle **16 a′**, which further cyclized by a diene metathesis to give **16 a** in good yield. This intermediate **16 a′** could be isolated as a 1:1 mixture with **16 a** if only 5 mol % of the precatalyst was used for the reaction. The first enyne metathesis reaction was not unexpected;^27^ what was more surprising to us was the ease of formation of the strained tricyclic system in compound **16 a**. This 14,15‐isotaxane has an unprecedented skeleton, which is very similar to that of taxane derivatives, except that the C14 and C15 carbons have swapped positions, which places the *gem*‐dimethyl group in the A‐ring alone. In addition, the C4 stereogenic center possesses the undesired configuration for Taxol.

In an effort to assess the influence of the nature of the diol protecting group on the outcome of the metathesis reaction, which was shown to be crucial for model compounds,15b the benzoate **17 a** was prepared by addition of phenyllithium to the carbonate **15 a** (Scheme 4). Unfortunately, benzoate **17 a** did not undergo metathesis when treated with the Grubbs 2 precatalyst, but slowly decomposed.

**Figure 1:**
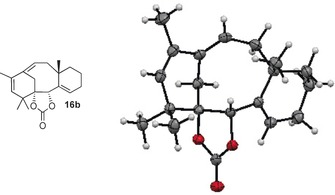
ORTEP (50 % probability) representation of compound **16 b**.

A similar cascade dienyne metathesis reaction was observed with stereoisomer **15 b**, but the reaction proceeded under milder conditions and gave tricycle **16 b** in 91 % yield (Scheme 4). This time, RCM of benzoate **17 b**, obtained by phenyllithium addition to **15 b**, furnished isotaxane **18 b** in 57 % yield, underscoring the influence of the configuration at C1 and C2 on the outcome of the metathesis reactions.15b These isotaxanes possess the undesired configuration at C1. Isotaxane **16 b** was crystalline, and its X‐ray crystallographic analysis^28^ (Figure 1) established its tricyclic structure and confirmed the configuration of the carbonate‐bearing stereocenters at C1 and C1

**Figure 2:**
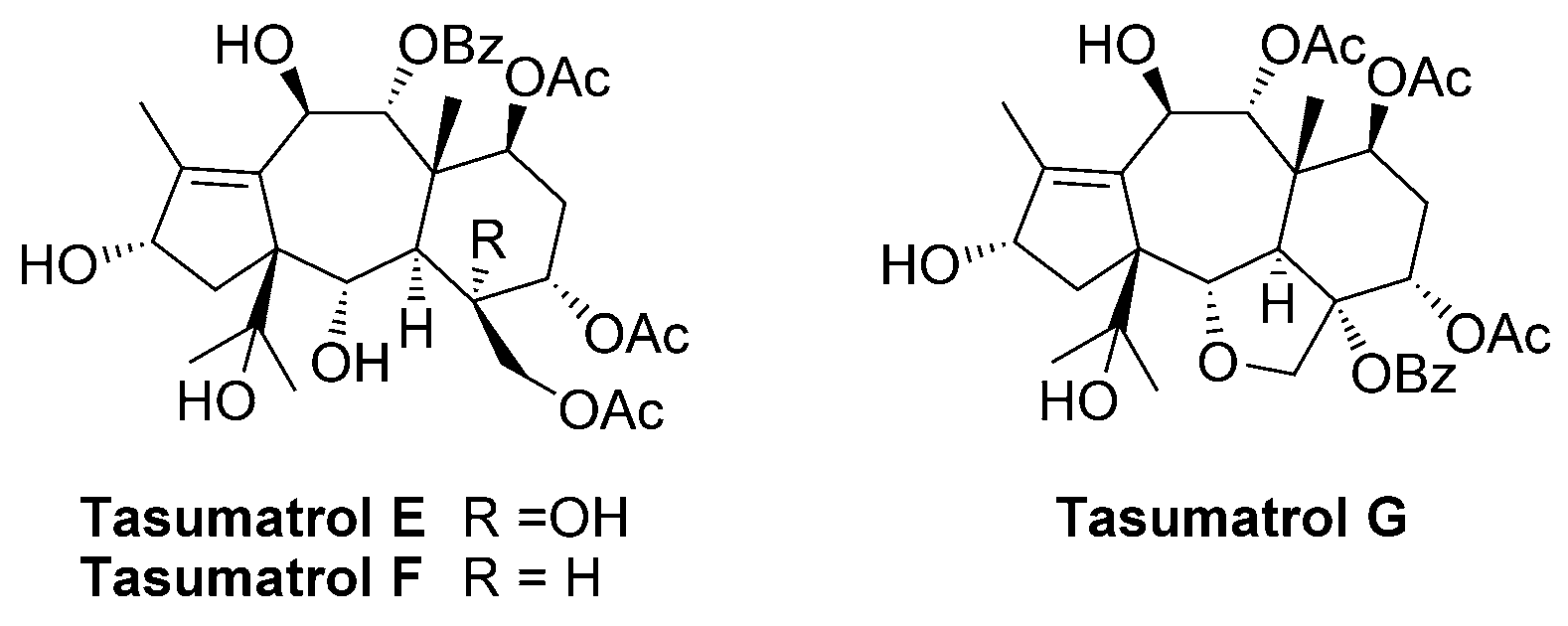
Structures of tasumatrols E, F and G.

The isotaxanes **16 a**, **16 b** and **18 b** represent a novel class of Taxol analogues, and could be transformed into potentially active compounds. Indeed, taxanes such as tasumatrols E, F and G (Figure 2), isolated from *Taxus sumatrana*, do not possess the classical ABC 6,8,6‐tricyclic system of Taxol; however, they exhibit more potent activity than Taxol in vitro against four human cancer cell lines.^29^, 2

**Scheme 5:**
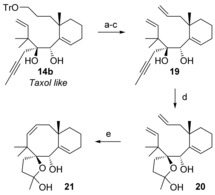
Alkyne hydration followed by RCM. a) Amberlyst H‐15, MeOH, 98 %; b) PPh_3_, imidazole, I_2_, 82 %; c) NaH, DMF, 84 %; d) Ph_3_PAuNTf_2_, THF, H_2_O, 80 %; e) 5 mol % Grubbs 2, CH_2_Cl_2_, reflux, 1.5 h, 98 %. DMF=dimethylformamide, Tf=trifluroromethanesulfonyl.

An easy way to circumvent the unwanted dienyne metathesis cascade is to perform the alkyne hydration before the RCM step, and this has been achieved in excellent yield (Scheme 5). Diol **19** was prepared in three steps from the Shapiro adduct **14 b** in 68 % overall yield. Treatment of alkyne **19** with the Gagosz catalyst^30^ in the presence of water did not give the corresponding ketone but hemiketal **20**.^31^ Fortunately, compound **20** underwent ring‐closing metathesis in 98 % yield to form the BC ring system of Taxol **21**. Work is in progress for the completion of the synthesis of the tricyclic core of Taxol according to the retrosynthesis shown in Scheme 1.5

**Scheme 6:**
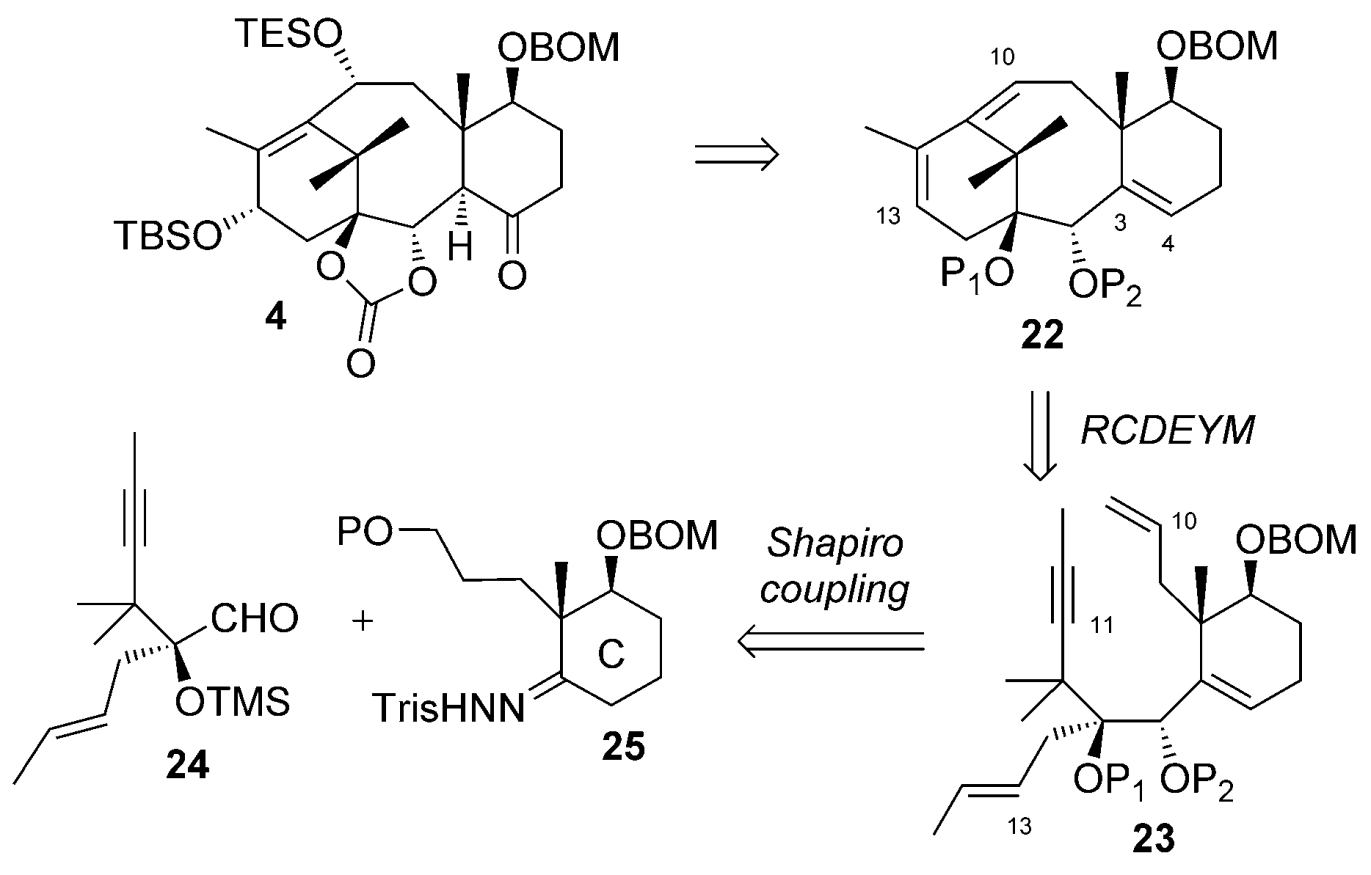
Novel retrosynthesis of taxol featuring a cascade ring‐closing dienyne metathesis (RCDEYM).

On the other hand, we also wanted to take advantage of this very efficient metathesis cascade to synthesize the ABC tricycle of Taxol, and our revised retrosynthesis is shown in Scheme 6. The 2‐ene‐1,4‐diol unit of compound **4** would be installed by a Ti^III^ radical‐mediated opening of the corresponding 1,3‐diepoxide, which can be generated from the 1,3‐diene moiety at C10‐C13 of compound **22**.12b A hydroboration/oxidation sequence of the C3‐C4 olefin would lead to the ketone at C4.^5^ Tricycle **22** would be formed by a metathesis cascade reaction from dienyne **23**, where the alkyne at C11 and the alkene at C13 have swapped positions compared to those in compound **15 b**. In order to direct the metathesis cascade reaction so it starts with the olefin at C10 and not with the one at C13, we elected to have a disubstituted olefin at C13, which would react more slowly with the metathesis precatalysts. It is important to note that this extra methyl group will not be present in the metathesis product **22**, but will be part of the propene released after the diene metathesis reaction. Disconnection of dienyne **23** through the C2−C3 bond reveals the two precursors aldehyde **24** and hydrazone 6.

**Scheme 7:**
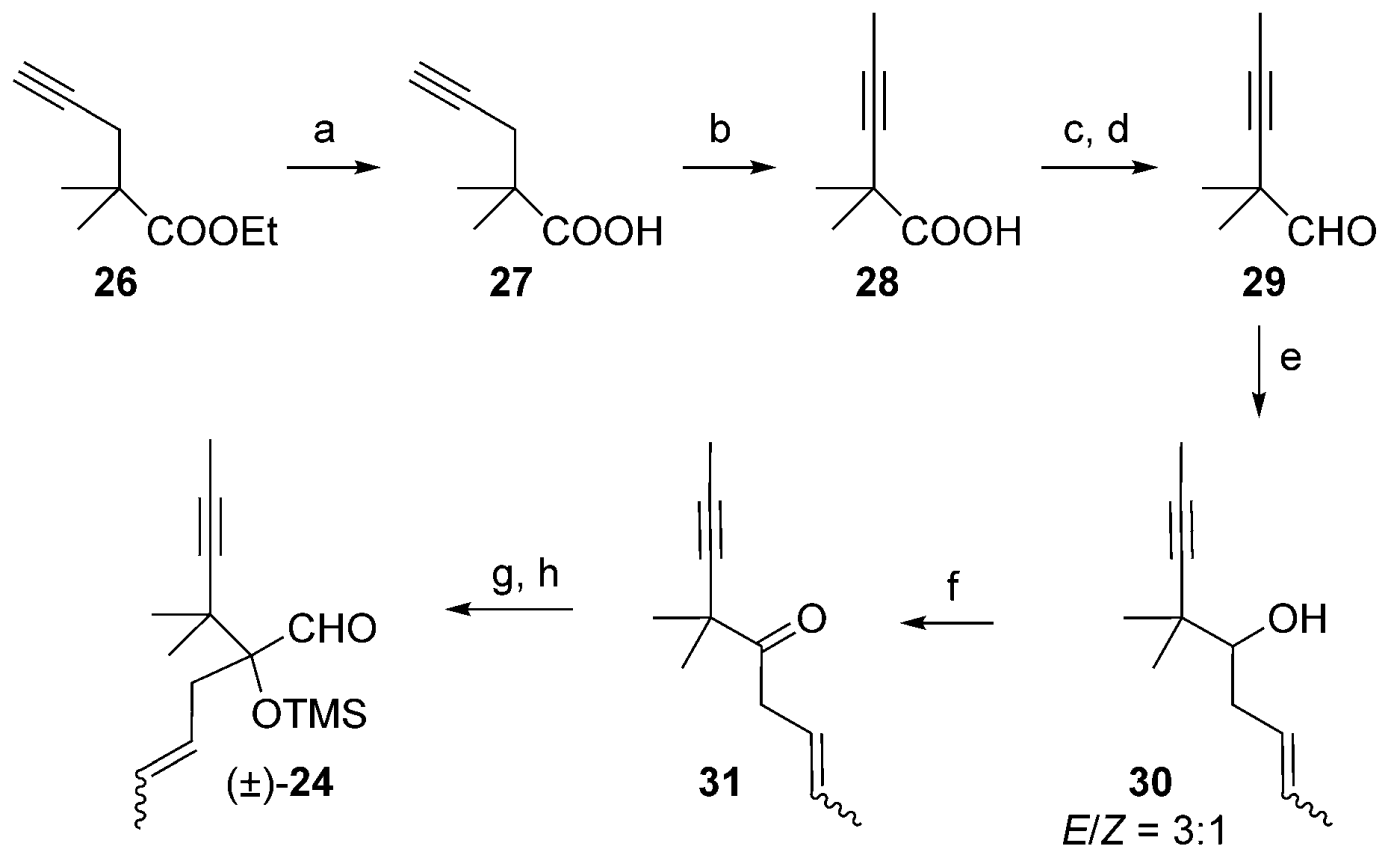
Synthesis of aldehyde (±)‐**24**. a) KOH, H_2_O, MeOH, 93 %; b) *t*BuOK, DMSO, 75 °C, 94 %; c) LiAlH_4_, THF; d) (COCl)_2_, DMSO, Et_3_N, CH_2_Cl_2_, 76 % (over 2 steps); e) 2,3‐dimethyl‐4‐penten‐2‐ol, Sn(OTf)_2_, CH_2_Cl_2_, 76 %; f) IBX, DMSO, THF, 93 %; g) TMSCN, ZnI_2_, CH_2_Cl_2_, reflux; g) DIBAL‐H, hexane; SiO_2_ (62 % over 2 steps). DMSO=dimethyl sulfoxide, IBX=2‐iodoxybenzoic acid.

The synthesis of aldehyde **24** in its racemic form was not as straightforward as the synthesis of the corresponding aldehyde **9**. It started with ester **26**,^32^ obtained by propargylation of ethyl isobutyrate (Scheme 7). Attempts to isomerize the terminal alkyne of **26** into the internal one with potassium *tert*‐butoxide only resulted in degradation products. Fortunately, this isomerization reaction was successful on the corresponding acid **27**, and acid **28** was obtained in 94 % yield. Addition of crotylmagnesium chloride to the corresponding Weinreb amide (compound **41**, see Scheme 9 for structure) furnished a complex mixture of products, so we next turned to the crotylation of aldehyde **29**. Treatment of this aldehyde with crotyl magnesium chloride in the presence of aluminum trichloride^33^ led to a 1:1.5 mixture of α and γ crotylation products. Fortunately, allyl transfer from 2,3‐dimethyl‐4‐penten‐2‐ol catalyzed by tin(II) triflate^34^ gave the desired alcohol **30** (as an inconsequential 3:1 mixture of *E*/*Z* isomers) in 76 % yield after 2 days. Oxidation of **30** with 2‐iodoxybenzoic acid (IBX) followed by homologation of the resulting ketone **31** furnished aldehyde (±)‐**24** 35 in good overall yield.7

**Scheme 8:**
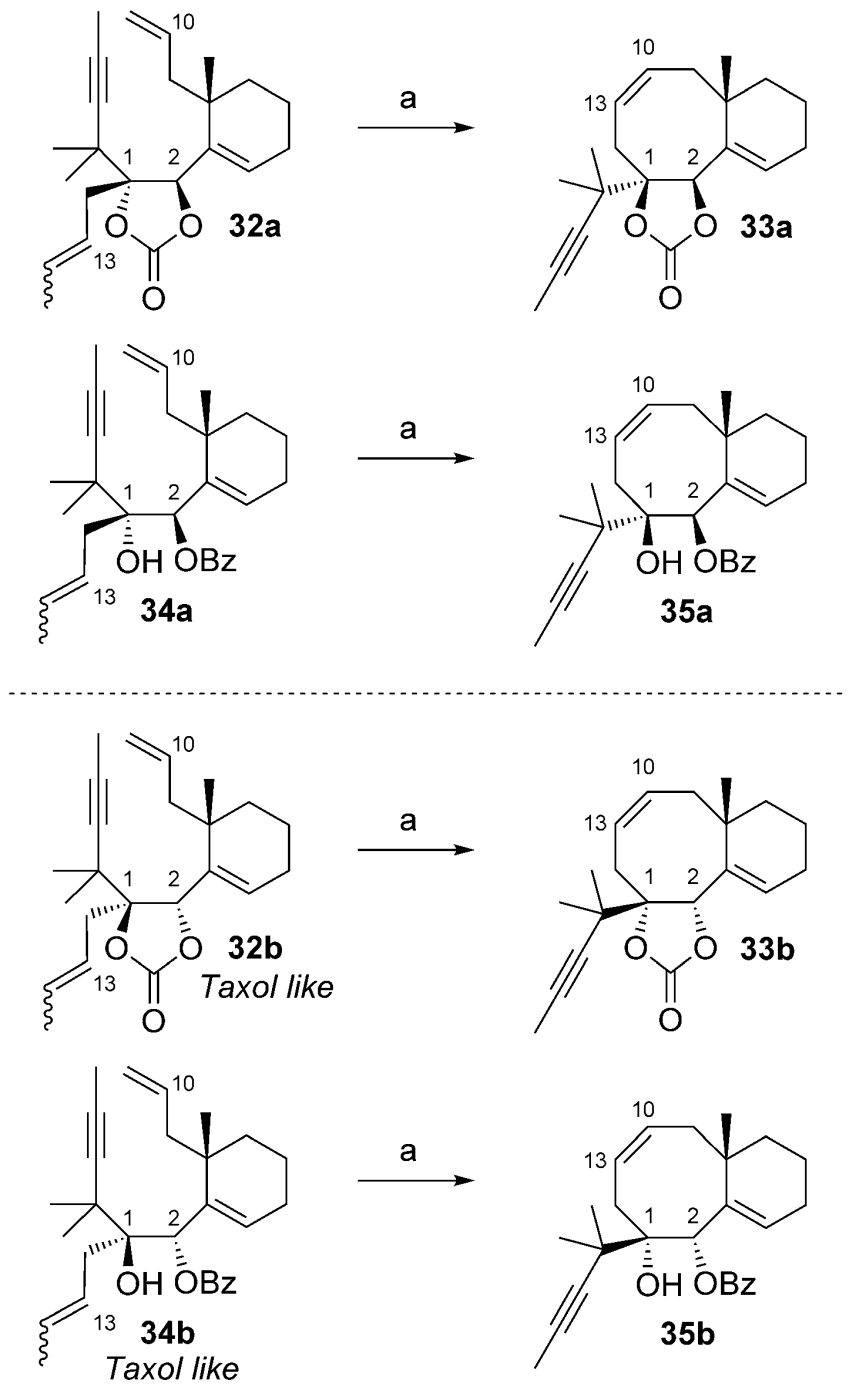
Attempts at metathesis cascade. a) 10 mol % Grubbs 2, toluene, 110 °C, 12 h, **33 a** 63 %, **35 a** 78 %, **33 b** 68 %, **35 b** 86 %.

The dienynes **32 a**,**b** and **34 a**,**b** were prepared using a similar reaction sequence to the one used for compounds **15 a**,**b** and **17 a**,**b**, as described in the preliminary account of our work.^13^ Metathesis reactions of carbonates **32 a**,**b** and benzoates **34 a**,**b** with Grubbs 2 precatalyst did not produce tricyclic compounds, but led to the bicycles **33 a**,**b** and **35 a**,**b**, respectively, resulting from a simple diene RCM between the olefins at C10 and C13 (Scheme 8).^13^, 8

**Figure 3:**
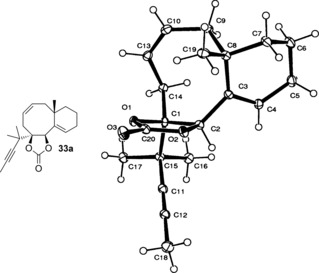
ORTEP (50 % probability) representation of compound **33 a**.

Compound **33 a** was crystalline, and its X‐ray crystallographic analysis^36^ (Figure 3) confirmed the configuration at C1 and C2 of the metathesis precursors **32 a** and **34 a**.3

**Scheme 9:**
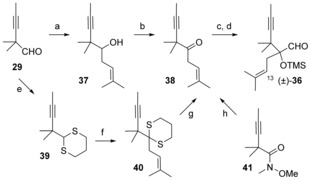
Synthesis of aldehyde (±)‐**36**. a) 2,3,3‐Trimethyl‐4‐penten‐2‐ol, Sn(OTf)_2_, CH_2_Cl_2_, 15–61 %; b) IBX, DMSO, THF, 85 %; c) TMSCN, ZnI_2_, CH_2_Cl_2_; d) DIBAL‐H, hexane; SiO_2_ (83 % over 2 steps); e) 1,2‐ethanedithiol; BF_3_⋅OEt_2_, CH_2_Cl_2_, 75 %; f) BuLi, prenyl bromide, THF, −78 °C, 99 %; g) MeI, CaCO_3_, MeCN, H_2_O, 83 %; h) Mg, THF, prenyl chloride, 95 %.

We had assumed that in the case of compounds **15** and **17** (Scheme 4), the initial enyne methathesis (between C10 and C13) would be favored compared to the alternative diene metathesis (between C10 and C11) because it would lead to a more stable tricyclic product after subsequent diene metathesis, but it seems that in all cases the first RCM takes place with the less hindered unsaturated functional group having no neighboring *gem*‐dimethyl group. Since this *gem*‐dimethyl group is part of the Taxol skeleton, it is not possible to relieve the steric hindrance at the propargylic position in compounds **32** and **34**, but another option is to increase the steric hindrance of the alkene at C13, so the undesired diene RCM is disfavored. We thus embarked on the synthesis of metathesis precursors bearing a trisubstituted olefin at C13. The synthesis of the aldehyde (±)‐**36** required for their preparation is shown in Scheme 9. Prenyl transfer to aldehyde **29** from 2,3,3‐trimethyl‐4‐penten‐2‐ol was unreliable, with yields of **37** ranging from 15 to 61 %. Ketone **38** was then obtained by IBX oxidation. An umpolung synthesis of **38** was also achieved. Prenylation of dithiane **39**, prepared from aldehyde **29**, furnished **40** in excellent yield. Hydrolysis of the dithiane moiety gave ketone **38**. However, this route was not very convenient on large scale, so as a last resort prenylation of the Weinreb amide **41** derived from acid **28** (Scheme 7) was also attempted. To our surprise, this reaction was very clean and afforded ketone **38** in 95 % yield. In this fashion, aldehyde (±)‐**36** 37 was obtained after homologation of **38** in 7 steps and 66 % overall yield from ethyl isobutyrate.9

**Scheme 10:**
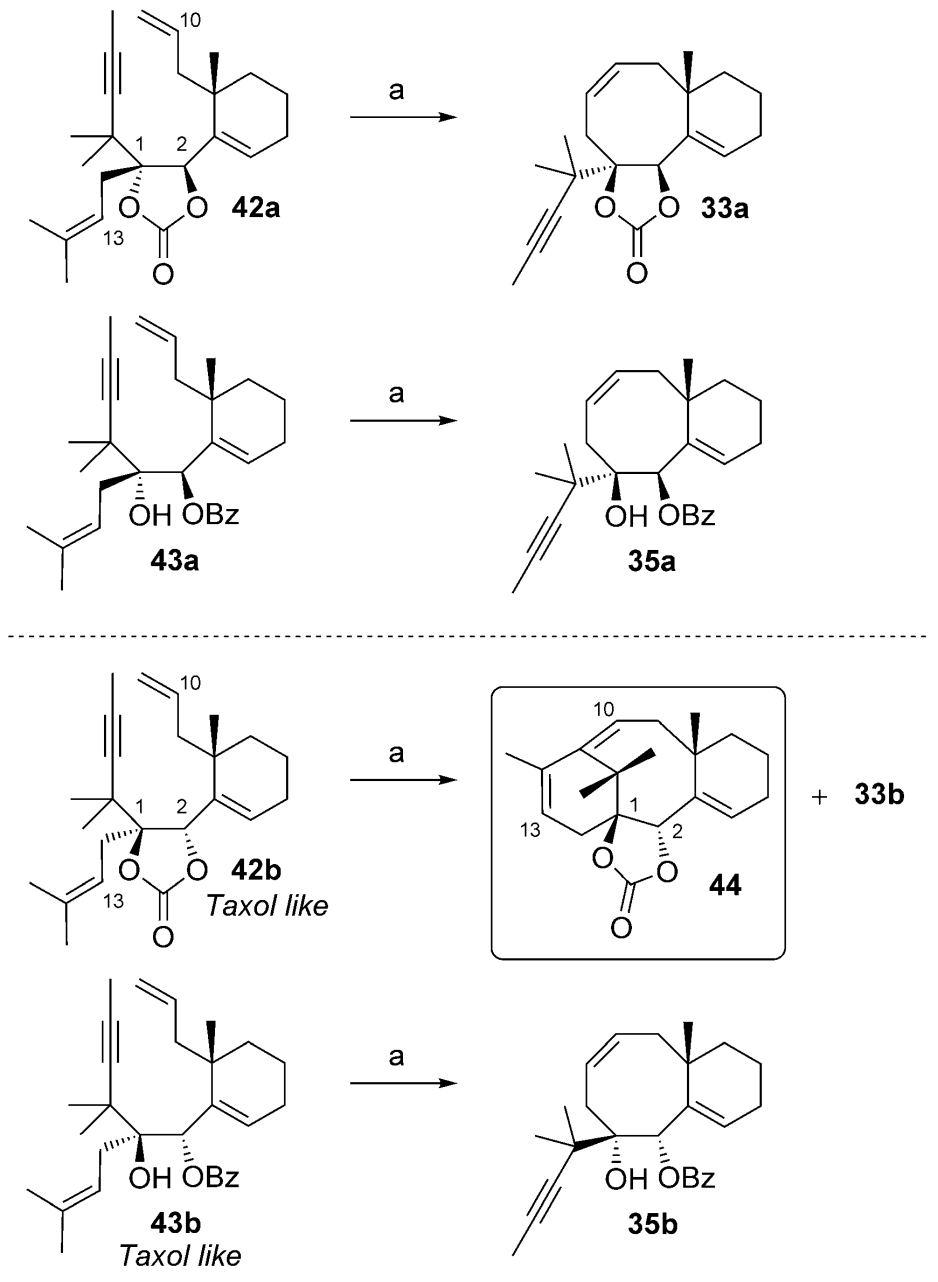
Attempts at metathesis cascade and synthesis of the ABC tricycle of taxol. a) 10 mol % Grubbs 2, toluene, 110 °C, 24 h, **33 a** 79 %, **35 a** 80 %, **44** 45 % and **33 b** 45 %, **35 b** 90 %.

Compounds **42 a**,**b** and **43 a**,**b** bearing a trisubstituted olefin at C13 were synthesized in the same way as compounds **32 a**,**b** and **34 a**,**b** as previously described, then subjected to the Grubbs 2 precatalyst in toluene at reflux (Scheme 10).^13^ We were disappointed to find out that carbonate **42 a** and benzoate **43 a**, possessing the undesired configurations at C1 and C2, furnished the bicyclic compounds **33 a** and **35 a** that we had already observed for the metathesis reactions of **32 a** and **34 a** (Scheme 8). Taxol‐like benzoate **43 b** also underwent diene RCM to produce **35 b**. However, Taxol‐like carbonate **42 b** furnished compound **44** after RCDEYM, which corresponds to the tricyclic core of Taxol, along with the undesired bicyclic compound **33 b**.^13^, 10

**Scheme 11:**
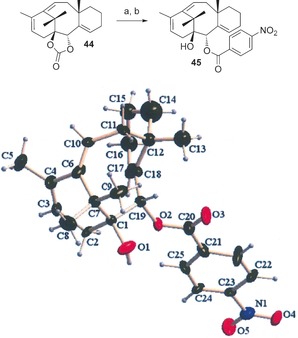
Synthesis and ORTEP (50 % probability) representation of *p*‐nitrobenzoate **45**. a) 2 N aq. NaOH, 1,4‐dioxane, 80 %; b) *p*‐nitrobenzoyl chloride, DMAP, Et_3_N, CH_2_Cl_2_, 80 %.

In order to confirm the structure of the highly strained tricyclic product **44**, we converted it to the crystalline *p*‐nitrobenzoate derivative **45** by hydrolysis of the carbonate and acylation of the resulting secondary alcohol (Scheme 11). X‐ray crystallographic analysis of **45** 38 not only confirmed the tricyclic structure, but also established without ambiguity the configurations at C1 and C2 for the Taxol‐like series of compounds.11

We then set to optimize the yield of the desired tricyclic compound **44**. The **44**/**33 b** ratio was the same under different concentrations ranging from 3×10^−3^ to 15×10^−3^ m,^13^ so all metathesis reactions were performed at 5×10^−3^ m. Toluene at reflux proved to be a better choice than 1,2‐dichloroethane at reflux (80 °C) or xylene at reflux (140 °C).^13^ Various precatalysts were then screened (Table 1). No reaction was observed with the less reactive Grubbs 1 precatalyst, so we tested second‐generation ruthenium complexes. The Hoveyda–Grubbs precatalyst HG2 gave an improved yield of the desired compound **44** compared to the Grubbs 2 precatalyst (69 vs. 45 %), and so did the Grela complex, which possesses a nitro substituent on the benzylidene ligand. Pleasingly, the HG2 derivative Zhan‐1B, which possesses a *N*,*N*‐dimethylsulfonamido group gave the best yield (70 %) of compound **44**. It seems that RCDYEM is favored with precatalysts possessing high initiation rates.1

**Table 1:** Optimization of the formation of **44**.^[a]^

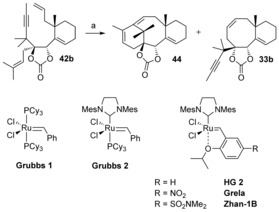		
Precatalyst	Yield of **44** [%]	Yield of **33 b** [%]
Grubbs 1	0^[b]^	0
Grubbs 2	45	45
Hoveyda–Grubbs 2	59	38
Grela	55	45
**Zhan‐1 B**	**70**	**20**

[a] Reaction conditions: a) 10 mol % precatalyst, toluene, 5×10^−3^ m, 110 °C. [b] No reaction was observed.

**Scheme 12:**
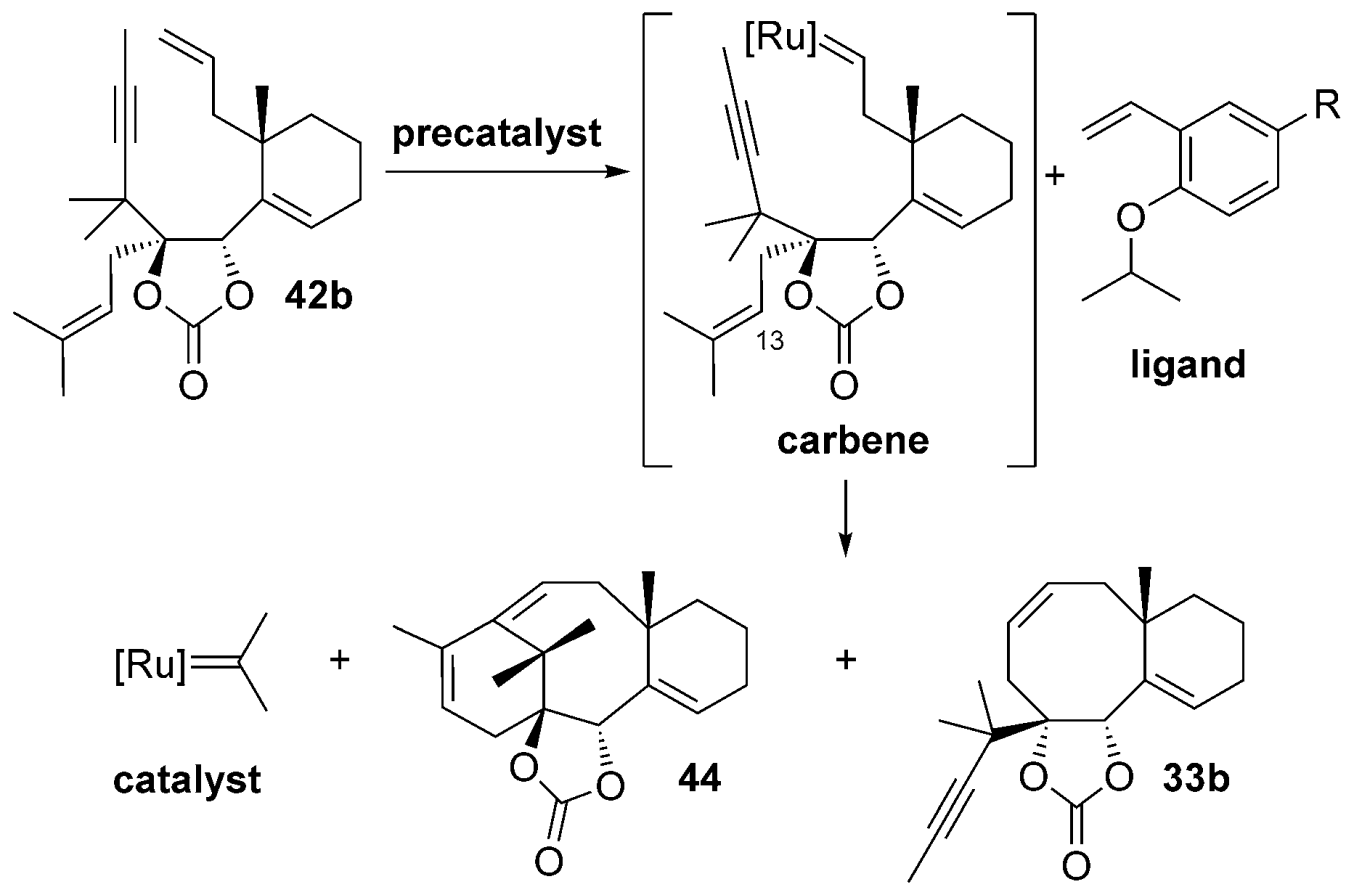
Metathesis scheme. Conditions: toluene, reflux.

The different ratios observed with the Hoveyda–Grubbs‐type precatalysts cannot be easily rationalized. Indeed, metathesis of substrate **42 b** with any **precatalyst** will result in the same **carbene** (Scheme 12). This intermediate should then lead to the same ratios of **44** and **33 b** after cyclization, releasing the same isopropylidene **catalyst**. The only difference between the reactions is the **ligand** released after the first catalytic cycle, which could recombine with the isopropylidene catalyst to reform the **precatalyst**. To probe the influence of the ligand, a metathesis experiment was run with 10 mol % of the Hoveyda–Grubbs 2 precatalyst and 300 mol % of the corresponding ligand, but the observed ratio of **44** and **33 b** was very similar to the one observed without any added ligand.12

**Scheme 13:**
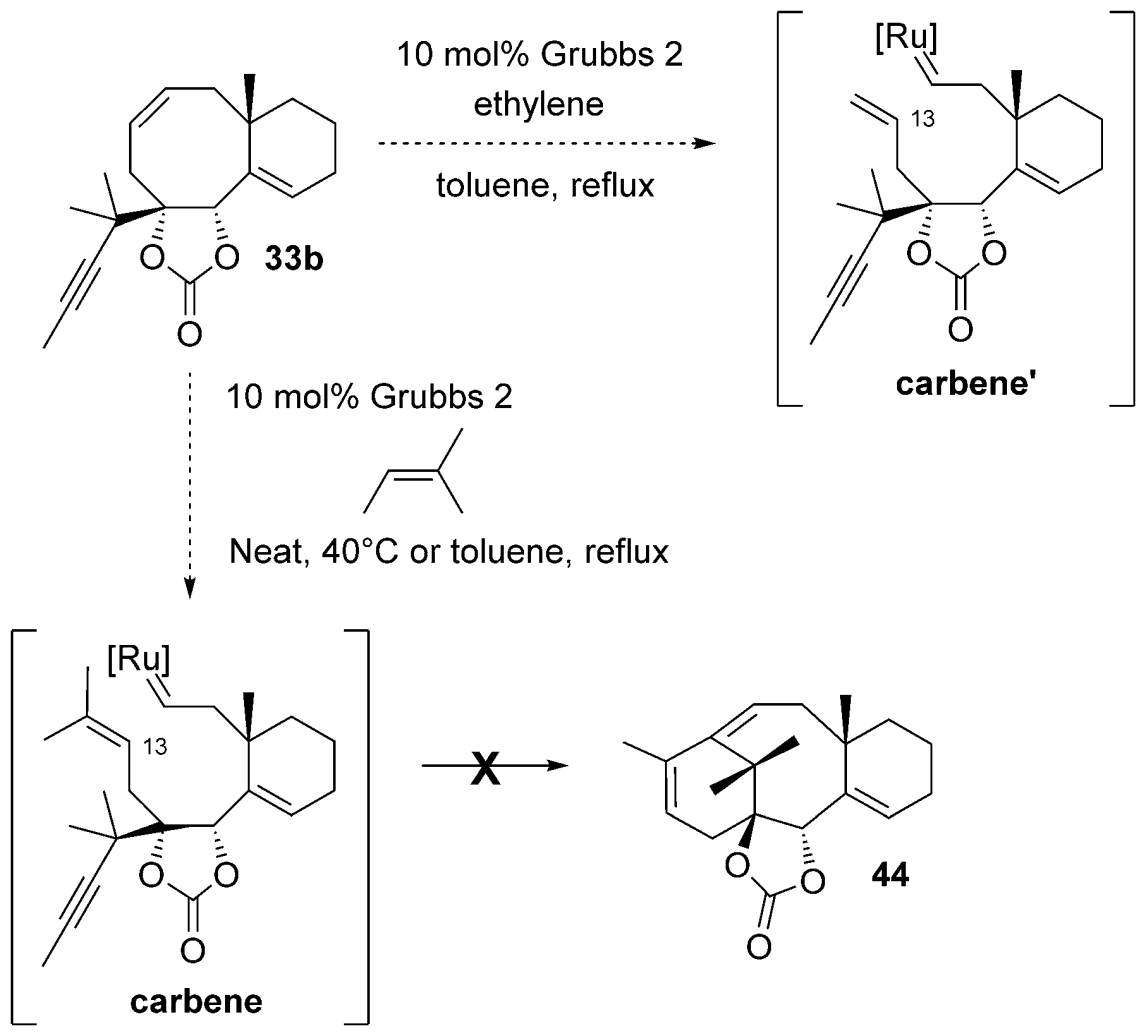
Equilibration attempts.

Attempts to convert bicyclic product **33 b** to the desired tricycle **44** by heating it in toluene at reflux in the presence of the Grubbs 2 or Zhan‐1B precatalyst were unsuccessful, even under microwave conditions. When 10 mol % of precatalyst was used, only **33 b** was recovered, and with 100 mol % of precatalyst decomposition occurred. Ring opening of **33 b** in the presence of ethylene was not considered, because it would lead to a carbene unsubstituted at C13, **carbene’** (Scheme 13), which would undergo diene metathesis preferentially. In an effort to regenerate the **carbene** with a trisubstituted olefin at C13, bicycle **33 b** was submitted to the above conditions in the presence of 2‐methyl‐2‐butene, the reagent which Grubbs and co‐workers have employed for the synthesis of trisubstituted olefins from their terminal homologues by cross metathesis,^39^ but to no avail (Scheme 13). These results seem to indicate that the formation of compound **33 b** is not reversible, and that the metathesis reactions leading to **33 b** and **44** are under kinetic control.13

## Conclusions

In summary, we have synthesized Taxol analogues with an unprecedented skeleton as well as the tricyclic core of Taxol in a very efficient fashion. The key step in these syntheses is a cascade ring‐closing dienyne metathesis (RCDEYM) reaction, leading to either 14,15‐isotaxane tricyclic ring systems or the tricylic ring system of Taxol in one operation from simple precursors, by judicious choice of the position of the alkyne (C13 for isotaxanes or C11 for taxanes). Furthermore, in the case of the taxane synthesis, we have shown that we can direct the course of the crucial metathesis reaction by adding a temporary methyl substituent to the olefin at C13, which does not appear in the structure of the tricycle. Calculations rationalizing the different outcomes of the metathesis reactions of compounds **42 a**,**b** and **43 a**,**b**, which strongly depend on the stereochemistry and the protecting group of the diol at the C1 and C2 positions in the metathesis precursors, will be reported in due course.

## Experimental Section

All experimental details can be found in the Supporting Information. The material includes compound characterization, crystal structures of **16 b** and **33 a**, and copies of spectra for all new compounds.

## Supporting information

As a service to our authors and readers, this journal provides supporting information supplied by the authors. Such materials are peer reviewed and may be re‐organized for online delivery, but are not copy‐edited or typeset. Technical support issues arising from supporting information (other than missing files) should be addressed to the authors.

Supplementary
