# Supplementary material for: Cascade Metathesis Reactions for the Synthesis of Taxane and Isotaxane Derivatives
Source: Chemistry. 2016 Apr 8;22(20):6891–8. doi: 10.1002/chem.201600592 (PMC4982030; doi:10.1002/chem.201600592)
Supplement: Supplementary file 1 — Supplementary [file CHEM-22-6891-s001.pdf]

CHEMISTRY
A European Journal

Supporting Information

Cascade Metathesis Reactions for the Synthesis of Taxane and Isotaxane Derivatives

Cong Ma,[b] Aurélien Letort,[a] Rémi Aouzal,[b] Antonia Wilkes,[a] Gourhari Maiti,[b]
Louis J. Farrugia,[a] Louis Ricard,[c] and Joëlle Prunet*[a]

chem_201600592_sm_miscellaneous_information.pdf

Table of Contents

1. General Information S2

2. Experimental Procedures and Characterisation Data of New Compounds S3

3. X-ray Data of **16b** and **33a** S18

4. NMR Spectra of New Compounds S21

1. General Information

All air and/or water sensitive reactions were carried out under an argon atmosphere with dry, freshly distilled solvents using standard syringe-cannula/septa techniques. All corresponding glassware was carefully dried under vacuum with a flameless heat gun. Melting points were determined on a REICHERT apparatus or a Stuart scientific-Melting Point SMP1 apparatus and are uncorrected. NMR spectra were recorded on a BRUKER AM 400 or a BRUKER Avance 400 instrument (^{1}H NMR at 400 MHz and ^{13}C NMR at 100.6 MHz) or a Bruker DPX-500 spectrometer (1H NMR at 500 MHz and 13C NMR at 126 MHz). Chemical shifts are reported in ppm and referenced to residual $CHCl_3$ (δ = 7.27 for ^{1}H NMR, δ = 77.00 for ^{13}C NMR). Signals in NMR spectra are described as singlet (s), doublet (d), triplet (t), quartet (q), quintet (quint), septet (sept), multiplet (m), broad (br) or combination of these, which refers to the spin-spin coupling pattern observed. Spin-spin coupling constants reported are uncorrected. Two-dimensional (COSY, HSQC, HMBC, NOESY) NMR spectroscopy was used where appropriate to assist the assignment of signals in the ^{1}H and ^{13}C NMR spectra. IR spectra were obtained on a PERKIN-ELMER FT 1600 instrument or a Shimadzu FTIR-8400 instrument with a Golden Gate™ attachment. Mass spectra (MS) were obtained on a HEWLETT-PACKARD HP 5989B spectrometer via either direct injection or GC/MS coupling with a HEWLETT-PACKARD HP 5890 chromatograph. Ionization was obtained either by electron impact (EI) or chemical ionization with ammonia (CI, NH_3) or methane (CI, CH_4). High-resolution mass spectra were performed with a Jeol GC Mate II apparatus, by direct introduction of the compound, in magnet mode or were recorded under FAB, ESI and CI conditions by the analytical services at the University of Glasgow. Flash column chromatography was performed performed using silica gel 60, 230-400 mesh. Thin Layer Chromatography (TLC) was performed on precoated plates of silica gel 60F, and developed under UV-light and/or with 7-10% ethanolic phosphomolybdic acid solution, anisaldehyde solution, ceric ammonium molybdate solution, vanillin/sulphuric acid or $KMnO_4$-solution as developing agents. Optical rotations were determined on a Perkin-Elmer 241-instrument operating at the D-line of Na. X-Ray structures were solved at the DCPH, Ecole Polytechnique, using a Nonius KappaCCD diffractometer, φ and ω scans, Mo$\kappa\alpha$ radiation (λ = 0.71069 Å), graphite monochromator, T = 150 K, structure solution with SIR97 and refined in SHELXL-97 by full matrix least-squares using anisotropic thermal displacement parameters for all-non hydrogen atoms.

2. Experimental Procedures and Characterisation Data

3,3-Dimethyl-oct-1-en-6-yn-4-ol (10)

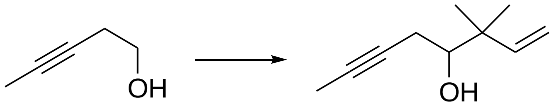

10

Formula: $C_{10}H_{16}O_2$

MW: 152.2

To a solution of Dess-Martin periodinane (18.2 g, 43.0 mmol, 1.10 equiv) in dichloromethane (100 ml) was added a solution of 3-pentynol (3.6 mL, 39.0 mmol) in dichloromethane (20 mL) and a drop of water. After stirring for 1 h, the reaction was cooled to -78 °C and pentane (50 mL) was added. The mixture was filtered through a pad of silica gel, and the crude product was concentrated in vacuo at room temperature. Freshly prepared 1-bromo-3-methylbut-2-ene (4.97 mL, 43.0 mmol, 1.10 equiv) was added to a solution of the crude product in THF (50 mL). This was followed by the addition of aqueous ammonium chloride (sat., 100 mL). The resulting mixture was cooled to 0°C, and activated zinc dust (7.60 g, 0.117 mol, 3.00 equiv) was added slowly. The mixture was then stirred vigorously at room temperature for 1 h (no inert atmosphere is necessary). After completion of the reaction, the mixture was filtered through a pad of celite, the two phases were separated and the aqueous phase was extracted with diethyl ether (3x25 mL). The combined organic fractions were washed with brine (20 mL), dried over magnesium sulfate, filtered and the solvent was removed under reduced pressure. Purification of the crude product by flash chromatography (diethyl ether/petroleum ether: 10/90) afforded alcohol **10** (5.92 g, 99%) as a colorless oil.

^{1}H NMR (400 MHz, $CDCl_3$): δ = 5.82 (dd, J = 17.5, 10.9 Hz, 1H), 5.03-4.97 (m, 2H), 3.41 (dt, J = 9.5, 2.9 Hz, 1H), 2.33 (dq, J = 16.3, 2.5 Hz, 1H), 2.16-2.07 (m, 2H), 1.76 (t, J = 2.5 Hz, 3H), 1.00 (s, 3H), 0.99 (s, 3H) ppm.

^{13}C NMR (100 MHz, $CDCl_3$): δ = 144.42, 112.8, 77.8, 76.5, 76.4, 40.8, 22.9, 22.8, 22.4, 3.4 ppm.

IR (film): 3583, 3083, 2967, 2922, 2869, 1639, 1466, 1420, 1387, 1272, 1192, 1072, 918, 688, 538 cm^{-1}.

MS (CI): m/z 153 [MH$^+$].

3,3-Dimethyloct-1-en-6-yn-4-one (11)

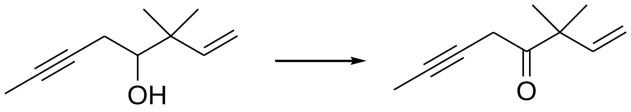

11

Formula: $C_{10}H_{14}O$

MW: 150.2

To a solution of Dess-Martin periodinane (5.97 g, 14.1 mmol, 1.20 equiv) in dichloromethane (50 mL) was added a solution of alcohol **10** (610 mg, 11.7 mmol) in dichloromethane (10 mL) and a drop of water. After stirring for 1 h, the reaction was cooled to -78 °C and pentane (50 mL) was added. The mixture was filtered through a pad of silica gel, and the crude product was concentrated in vacuo and purified by flash chromatography (diethyl ether/petroleum ether: 5/95) to afford ketone **11** (570 mg, 95% yield) as a pale yellow oil.

^{1}H NMR (400 MHz, $CDCl_3$): δ = 5.86 (dd, J = 17.7, 10.6 Hz, 1H), 5.14-5.10 (m, 2H), 3.30 (q, J = 2.5 Hz, 2H), 1.77 (t, J = 2.5 Hz, 3H), 1.20 (s, 3H), 1.20 (s, 3H) ppm.

^{13}C NMR (100 MHz, $CDCl_3$): δ = 206.5, 141.7, 114.6, 79.6, 71.3, 50.5, 29.0, 23.3, 3.3 ppm.

IR (film): 3086, 2959, 2250, 1719, 1635, 1452, 1322, 1240, 1171, 1078, 923, 673, 561 cm^{-1}.

HRMS (EI) **Calcd** for $C_{10}H_{14}O$: 150.1045 **Found**: 150.1048.

2-(1,1-Dimethylallyl)-2-trimethylsilanyloxy-hex-4-ynal (9)

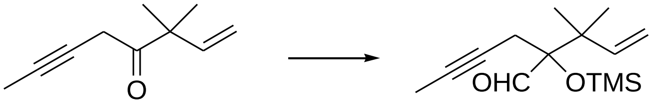

9

Formula: $C_{14}H_{24}O_2Si$

MW: 252.4

To a solution of ketone **11** (800 mg, 5.33 mmol) and DABCO (300 mg, 2.67 mmol, 0.50 equiv) in dichloromethane (10 mL) was added TMSCN (0.850 mL, 6.40 mmol, 1.20 equiv). The resulting mixture was refluxed for 2 h. The solvents and excess TMSCN were then evaporated under reduced pressure (with a NaOCl/NaOH trap). Purification of the crude product by flash chromatography (diethyl ether/petroleum ether: 2/100 then 5/100) afforded the desired racemic nitrile compound (1.29 g, 97 %) as a pale yellow oil. The intermediate nitrile (220 mg, 0.88 mmol) was dissolved in dichloromethane (10 mL) and cooled to -78°C. DIBAL-H (2.21 mL, 1 M in hexanes, 2.21 mmol, 2.50 equiv) was then added dropwise. The reaction mixture was warmed to 0°C and stirred for 2 h. The reaction was quenched by addition of ethyl acetate (4

mL), diluted with diethyl ether (10 mL) and allowed to warm to room temperature. Silica (10 g) was added to the solution, which was then placed at -20°C overnight. After completion of the reaction, the mixture was warmed to room temperature and the silica was filtered off. Solvents were evaporated under reduced pressure. Purification of the crude product by flash chromatography (diethyl ether/petroleum ether: 1/99) afforded racemic aldehyde **9** (134 mg, 60%) as a colorless oil.

^{1}H NMR (400 MHz, $CDCl_3$): δ = 9.64 (s, 1H), 5.96 (dd, *J* = 17.5, 10.9 Hz, 1H), 5.08-4.99 (m, 2H), 2.75 (dd, *J* = 16.8, 2.6 Hz, 1H), 2.38 (dd, *J* = 16.8, 2.6 Hz, 1H), 1.72 (t, *J* = 2.6 Hz, 3H), 1.03 (s, 3H), 1.03 (s, 3H), 0.21 (s, 9H) ppm.

^{13}C NMR (100 MHz, $CDCl_3$): δ = 204.1, 143.1, 113.7, 86.3, 79.3, 75.2, 44.5, 23.6, 22.3, 22.0, 3.6, 2.4 ppm.

IR (film): 3055, 2964, 2928, 1735, 1463, 1422, 1253, 1153, 1009, 967, 926, 894, 857, 563, 502 cm^{-1}.

HRMS (EI) **Calcd** for $C_{14}H_{24}O_2Si$: 252.1546. **Found**: 252.1545.

(1*R*,2*R*)-2-(1,1-Dimethylallyl)-1-[(*S*)-6-methyl-6-(3-trityloxypropyl)cyclohex-1-enyl]hex-4-yne-1,2-diol (14a) and

(1*S*,2*S*)-2-(1,1-Dimethylallyl)-1-[(*S*)-6-methyl-6-(3-trityloxypropyl)cyclohex-1-enyl]hex-4-yne-1,2-diol (14b)

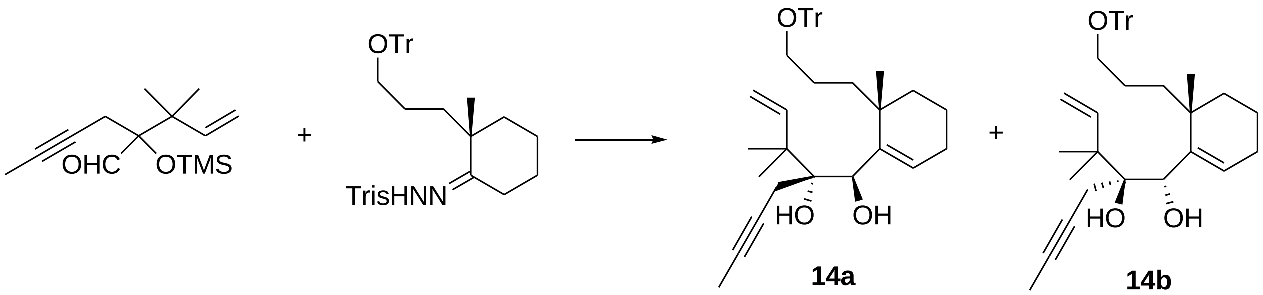

14a **14b**

tert-Butyllithium (2.65 mL, 3.98 mmol, 2.30 equiv, titrated 1.50 M) was added dropwise to a solution of hydrazone **13** (1.20 g, 1.73 mmol) in THF (10 mL) at -78°C. The solution turned red and was stirred at -78°C for 30 min until the color turned dark red, then the temperature was allowed to warm to 0°C. When nitrogen evolution was finished and the color had turned to red, the solution was cooled again to -78°C. A solution of titrated $CeCl_3$ (426 mg, 1.73 mmol, 1.00 equiv) with *tert*-Butyllithium in THF (2 mL) was then added and stirred for 30 min. A cooled solution of aldehyde **9** (480 mg, 1.90 mmol, 1.10 equiv) in THF (5.0 mL) was added dropwise *via* cannula. The resulting mixture was stirred at -78°C for 30 min. The reaction was quenched by addition of aqueous sodium hydrogencarbonate (sat., 10 mL). The phases were separated then the aqueous phase was extracted with diethyl ether (3x15 mL). The combined organic fractions

were washed with brine (20 mL) and dried over magnesium sulfate, filtered and the solvent was removed under reduced pressure. Aqueous 1N HCl (3.46 mL, 3.46 mmol, 2.00 equiv) was added at 0°C to a solution of the crude product in THF (5.0 mL). The resulting mixture was allowed to warm to room temperature over 1 h and stirred overnight. The reaction was quenched by addition of aqueous sodium hydrogencarbonate (sat., 3 mL). The phases were separated and the aqueous phase was extracted with diethyl ether (3x5 mL). The combined organic fractions were washed with brine (5 mL), dried over magnesium sulfate, filtered and the solvent was removed under reduced pressure. Purification of the crude product by flash chromatography (diethyl ether/petroleum ether: 10/90) allowed separation of the two diastereomers to afford the desired diols diastereomer **14a** (438 mg, 44% yield over two steps) and diastereomer **14b** (410 mg, 41% yield) as colorless oils (85% global yield).

(1*R*,2*R*)-2-(1,1-Dimethylallyl)-1-[(*S*)-6-methyl-6-(3-trityloxypropyl)cyclohex-1-enyl]hex-4-yne-1,2-diol (14a)

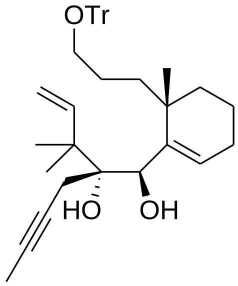

Formula: $C_{40}H_{48}O_3$

MW: 576.8

[α]$_D^{20}$: +100.7 (*c* 1.5, CH_2Cl_2).

^{1}H NMR (400 MHz, $CDCl_3$): δ = 7.50-7.48 (m, 6H), 7.33-7.30 (m, 6H), 7.26-7.23 (m, 3H), 6.30-6.23 (m, 2H), 5.09-5.05 (m, 2H), 4.26 (d, *J* = 6.1 Hz, 1H), 3.17 (s, 1H), 3.13-3.08 (m, 2H), 2.59-2.49 (m, 2H), 2.19 (d, *J* = 6.1 Hz, 1H), 2.12-2.00 (m, 2H), 1.80 (t, *J* = 2.6 Hz, 3H), 1.71-1.56 (m, 6H), 1.52-1.47 (m, 2H), 1.24 (s, 3H), 1.22 (s, 3H), 1.02 (s, 3H) ppm.

^{13}C NMR (100 MHz, $CDCl_3$): δ = 146.6, 146.1, 144.4, 128.6, 128.3, 127.6, 126.7, 112.4, 86.4, 79.2, 77.2, 76.4, 68.9, 64.4, 46.4, 37.1, 36.6, 35.2, 26.0, 26.0, 25.8, 24.6, 23.7, 23.2, 18.7, 3.6 ppm.

IR (film): 3602, 3533, 3062, 2933, 2868, 1956, 1632, 1597, 1487, 1150, 1383, 1221, 1115, 1080, 922, 900, 802, 719, 524 cm^{-1}.

HRMS (EI) **Calcd** for $C_{40}H_{48}O_3$: 576.3604. **Found**: 576.3599.

(1*S*,2*S*)-2-(1,1-Dimethylallyl)-1-[(*S*)-6-methyl-6-(3-trityloxypropyl)cyclohex-1-enyl]hex-4-yne-1,2-diol (14b)

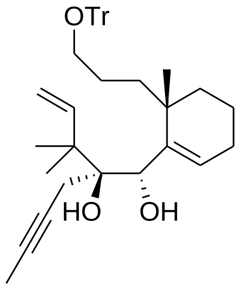

Formula: $C_{40}H_{48}O_3$

MW: 576.8

[α]D20: +137.5 (*c* 1.6, CH_2Cl_2).

^{1}H NMR (400 MHz, $CDCl_3$): δ = 7.45-7.43 (m, 6H), 7.32-7.28 (m, 6H), 7.25-7.21 (m, 3H), 6.26-6.17 (m, 2H), 5.04-4.96 (m, 2H), 4.24 (d, *J* = 6.1 Hz, 1H), 3.09 (s, 1H), 3.05 (t, *J* = 6.9 Hz, 2H), 2.55-2.43 (m, 2H), 2.07-2.03 (m, 3H), 1.67 (t, *J* = 2.4 Hz, 3H), 1.61-1.51 (m, 4H), 1.41-1.33 (m, 4H), 1.18 (s, 3H), 1.16 (s, 3H), 1.08 (s, 3H) ppm.

^{13}C NMR (100 MHz, $CDCl_3$): δ = 147.2, 146.6, 144.4, 128.6, 127.7, 127.6, 126.8, 112.5, 86.3, 79.3, 77.2, 76.4, 69.5, 64.4, 46.4, 36.6, 35.7, 34.9, 26.1, 25.8, 25.0, 24.4, 23.7, 23.3, 18.5, 3.7 ppm.

IR (film): 3604, 3535, 3062, 2930, 2866, 1490, 1450, 1380, 1222, 1073, 1029, 878.6, 802.2, 724, 626, 533 cm^{-1}.

HRMS (EI) **Calcd** for $C_{40}H_{48}O_3$: 576.3604. **Found**: 576.3605.

(4*R*,5*R*)-5-((*S*)-6-Allyl-6-methylcyclohex-1-enyl)-4-but-2-ynyl-4-(1,1-dimethylallyl)-[1,3]dioxolan-2-one (15a) and

(4*S*,5*S*)-5-((*S*)-6-Allyl-6-methylcyclohex-1-enyl)-4-but-2-ynyl-4-(1,1-dimethylallyl)-[1,3]dioxolan-2-one (15b)

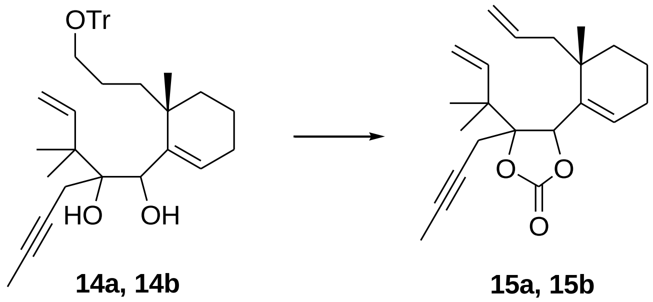

14a, 14b **15a, 15b**

Diol **14a** (126 mg, 0.220 mmol) was dissolved in MeOH (5 mL) at 20°C. Amberlyst H-15 (40 mg) was then added, and the mixture was stirred overnight at 20°C. The resin was then filtered

off and the solvents were removed *in vacuo*. *o*-Nitrophenylselenocyanate (61.0 mg, 0.269 mmol, 2.40 equiv) was added in one portion at 20°C to a solution of the crude product (60.0 mg, 0.180 mmol) in THF (1.0 mL). Tributylphosphine (0.067 mL, 0.269 mmol, 2.40 equiv) was then added dropwise. The resulting mixture was stirred at 20°C for 20 min. The completion of the reaction was checked by TLC. Water was then added, and the solution was diluted with diethyl ether. The phases were separated then the aqueous phase was extracted with diethyl ether. The combined organic fractions were washed with brine, dried over magnesium sulfate, filtered and the solvent was removed under reduced pressure. The crude product was used for the next step without purification. Carbonyl diimidazole (291 mg, 1.80 mmol, 10.0 equiv) was added at 20°C to a solution of the crude product in toluene (5 mL). The resulting mixture was refluxed for three days. After cooling, the reaction was quenched by addition of saturated aqueous sodium hydrogen carbonate. The phases were separated then the aqueous phase was extracted with ether. The combined organic fractions were washed with brine, dried over magnesium sulfate, filtered and the solvent was removed under reduced pressure. The crude product was then dissolved in THF (1.0 mL). Premixed aqueous hydrogen peroxide (1.5 mL, 10% wt solution in water) with ammonium molybdate (7 M) was added dropwise at -10°C. The mixture was then stirred for 5 min. Water was then added, and the solution was diluted with diethyl ether. The phases were separated then the aqueous phase was extracted with diethyl ether. The combined organic fractions were washed with brine and dried over magnesium sulfate, filtered and the solvent was removed under reduced pressure. Purification of the crude product by flash chromatography (diethyl ether/petroleum ether: 10/90) afforded the desired diene **15a** (56 mg, 75% yield over four steps) as a pale yellow oil.

The same procedure repeated with 68 mg of diol **14b** afforded the desired diene **15b** (26 mg, 65% yield over four steps) as a pale yellow oil.

(4*R*,5*R*)-5-((*S*)-6-Allyl-6-methylcyclohex-1-enyl)-4-but-2-ynyl-4-(1,1-dimethylallyl)-[1,3]dioxolan-2-one (15a)

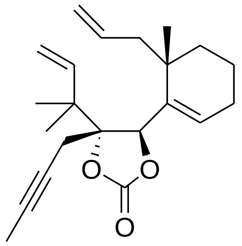

Formula: $C_{22}H_{30}O_3$

MW: 342.5

[α]$_D^{20}$: +19.0 (*c* 1.6, CH_2Cl_2).

^{1}H NMR (400 MHz, $CDCl_3$): δ = 6.03 (ddd, *J* = 17.8, 10.5, 1.5 Hz, 1H), 5.95 (dt, *J* = 3.9, 1.5 Hz, 1H), 5.82-5.72 (m, 1H), 5.25-5.20 (m, 2H), 5.09-5.01 (m, 3H), 2.74 (dq, *J* = 17.3, 2.3 Hz, 2H), 2.27 (dd, *J* = 13.9, 6.7 Hz, 1H), 2.16-2.10 (m, 3H), 1.75-1.73 (m, 3H), 1.66-1.57 (m, 2H), 1.43-1.33 (m, 2H), 1.23 (d, *J* = 1.5 Hz, 3H), 1.22 (s, 3H), 1.01 (d, *J* = 1.5 Hz, 3H) ppm.

^{13}C NMR (100 MHz, $CDCl_3$): δ = 141.9, 138.5, 134.0, 133.3, 118.0, 115.9, 89.0, 79.1, 77.7, 74.0, 46.2, 45.0, 36.9, 35.6, 26.0, 25.6, 23.4, 22.9, 21.2, 18.0, 3.7 ppm.

IR (film): 3078, 2930, 2870, 1807, 1640, 1520, 1459, 1424, 1334, 1176, 1043, 923, 815, 639, 464 cm^{-1}.

HRMS (EI) **Calcd** for $C_{22}H_{30}O_3$: 342.2195. **Found**: 342.2209.

(4*S*,5*S*)-5-((*S*)-6-Allyl-6-methylcyclohex-1-enyl)-4-but-2-ynyl-4-(1,1-dimethylallyl)-[1,3]dioxolan-2-one (15b)

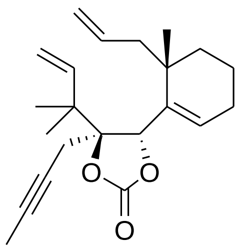

Formula: $C_{22}H_{30}O_3$

MW: 342.5

$[\alpha]_D^{20}$: -66.7 (*c* 1.0, CH_2Cl_2).

^{1}H NMR (400 MHz, $CDCl_3$): δ = 6.07 (dd, *J* = 17.2, 11.0 Hz, 1H), 5.91 (t, *J* = 4.0 Hz, 1H), 5.80-5.70 (m, 1H), 5.26-5.21 (m, 2H), 5.08-5.00 (m, 3H), 2.76 (dq, *J* = 17.3, 2.5 Hz, 2H), 2.21 (dd, *J* = 13.5, 7.6 Hz, 1H), 2.15-2.10 (m, 2H), 2.07 (dd, *J* = 13.5, 7.0 Hz, 1H), 1.75 (t, *J* = 2.5 Hz, 3H), 1.69-1.57 (m, 3H), 1.38-1.32 (m, 1H), 1.43-1.33 (m, 1H), 1.25 (s, 3H), 1.23 (s, 3H), 1.08 (s, 3H) ppm.

^{13}C NMR (100 MHz, $CDCl_3$): δ = 154.7, 141.9, 138.5, 134.0, 133.3, 118.0, 115.9, 89.0, 79.1, 77.7, 74.0, 46.2, 45.0, 36.9, 35.6, 26.0, 25.6, 23.4, 22.9, 21.2, 18.0, 3.7 ppm.

IR (film): 3078, 2966, 2933, 2867, 1809, 1639, 1459, 1425, 1175, 1042, 873, 678, 563 cm^{-1}.

HRMS (EI) **Calcd** for $C_{22}H_{30}O_3$: 342.2195. **Found**: 342.2203.

(1*R*,5*R*,11*S*)-11,15,17,17-Tetramethyl-2,4-dioxatetracyclo[12.3.1.0]octadeca-6,13,15-trien-3-one (16a)

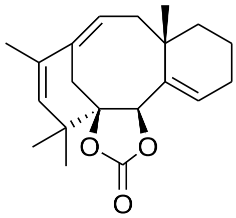

Formula: $C_{20}H_{26}O_3$

MW: 314.4

Diene **15a** (30 mg, 95 µmol) was dissolved in toluene (5.0 mL). The mixture was thoroughly degassed three times while stirring. Second-generation Grubbs' catalyst (4.0 mg, 9.5 µmol, 10 mol%) was added and the mixture was refluxed for 12 h. After cooling, the solvent was evaporated under reduced pressure. Purification of the crude product by flash chromatography (diethyl ether/petroleum ether: 10/90) afforded **16a** (17 mg, 62% yield) as a pale yellow oil.

$[\alpha]_D^{20}$: +104.8 (*c* 0.5, CH_2Cl_2).

^{1}H NMR (400 MHz, $CDCl_3$): δ = 5.71 (t, *J* = 3.8 Hz, 1H), 5.65 (t, *J* = 8.2 Hz, 1H), 5.13 (s, 1H), 4.98 (s, 1H), 3.11 (d, *J* = 12.5 Hz, 1H), 2.72 (dd, *J* = 14.4, 8.0 Hz, 1H), 2.66 (d, *J* = 12.5 Hz, 1H), 2.15 (dd, *J* = 14.4, 7.7 Hz, 1H), 2.12-2.00 (m, 2H), 1.72 (s, 3H), 1.68-1.55 (m, 4H), 1.27 (s, 3H), 1.25 (s, 3H), 1.15 (s, 3H) ppm.

^{13}C NMR (100 MHz, $CDCl_3$): δ = 153.6, 137.6, 136.9, 136.1, 132.3, 131.5, 127.8, 90.9, 88.0, 43.2, 41.4, 40.9, 32.7, 31.9, 29.7, 27.5, 26.0, 23.6, 18.1, 17.5 ppm.

IR (film): 2929, 2858, 1809, 1462, 1261, 1191, 1052, 876, 826, 787, 749, 729, 679, 572 cm^{-1}.

HRMS (EI) **Calcd** for $C_{20}H_{26}O_3$: 314.1882. **Found**: 314.1881.

(*Z*)-(2*R*,6*R*,11*R*)-6-(1,1-Dimethyl-prop-2-enyl)-8-ethenyl-11-methyl-3,5-dioxa-tricyclo[9.4.0.0^{2,6}]pentadeca-1(15),8-dien-4-one (16a')

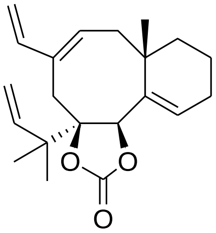

Formula: $C_{21}H_{28}O_3$

MW: 328.5

^{1}H NMR (400 MHz, $CDCl_3$): δ = 6.99-6.87 (m, 3H), 6.51-6.41 (m, 1H), 4.85 (s, 1H), 4.50 (m, 2H), 4.12 (m, 2H), 2.59-2.50 (m, 2H), 2.35-2.29 (m, 2H), 2.27-2.18 (m, 2H), 1.78-1.60 (m, 4H), 1.55 (s, 3H), 1.44 (s, 3H), 1.26 (s, 3H) ppm.

(1*S*,5*S*,11*S*)-11,15,17,17-Tetramethyl-2,4-dioxatetracyclo[12.3.1.0]octadeca-6,13,15-trien-3-one (16b)

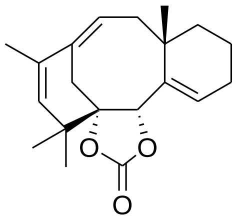

Formula: $C_{20}H_{26}O_3$

MW: 314.4

Diene **15b** (20 mg, 58 μmol) was dissolved in toluene (5.0 mL). The mixture was thoroughly degassed three times while stirring. Second-generation Grubbs' catalyst (2.5 mg, 2.9 μmol, 5 mol%) was added and the mixture was stirred for 1 h at 80°C. After cooling, the solvent was evaporated under reduced pressure. Purification of the crude product by flash chromatography (diethyl ether/petroleum ether: 10/90) afforded **16b** (17 mg, 91% yield) as a pale yellow oil.

$[\alpha]_D^{20}$: +34.4 (*c* 1.7, CH_2Cl_2).

^{1}H NMR (400 MHz, $CDCl_3$): δ = 5.77 (t, *J* = 8.3 Hz, 1H), 5.73 (t, *J* = 3.8 Hz, 1H), 4.97 (d, *J* = 1.0 Hz, 1H), 4.92 (s, 1H), 2.84 (d, *J* = 11.3 Hz, 1H), 2.77 (dd, *J* = 13.0, 8.7 Hz, 1H), 2.50 (d, *J* = 11.3 Hz, 1H), 2.29-2.20 (m, 2H), 1.86-1.83 (m, 1H), 1.81 (d, *J* = 1.0 Hz, 3H), 1.78-1.75 (m, 1H), 1.70-1.58 (m, 2H), 1.48-1.43 (m, 1H), 1.20 (s, 3H), 1.09 (s, 3H), 0.97 (s, 3H) ppm.

^{13}C NMR (100 MHz, $CDCl_3$): δ = 156.3, 139.6, 136.6, 132.7, 131.8, 128.5, 125.1, 91.9, 79.2, 43.1, 42.5, 41.5, 38.6, 30.7, 26.8, 26.1, 24.4, 24.2, 17.9, 17.4 ppm.

IR (film): 2973, 2931, 2864, 2253, 1804, 1732, 1461, 1379, 1261, 1153, 1119, 1044, 905, 856, 747, 639, 574 cm^{-1}.

HRMS (EI) **Calcd** for $C_{20}H_{26}O_3$: 314.1882. **Found**: 314.1881.

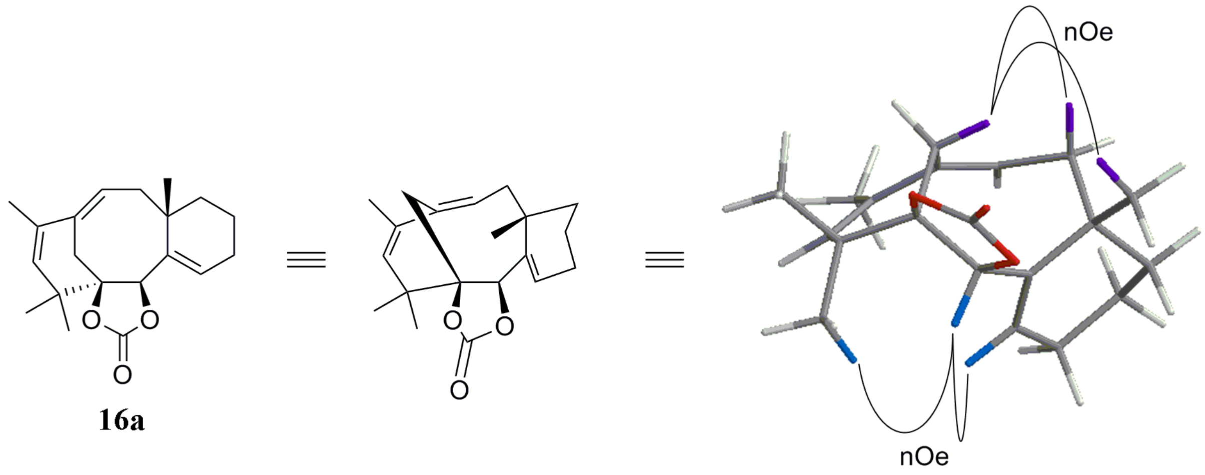

16a

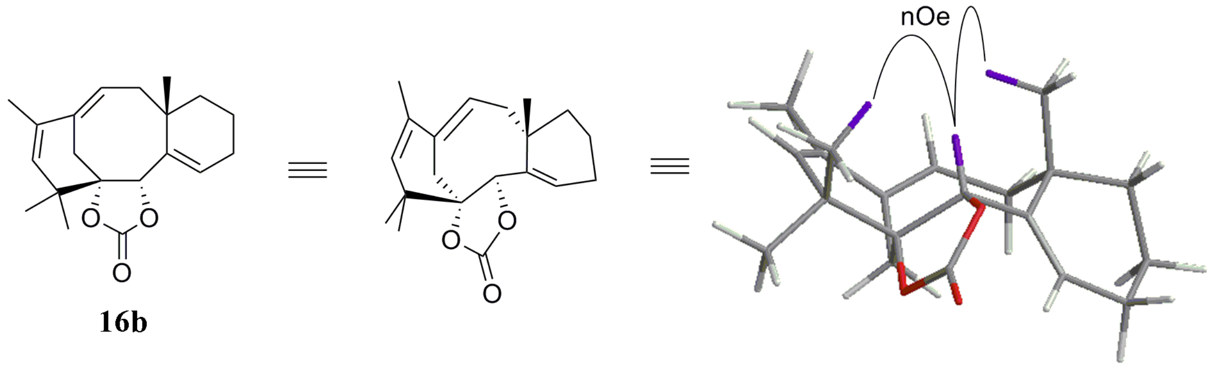

16b

Figure S1: Energy minimized models of **16a** and **16b** using Chem3D Pro 8.0. The Nuclear Overhauser Effect (NOE) of hydrogen nuclei spatially close to each other can be observed through 2D-NOESY spectra.

(1*R*,2*R*)-2-Hydroxy-1-[(*S*)-6-allyl-6-methylcyclohex-1-enyl]- 2-(1,1-dimethylallyl)hex-4-yn-1-yl benzoate (17a)

(1*S*,2*S*)-2-Hydroxy-1-[(*S*)-6-allyl-6-methylcyclohex-1-enyl]- 2-(1,1-dimethylallyl)hex-4-yn-1-yl benzoate (17b)

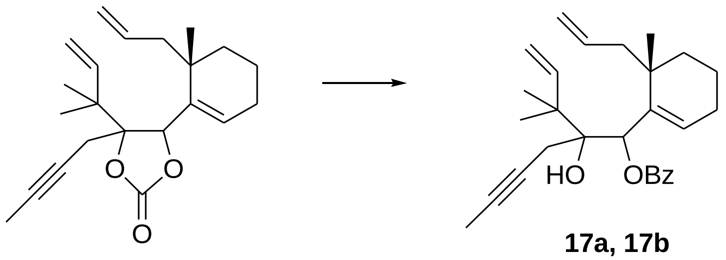

16a, 16b **17a, 17b**

To a solution of **16a** (46 mg, 0.13 mmol) in tetrahydrofuran (3 mL) at -78˚C was added phenyllithium (1.72 mL, 0.7 M in ether, 1.17 mmol, 9.0 equiv). The mixture was stirred at this temperature for 1.5 h before being quenched with saturated aqueous sodium hydrogen carbonate. The aqueous phase was extracted with diethyl ether and the combined organic extracts were washed with brine, dried over anhydrous magnesium sulfate, filtered and concentrated in vacuo. Purification by flash column chromatography (diethyl ether/petroleum ether: 5/95) gave **17a** as a pale yellow oil (30 mg, 54 %).

The same procedure was used with **16b** (42 mg, 0.12 mmol) and gave the desired benzoate **17b** as a pale yellow oil (36 mg, 70%).

(1*R*,2*R*)-2-Hydroxy-1-[(*S*)-6-allyl-6-methylcyclohex-1-enyl]- 2-(1,1-dimethylallyl)hex-4-yn-1-yl benzoate (17a)

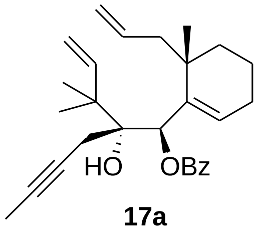

17a

Formula: $C_{28}H_{36}O_3$

MW: 420.6

[α]D^{25}: +65.0 (*c* 0.6, CH_2Cl_2).

^{1}H NMR (400 MHz, $CDCl_3$): δ = 8.00 (d, *J* = 7.5 Hz, 2H), 7.54 (t, *J* = 7.5 Hz, 1H), 7.43 (t, *J* = 7.5 Hz, 2H), 6.44 (t, *J* = 4.0 Hz, 1H), 6.09 (dd, *J* = 17.7, 10.8 Hz, 1H), 5.81 (s, 1H), 5.74-5.62 (m, 1H), 5.01-4.92 (m, 2H), 4.89 (d, *J* = 17.7 Hz, 1H), 4.71 (d, *J* = 10.7 Hz, 1H), 2.89 (s, 1H), 2.67 (dq, *J* = 17.0, 2.6 Hz, 1H), 2.56 (dq, *J* = 17.0, 2.6 Hz, 1H), 2.46 (m, 1H), 2.16-2.07 (m, 3H), 1.82 (t, *J* = 2.6 Hz, 3H), 1.65-1.50 (m, 4H), 1.22 (s, 3H), 1.14 (s, 3H), 1.11 (s, 3H) ppm.

^{13}C NMR (100 MHz, $CDCl_3$): δ = 163.9, 144.6, 141.5, 134.3, 131.8, 131.0, 130.3, 129.0, 127.5, 116.2, 110.5, 79.5, 77.0, 76.3, 74.9, 45.0, 43.2, 36.0, 34.2, 25.5, 24.9, 24.5, 23.3, 22.0, 17.1, 2.9 ppm.

IR (film): 3541, 3064, 2931, 2864, 1712, 1446, 1311, 1267, 1105 cm^{-1}.

HRMS (CI) **Calcd** for $C_{28}H_{37}O_3$: 421.6029. **Found**: 421.6021.

(1*S*,2*S*)-2-Hydroxy-1-[(*S*)-6-allyl-6-methylcyclohex-1-enyl]- 2-(1,1-dimethylallyl)hex-4-yn-1-yl benzoate (17b)

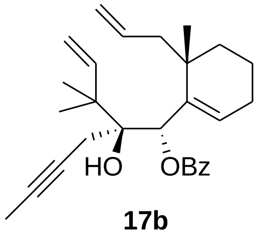

17b

Formula: $C_{28}H_{36}O_3$

MW: 420.6

$[\alpha]_D^{25}$: -33.2 (*c* 1.2, CH_2Cl_2).

^{1}H NMR (400 MHz, $CDCl_3$): δ = 8.00 (d, *J* = 7.7 Hz, 2H), 7.55 (t, *J* = 7.7 Hz, 1H), 7.44 (t, *J* = 7.7 Hz, 2H), 6.39 (t, *J* = 4.0 Hz, 1H), 6.09 (dd, *J* = 17.4, 10.6 Hz, 1H), 5.85 (s, 1H), 5.83-5.75 (m, 1H), 5.05-4.97 (m, 2H), 4.97 (d, *J* = 17.6 Hz, 1H), 4.91 (d, *J* = 10.7 Hz, 1H), 2.88 (s, 1H), 2.63 (dq, *J* = 17.0, 2.6 Hz, 1H), 2.56 (dq, *J* = 17.0, 2.6 Hz, 1H), 2.47-2.39 (m, 1H), 2.25-2.17 (m, 1H), 2.14-2.04 (m, 2H), 1.81 (t, *J* = 2.6 Hz, 3H), 1.66-1.51 (m, 4H), 1.24 (s, 3H), 1.17 (s, 3H), 1.12 (s, 3H) ppm.

^{13}C NMR (100 MHz, $CDCl_3$): δ = 164.0, 144.8, 141.6, 134.3, 131.9, 131.0, 130.2, 128.8, 127.4, 116.4, 110.9, 79.5, 77.2, 76.7, 75.0, 44.9, 43.4, 35.8, 34.8, 25.8, 24.9, 24.6, 23.2, 22.2, 17.0, 2.8 ppm.

IR (film): 3543, 2931, 2359, 1716, 1450, 1315, 1269, 1111 cm^{-1}.

HRMS (CI) **Calcd** for $C_{28}H_{37}O_3$: 421.6029. **Found**: 421.6021.

(1*S*,2*S*,8*S*,10*E*)-1-Hydroxy-8,12,14,14- tetramethyltricyclo[9.3.1.0{3,8}]pentadeca- 3,10,12-trien-2-yl benzoate (18b)

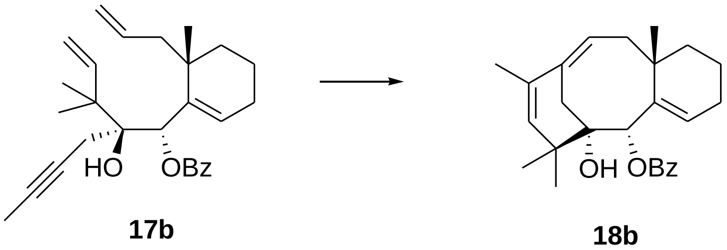

17b **18b**

Formula: $C_{26}H_{32}O_3$

MW: 392.5

To a thoroughly degassed solution of **17b** (14 mg, 33 µmol) in toluene (3 mL) was added second generation Grubbs' catalyst (1.4 mg, 1.6 µmol, 5 mol%). The mixture was stirred at reflux and monitored by TLC. After 1 h the mixture was cooled and the solvent was removed in vacuo and the crude mixture was purified by flash column chromatography and gave **18b** as a colourless oil (diethyl ether/petroleum ether: 8 mg, 57%).

$[\alpha]_D^{25}$: +14.8 (*c* 0.8, $CHCl_3$).

^{1}H NMR (400 MHz, $CDCl_3$): δ = 8.00 (d, J = 7.8 Hz, 2H), 7.56 (t, J = 7.8 Hz, 1H), 7.45 (t, J = 7.8 Hz, 2H), 6.12 (m, 1H), 5.77 (t, J = 8.0 Hz, 1H), 5.47 (s, 1H), 4.97 (m, 1H), 2.84 (dd, J = 12.7, 8.4 Hz, 1H), 2.66 (d, J = 11.4 Hz, 1H), 2.33 (s, 1H), 2.29 (d, J = 11.6 Hz, 1H), 2.18 (dt, J = 18.4, 5.3 Hz, 1H), 2.05-1.93 (m, 1H), 1.80 (s, 3H), 1.71 (dd, J = 12.9, 7.5 Hz, 1H), 1.63-1.57 (m, 2H), 1.46-1.35 (m, 2H), 1.19 (s, 3H), 1.13 (s, 3H), 0.89 (s, 3H) ppm.

^{13}C NMR (126 MHz, $CDCl_3$): δ = 163.4, 143.1, 138.0, 132.8, 132.0, 130.8, 130.0, 128.5, 127.9, 127.6, 121.5, 78.1, 72.0, 42.2, 42.0, 40.8, 38.3, 33.5, 26.5, 25.8, 25.0, 23.6, 17.5, 16.9 ppm.

IR (film): 3542, 2927, 1718, 1451, 1320, 1266, 1113, 1068 cm^{-1}.

HRMS (EI) **Calcd** for $C_{26}H_{32}O_3$: 392.2351. **Found**: 392.2348.

(1*S*,2*S*)-1-[(*S*)-6-allyl-6-methylcyclohex-1-enyl]-2-(1,1-dimethylallyl)hex-4-yn-1,2-diol (19)

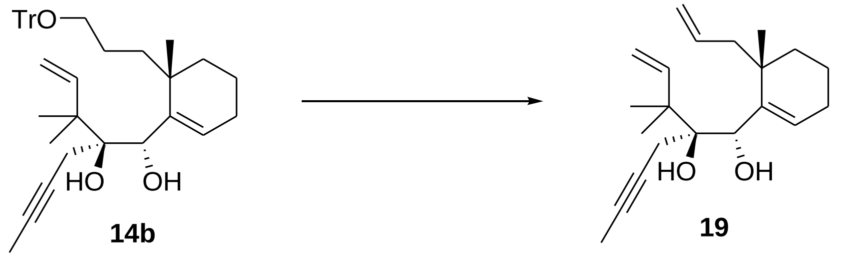

Formula: $C_{21}H_{32}O_2$

MW: 316.5

Diol **14b** (600 mg, 1.04 mmol) was dissolved in undistilled MeOH (20 mL) at room temperature. Amberlyst H-15 (230 mg) was then added in one portion and the reaction mixture was stirred overnight at room temperature. The resin was then filtered off and washed thoroughly with ethyl acetate and the solvents were removed under reduced pressure. Purification of crude residue by

flash chromatography (petroleum ether/Et_2O: 75/25) afforded the desired triol (340 mg, 98%) as a colorless oil.

To a solution of the previous triol (220 mg, 0.66 mmol) in THF (15 mL) were added imidazole (134 mg, 1.97 mmol, 3.0 equiv), triphenyl phosphine (517 mg, 1.97 mmol, 3.0 equiv) and iodine (251 mg, 0.99 mmol, 1.5 equiv). After being stirred at room temperature for 30 min, the reaction mixture was quenched with saturated aqueous Na_2SO_3. The resulting mixture was diluted with ethyl acetate, washed with water and brine, dried over $MgSO_4$, filtered and concentrated in vacuo. Purification of crude residue by flash chromatography over silica gel (petroleum ether/Et_2O: 88/12) gave the desired iodide (240 mg, 82%) as a colorless oil.

To a solution of the previous iodide (210 mg, 0.47 mmol) in DMF (8 mL) at 0°C was added NaH (60 % in oil, 61 mg, 1.53 mmol, 3.25 equiv) in DMF (4 mL). After being stirred at room temperature overnight, the reaction mixture was quenched with water at 0°C. The resulting reaction mixture was diluted with ethyl acetate, washed with water and brine, dried over $MgSO_4$, filtered and concentrated in vacuo. Purification of the crude residue by flash chromatography over silica gel (petroleum ether/Et_2O: 90/10) gave diene **19** (125 mg, 84%) as a colorless oil.

^{1}H NMR (400 MHz, $CDCl_3$): δ = 6.30-6.23 (m, 2H), 5.85-5.74 (m, 1H), 5.10-5.00 (m, 4H), 4.29 (d, J = 4.4 Hz, 1H), 3.08 (s, 1H), 2.52-2.49 (m, 2H), 2.14 (d, J = 7.2 Hz, 2H), 2.16-2.00 (m, 3H), 1.76 (t, J = 2.4 Hz, 3H), 1.66-1.60 (m, 1H), 1.58-1.49 (m, 2H), 1.34-1.27 (m, 1H), 1.24 (s, 3H), 1.19 (s, 3H), 1.07 (s, 3H) ppm.

^{13}C NMR (100 MHz, $CDCl_3$): δ = 146.8, 146.5, 134.9, 128.0, 117.2, 112.5, 79.4, 77.5, 76.5, 69.6, 46.4, 44.1, 37.0, 35.1, 26.1, 25.8, 25.3, 23.8, 23.3, 18.3, 3.6 ppm.

IR (CH_2Cl_2): 3588, 3526, 3038, 3042, 2986, 2955, 1464, 1415, 1380, 1172, 1112, 1011 cm^{-1}.

(5*S*)-5-{(*S*)-[(*S*)-6-allyl-6-methylcyclohex-1-enyl]hydroxymethyl}-tetrahydro-2-methyl-5-(2-methylbut-3-en-2-yl)furan-2-ol (20)

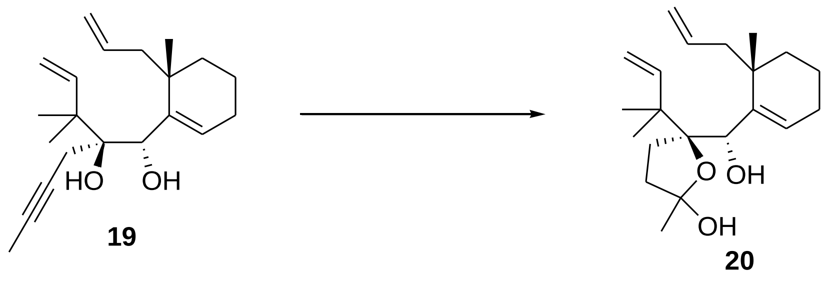

Formula: $C_{21}H_{34}O_3$

MW: 334.5

To a solution of diene **19** (125 mg, 0.390 mmol) in THF (6 mL) and water (4 drops) at room temperature was added Ph_3AuNTf_2 (14.6 mg, 0.019 mmol, 5 mol%). The resulting reaction mixture was stirred for 30 min at room temperature and water (3 mL) was added. The mixture was diluted with diethyl ether, washed with brine, dried over $MgSO_4$, filtered and concentrated in vacuo. Purification of the crude residue by flash chromatography over silica gel (petroleum ether/Et_2O: 97/3) gave hemiketal **20** (108 mg, 80%) as a colorless oil.

^{1}H NMR (400 MHz, $CDCl_3$): δ = 6.30 (dd, J = 17.0, 11.2 Hz, 1H), 5.94 (t, J = 3.8 Hz, 1H), 5.78-5.68 (m, 1H), 5.08-4.95 (m, 4H), 4.35 (s, 1H), 2.33-2.13 (m, 4H), 2.08-2.03 (m, 2H), 1.94 (dd, J = 14.8, 8.4 Hz, 1H), 1.82 (dd, J = 14.8, 8.4 Hz, 1H), 1.67 (dt, J = 17.6, 5.0 Hz, 1H), 1.73-1.55 (m, 4H), 1.53 (s, 3H), 1.37-1.20 (m, 1H), 1.15 (s, 3H), 1.13 (s, 3H), 1.06 (s, 3H) ppm.

^{13}C NMR (100.6 MHz, $CDCl_3$): δ = 145.1, 141.9, 135.3, 127.0, 117.0, 111.9, 106.9, 94.3, 79.2, 77.2, 44.1, 39.3, 37.6, 35.5, 26.2, 26.1, 25.3, 24.3, 23.3, 19.5, 17.9 ppm.

(5*S*,8*Z*,10a*S*)-1,2,3,5,6,7,10,10a-octahydro-5-hydroxyl-7,7,10a-trimethylbenzyl-2'-hydroxyl-2'-methylspiro[[8]annulene-6,5'-tetrahydrofuran] (21)

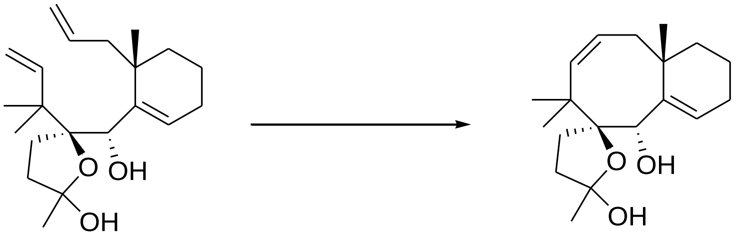

20 → **21**

Formula: $C_{19}H_{30}O_3$

MW: 306.4

Diene **20** (66 mg, 0.197 mmol) was dissolved in dichloromethane (8 mL). The mixture was thoroughly degassed three times while stirring, second-generation Grubbs catalyst (8.3 mg, 0.0098 mmol, 5 mol%) was added and the reaction mixture was refluxed for 1.5 h. After cooling, the solvent was evaporated under reduced pressure. Purification of the crude product by flash chromatography over silica gel (petroleum ether/Et_2O: 96/4) afforded olefin **21** (59 mg, 98%) as a white solid.

mp: 93°C.

^{1}H NMR (400 MHz, $CDCl_3$): δ = 5.88 (dd, J = 5.4, 2.4 Hz, 1H), 5.58 (dt, J = 11.3, 7.2 Hz, 1H), 5.28 (dd, J = 11.3, 1.2 Hz, 1H), 4.65 (s, 1H), 2.66 (dd, J = 13.4, 11.3 Hz, 1H), 2.19-2.12 (m, 1H), 2.08-1.91 (m, 3H), 1.78-1.60 (m, 6H), 1.65 (dd, J = 13.4, 11.3 Hz, 1H), 1.55 (s, 3H), 1.49 (s, 3H), 1.25 (s, 3H), 1.26 (s, 3H) ppm.

^{13}C NMR (100.6 MHz, $CDCl_3$): δ = 140.6, 138.3, 130.2, 124.6, 107.0, 94.6, 79.9, 38.8, 38.4, 38.3, 36.7, 35.3, 28.9, 28.6, 25.1, 23.0, 22.7, 19.1, 18.5 ppm.

HRMS (EI) **Calcd** for $C_{19}H_{28}O_2$: 288.2089. **Found:** 288.2084.

2-(2-Methylpent-3-yn-2-yl)-1,3-dithiane (39)

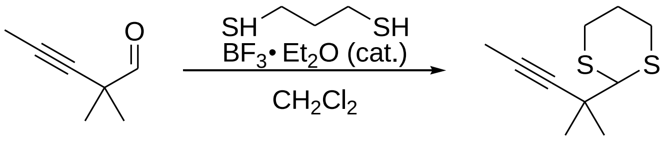

39

Formula: $C_{10}H_{16}S_2$

MW: 200.4

To a solution of aldehyde **29** (770 mg, 7.00 mmol) in dichloromethane (40 mL) was added 1,3-propanedithiol (1.0 mL, 10.5 mmol, 1.5 equiv) and boron trifluoride etherate (180 µL, 1.40 mmol, 0.2 equiv) dropwise at 0°C. The reaction mixture was stirred at this temperature for 16 h. A 5% aqueous solution of NaOH (40 mL) was added and the aqueous layer was extracted with dichloromethane (3x40 mL), and the combined organic extracts were washed with brine (100 mL), dried over anhydrous Na_2SO_4, filtered and concentrated in vacuo. The crude mixture was purified by flash chromatography (petroleum ether/Et_2O: 95/5) to afford the title dithiane **39** (1.05 g, 5.25 mmol, 75%) as a yellow oil.

^{1}H NMR (400 MHz, $CDCl_3$): δ = 4.08 (s, 1H), 2.93-2.90 (m, 4H), 2.10-2.07 (m, 1H), 1.87-1.82 (m, 1H), 1.84 (s, 3H), 1.40 (s, 6H) ppm.

^{13}C NMR (126 MHz, $CDCl_3$): δ = 83.7, 77.9, 59.6, 36.4, 31.1, 27.9, 25.8, 3.7 ppm.

HRMS (EI) **Calcd** for $C_{10}H_{16}S_2$: 200.0693. **Found** 200.0692.

2-(3-Methylbut-2-enyl)-2-(2-methylpent-3-yn-2-yl)-1,3-dithiane (40)

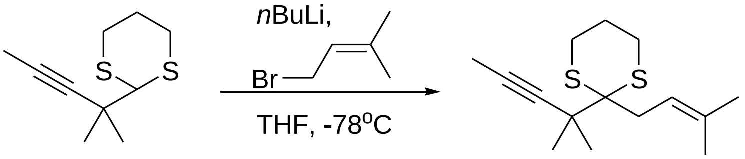

40

Formula: $C_{15}H_{24}S_2$

MW: 268.5

To a solution of dithiane **39** (2.60 g, 13.0 mmol) in THF (150 mL) was added at -78°C *n*-BuLi (6.8 mL, 2.5 M in hexanes, 17 mmol, 1.3 equiv). The reaction mixture was stirred 30 min at this temperature and 30 min at 0°C and then cooled down to -78°C and prenyl bromide (1.7 mL, 14 mmol, 1.1 equiv) was added neat. The mixture was stirred and allowed to reach room temperature over a period of 2.5 h. Water (100 mL) and dichloromethane (100mL) were added, the aqueous layer was extracted with dichloromethane (3x100 mL), and the combined organic extracts were washed with a 7% NaOH aqueous solution (250 mL), washed with brine (250 mL), dried over anhydrous Na_2SO_4, filtered and concentrated *in vacuo*. The crude mixture was purified by flash chromatography (petroleum ether/Et_2O: 95/5) to afford the title dithiane **40** (3.38 g, 12.6 mmol, 97%) as a yellow oil.

^{1}H NMR (500 MHz, $CDCl_3$): δ = 5.57-5.54 (m, 1H), 2.92-2.82 (m, 6H), 2.03-1.94 (m, 1H), 1.90-1.79 (m, 1H), 1.84 (s, 3H) 1.75 (d, J = 1.2 Hz, 3H), 1.70 (s, 3H), 1.45 (s, 6H) ppm.

^{13}C NMR (126 MHz, $CDCl_3$): δ = 132.0, 122.2, 78.6, 77.2, 62.2, 42.7, 36.9, 27.0, 26.6, 26.1, 24.1, 18.3, 3.7 ppm.

HRMS (CI) **Calcd** for $C_{15}H_{25}S_2$: 269.1398. **Found** 269.1394.

2,6,6-Trimethylnon-2-en-7-yn-5-one (38)

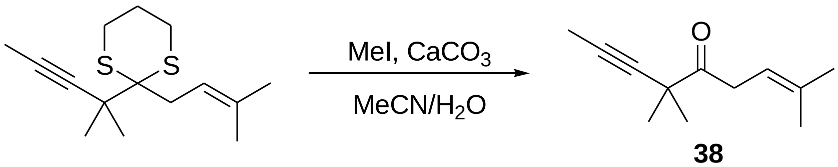

38

Formula: $C_{12}H_{18}O$

MW: 178.3

To a solution of dithiane **40** (3.38 g, 12.6 mmol) in a 1:1 mixutre of MeCN/H_2O (250 mL) was added $CaCO_3$ (14.0 g, 126 mmol, 10 equiv) followed by MeI (3.1 mL, 50 mmol, 4 equiv). The resulting mixture was allowed to stir at 40°C for 48 h. Then a saturated aqueous solution of $NaHCO_3$ (150 mL) was added and the aqueous layer was extracted with Et_2O (3x150 mL), then the combined organic extracts were washed with brine (250 mL), dried over anhydrous $MgSO_4$, filtered and concentrated *in vacuo*. The crude mixture was purified by flash chromatography (petroleum ether/Et_2O: 97/3) to afford the title ketone **38** (1.86 g, 10.5 mmol, 83%) as a colourless oil.

^{1}H NMR (400 MHz, $CDCl_3$): δ = 5.32 (m, 1H), 3.48 (d, *J* = 5.6 Hz, 2H), 1.83 (s, 3H), 1.74 (s, 3H), 1.63 (s, 3H), 1.32 (s, 6H) ppm.

^{13}C NMR (100 MHz, $CDCl_3$): δ = 209.2, 134.9, 116.7, 82.3, 78.9, 43.6, 37.3, 26.4, 25.7, 18.1, 3.6 ppm.

IR (ν, cm^{-1}): 2981, 2923, 1716, 1450, 1380, 1264, 1113, 1080, 1040 cm^{-1}.

HRMS (CI) **Calcd** for $C_{12}H_{19}O$: 179.1436. **Found** 179.1433.

The experimental procedures for compounds **24**, **26-31**, **32a,b-35a,b**, **36**, **41**, **42a,b-43a,b**, **44** and **45** are reported in the Supporting Information of the preliminary account of this work: A. Letort, R. Aouzal, C. Ma, D.-L. Long, J. Prunet, *Org. Lett*. **2014**, *16*, 3300-3303.

3. X-ray Data of 16b and 33a:

CCDC 1427199

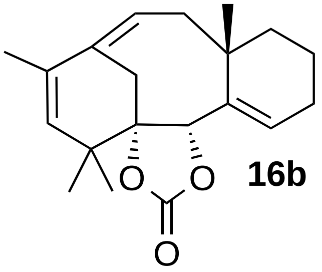

16b

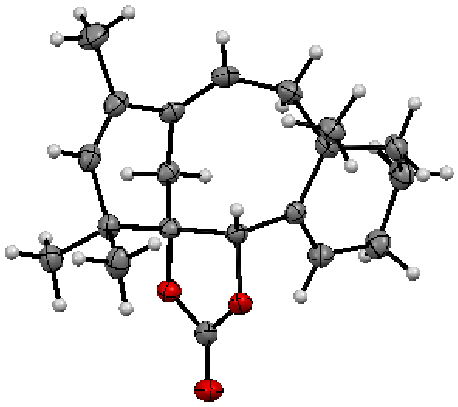

Compound	**16b**
Molecular formula	$C_{20}H_{26}O_3$
Molecular weight	314.41
Crystal habit	colorless plate
Crystal dimensions(mm)	0.400x0.240x0.050
Crystal system	orthorhombic
Space group	P 2_1 2_1 2_1
a(Å)	7.5880(2)
b(Å)	9.4732(5)
c(Å)	23.3607(12)
α(°)	90
β(°)	90
γ(°)	90
V(Å^3)	1679.23(13)
Z	4
d(g-cm^{-3})	1.244
F(000)	680
μ(cm^{-1})	0.082
Absorption corrections	multi-scan;
Diffractometer	Kappa APEX II
X-ray source	MoKα
λ(Å)	0.71069
Monochromator	graphite
T (K)	150.0(1)
Scan mode	phi and omega scans
Maximum θ	27.463
HKL ranges	-9 8 ; -12 8 ; -30 27
Reflections measured	9057
Unique data	3804
Rint	0.0263
Reflections used	2961

Criterion	I > 2σI)
Refinement type	Fsqd
Hydrogen atoms	constr
Parameters refined	214
Reflections / parameter	13
wR2	0.0784
R1	0.0371
Flack's parameter	0.6(12)
Weights a, b	0.0365 ; 0.0000
GoF	0.993
difference peak / hole (e Å^{-3})	0.176(0.036) / -0.159(0.036)

CCDC 1405009

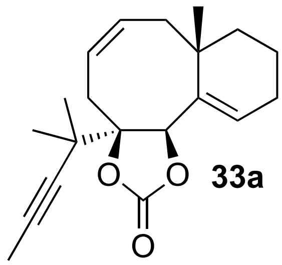

33a

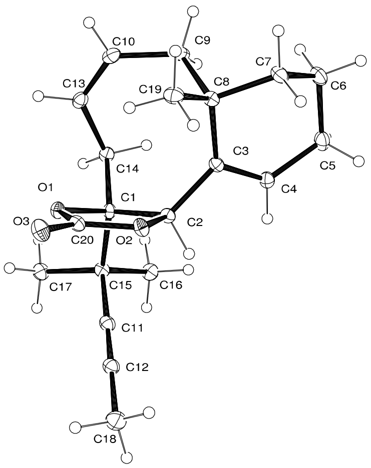

Compound name	**33a**
Empirical formula	C20 H26 O3
Formula weight	314.41
Temperature	100 K
Wavelength	0.71073
Crystal system	Orthorhombic
Space group	P 21 21 21
Unit cell dimension	a = 7.5528(2) Å
	b = 11.6745(3) Å
	c = 19.2152(6) Å
	α = 90 °
	β = 90 °
	γ = 90 °
Volume	1694.30(8) Å^3
Z	4

Density (calculated)	1.233 g/cm^3
Radiation type	MoK\a
Absorption coefficient	0.081 μ/mm
F(000)	680.0
Theta range for data collection	2.336 - 27.506 °
Index ranges	−9<=h<=+9; −15<=k<=+15; −24<=l<=+24
R*int*	0.043
Absorption correction type	Gaussian
Refinement method	Full-matrix least-squares on F^2
Final R indices [I>2sigma(I)]	R1 = 0.0316, wR2 = 0.0765
R indices (all data)	R1 = 0.0398, wR2 = 0.0975
Largest diff. peak and hole	0.23 and -0.15 e.Å^{-3}

The X-ray data of compound **45** are reported in the Supporting Information of the preliminary account of this work:
A. Letort, R. Aouzal, C. Ma, D.-L. Long, J. Prunet, *Org. Lett*. **2014**, *16*, 3300-3303.

4. NMR Spectra of New Compounds

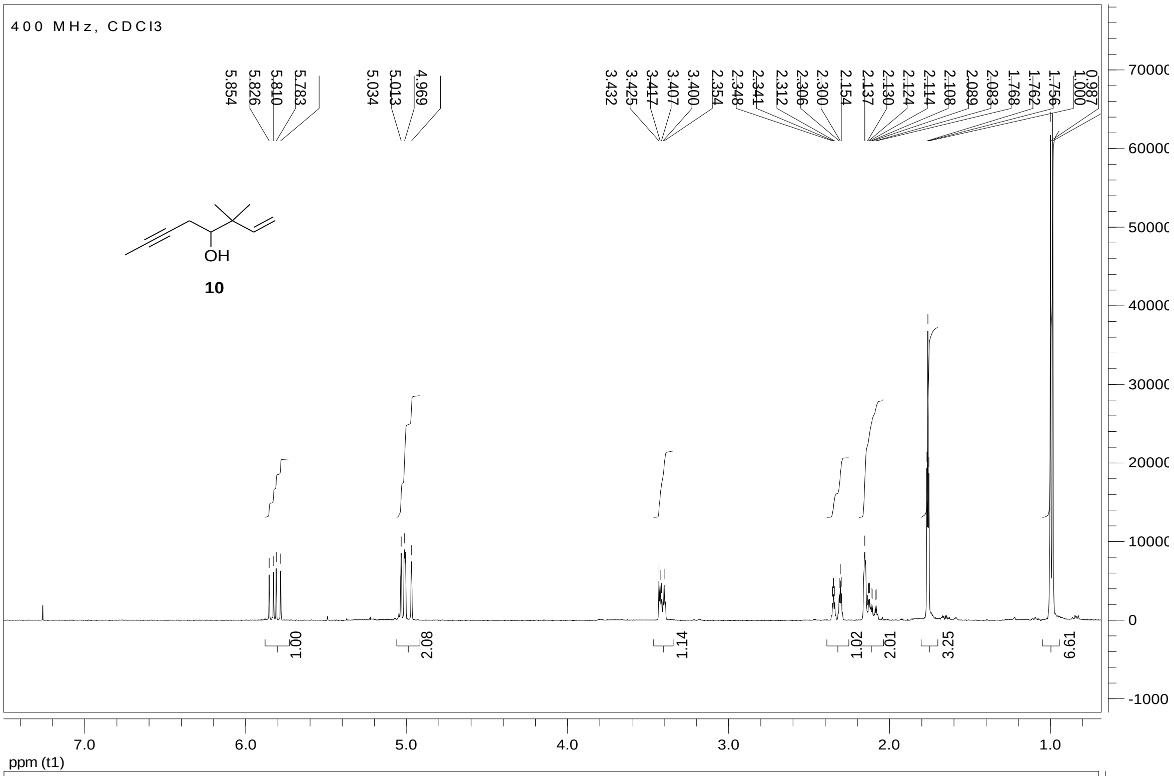

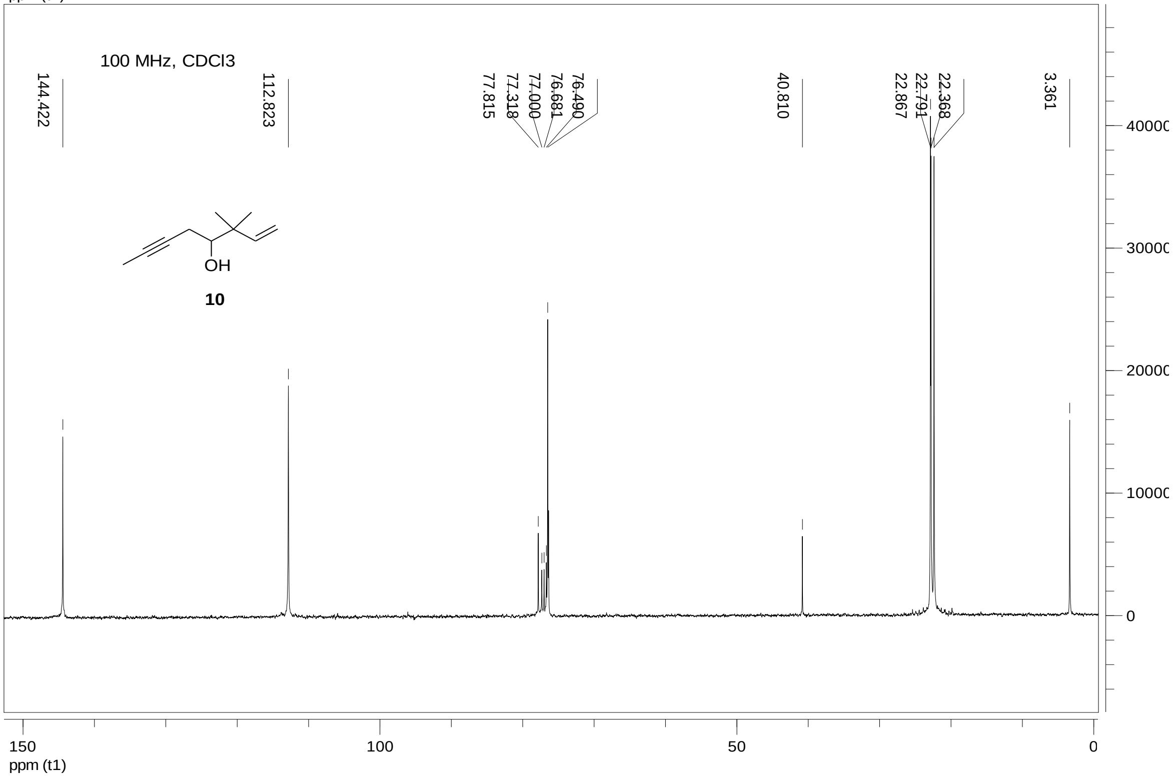

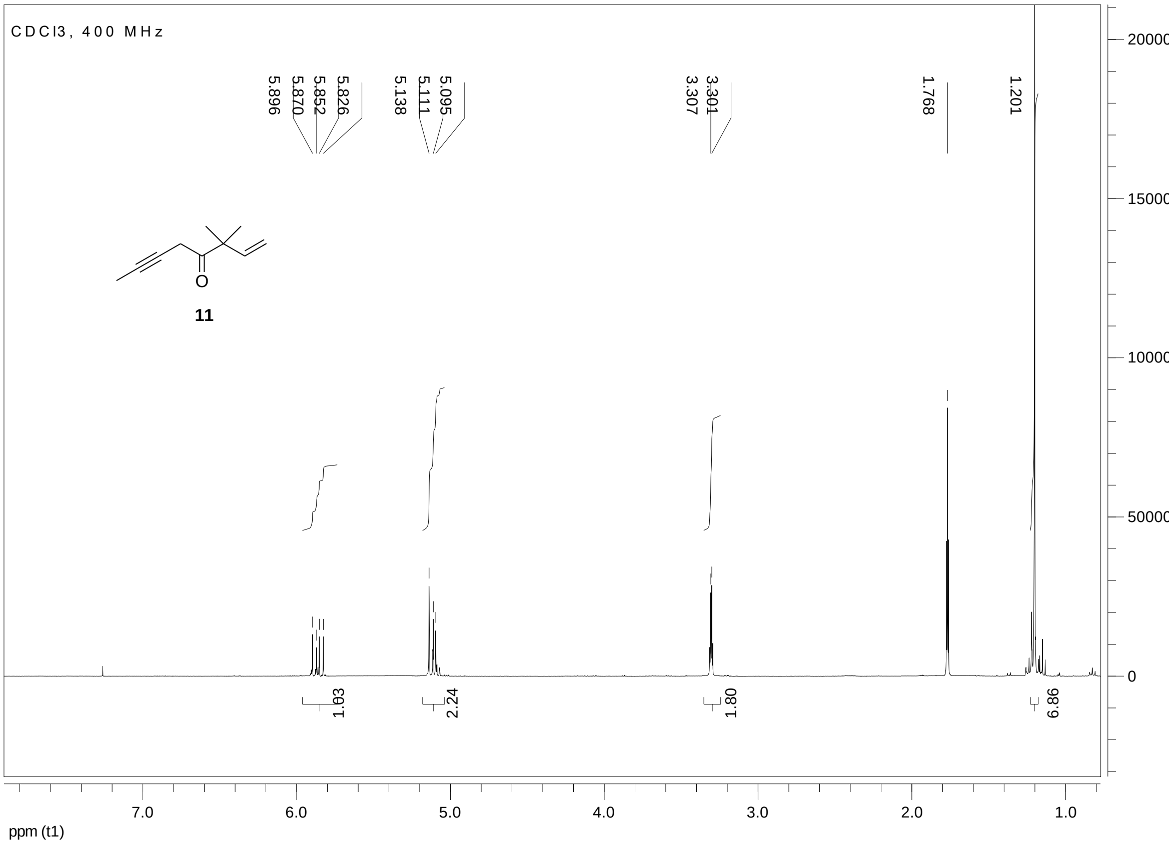
CDCl3, 400 MHz
5.896
5.870
5.852
5.826
5.138
5.111
5.095
3.301
3.307
1.768
1.201
O
11
1.03
2.24
1.80
6.86
7.0
6.0
5.0
4.0
3.0
2.0
1.0
ppm (t1)

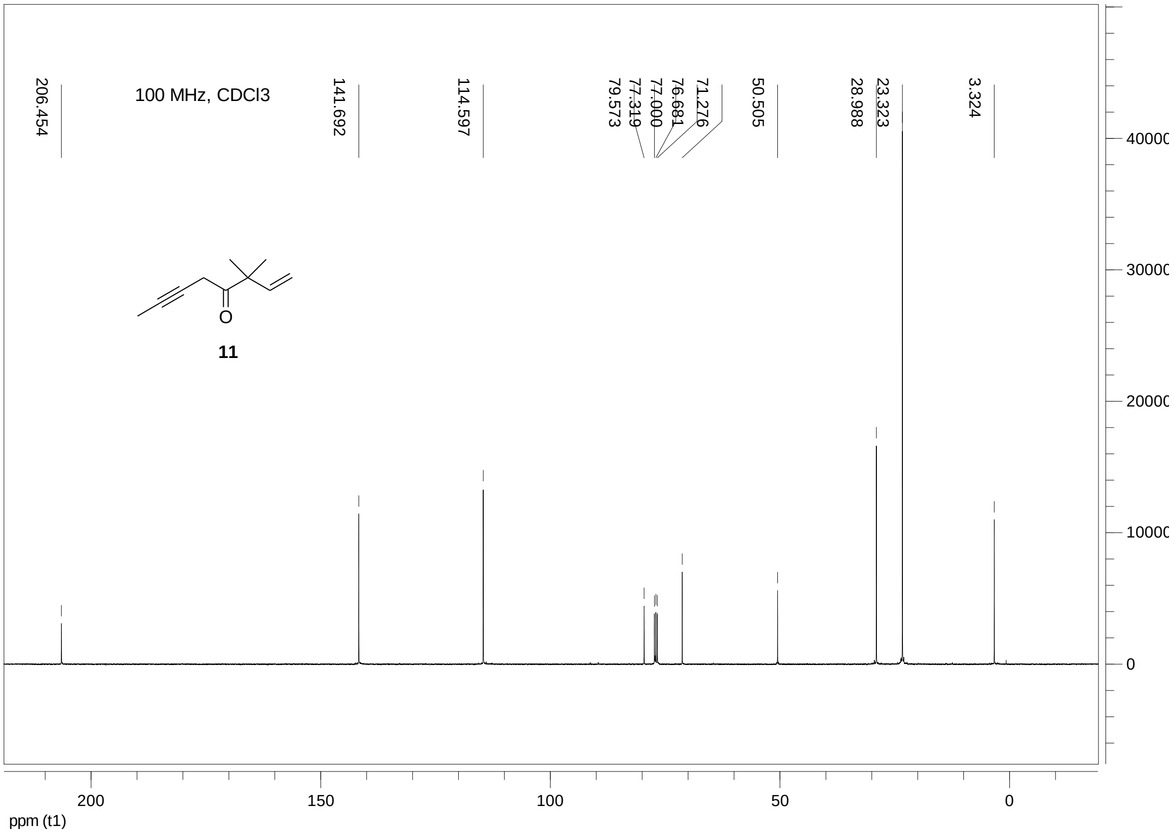
206.454
100 MHz, CDCl3
141.692
114.597
79.573
77.319
77.000
76.681
71.276
50.505
28.988
23.323
3.324
O
11
200
150
100
50
0
ppm (t1)

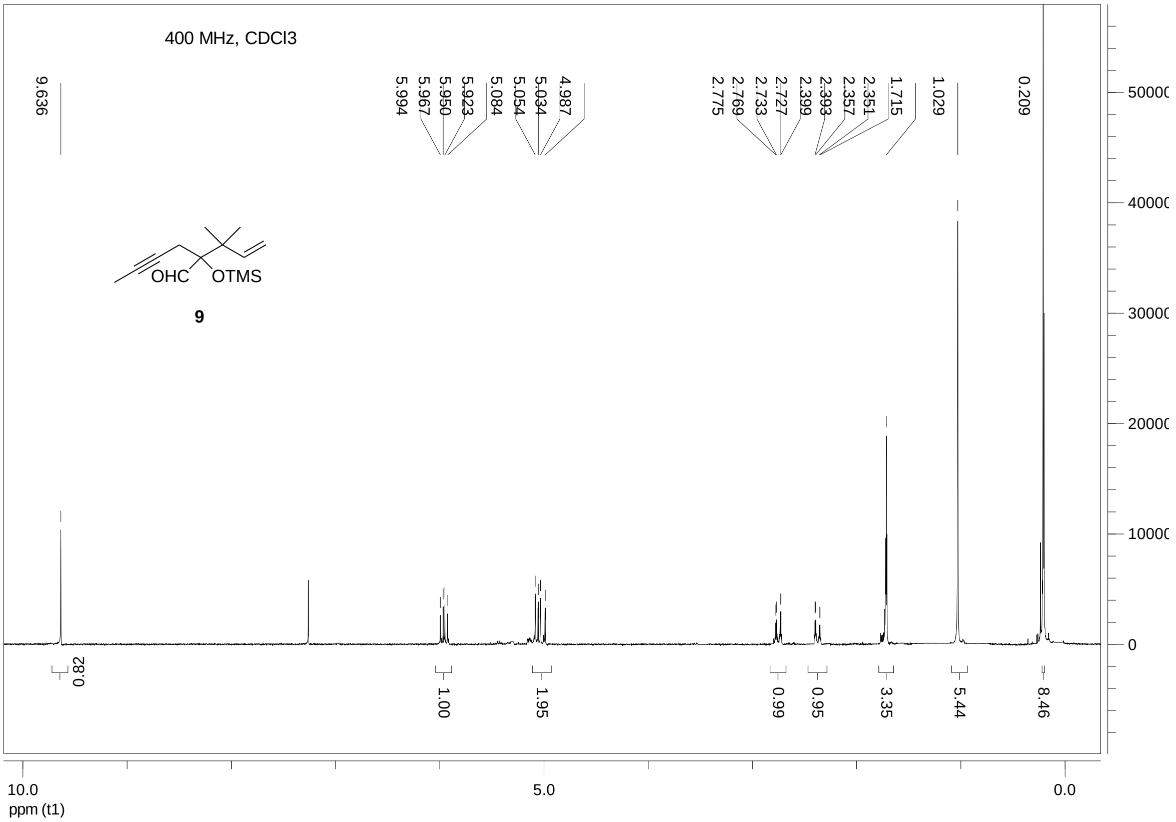
400 MHz, CDCl3
9.636
5.994 5.967 5.950 5.923 5.084 5.054 5.034 4.987
2.775 2.769 2.733 2.727 2.399 2.393 2.357 2.351 1.715
1.029
0.209
OHC OTMS
9
0.82 1.00 1.95 0.99 0.95 3.35 5.44 8.46
10.0
5.0
0.0
ppm (t1)

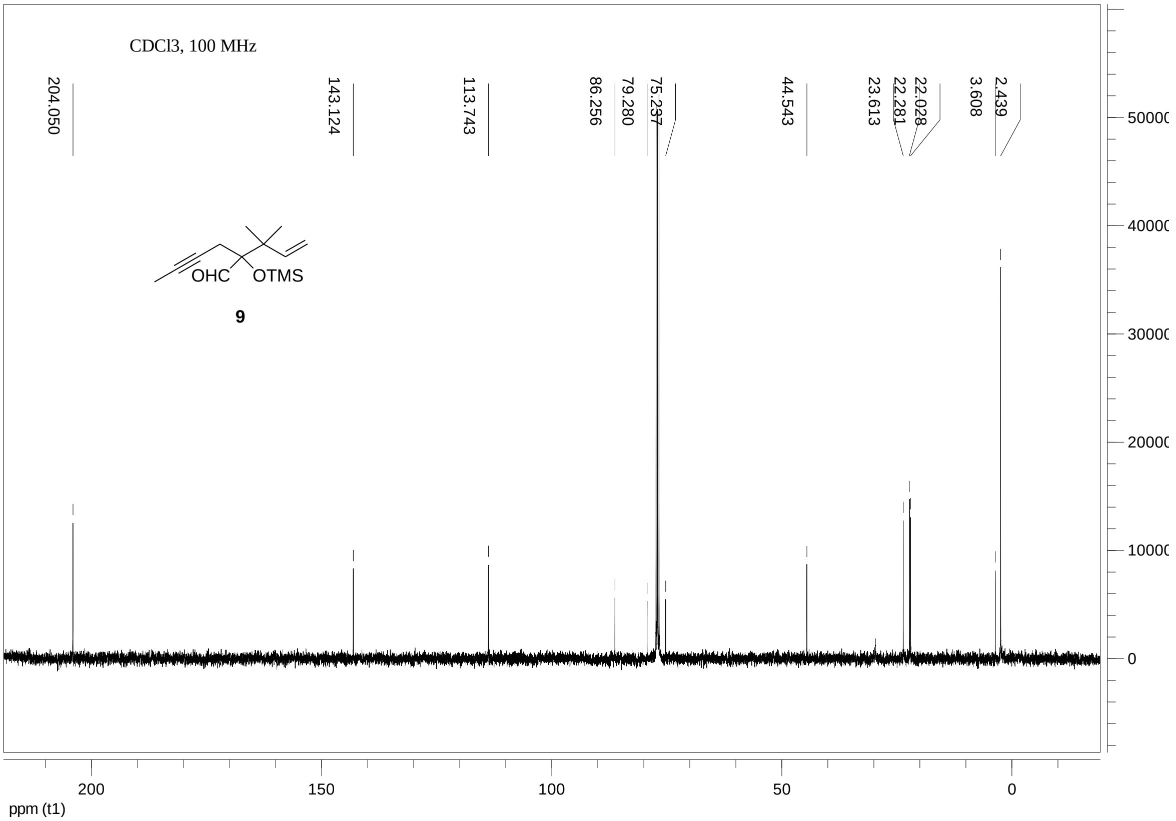
CDCl3, 100 MHz
204.050
143.124
113.743
86.256 79.280 75.237
44.543
23.613 22.281 22.028
3.608 2.439
OHC OTMS
9
200
150
100
50
0
ppm (t1)

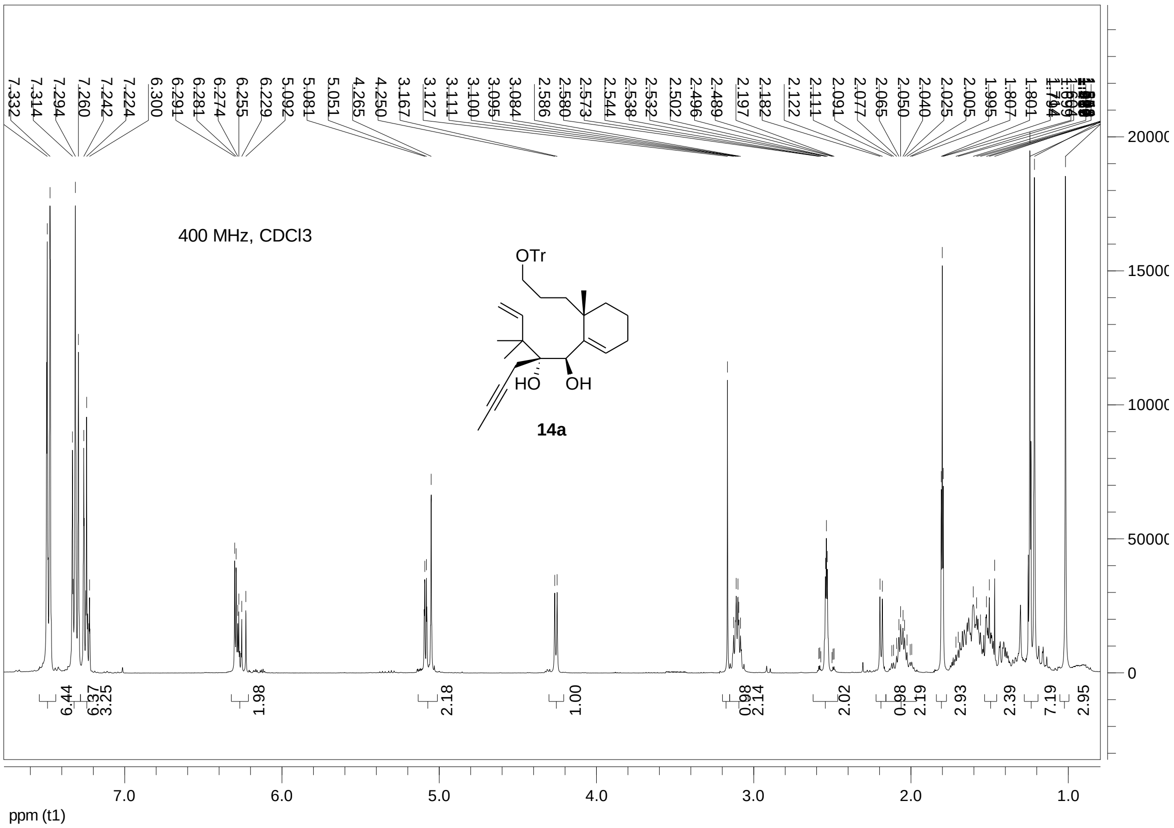

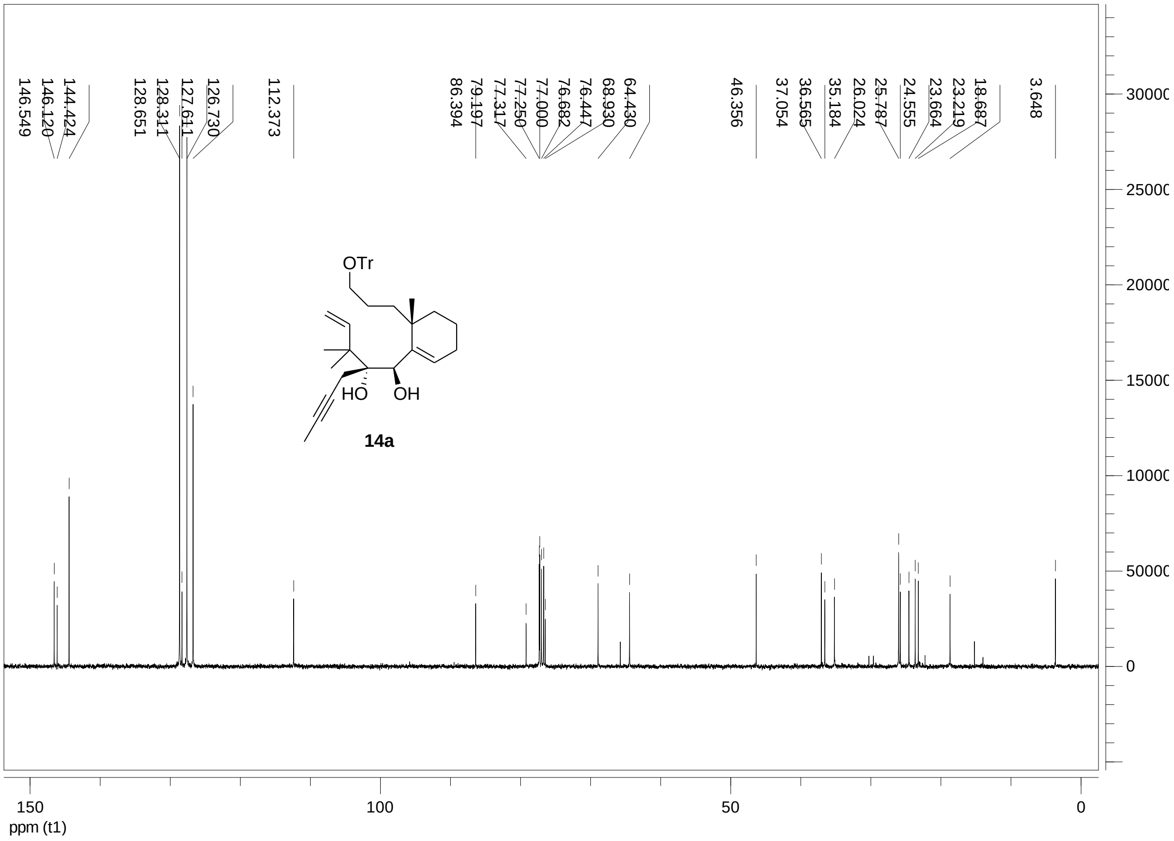

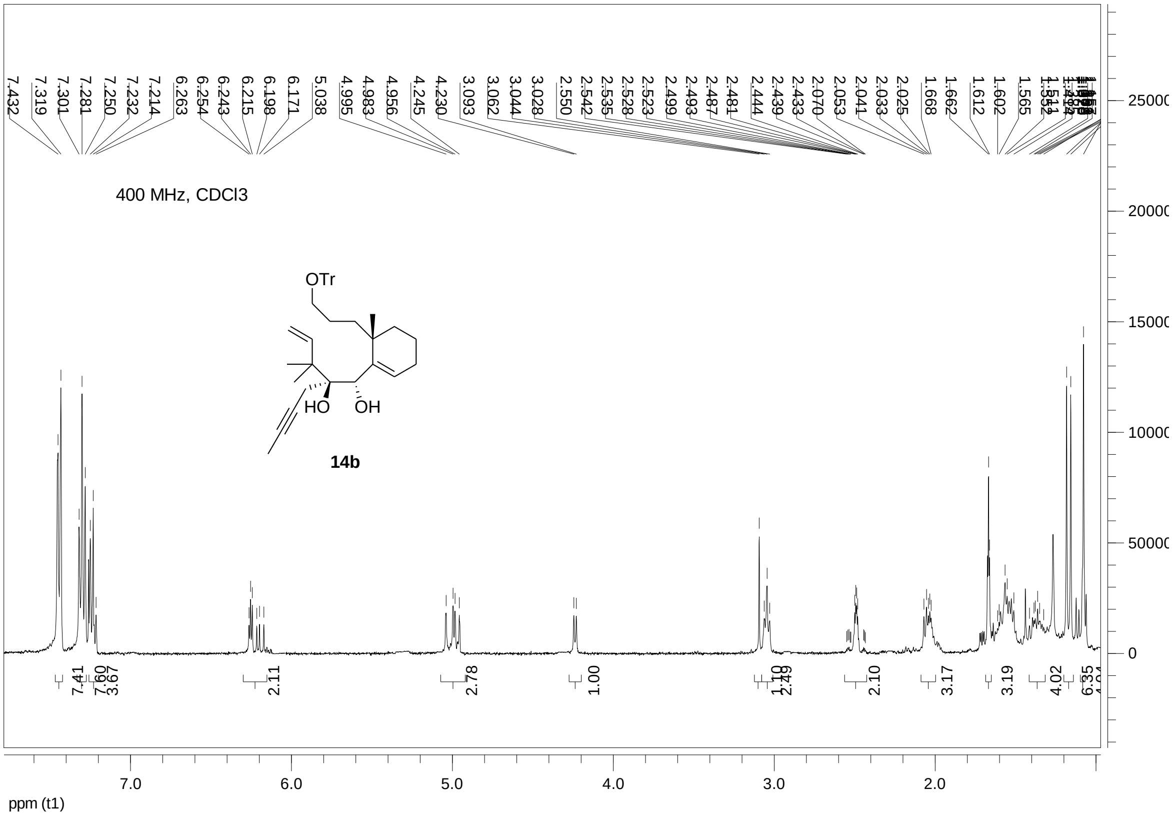
400 MHz, CDCl3
OTr
HO
OH
14b
7.0
6.0
5.0
4.0
3.0
2.0
ppm (t1)

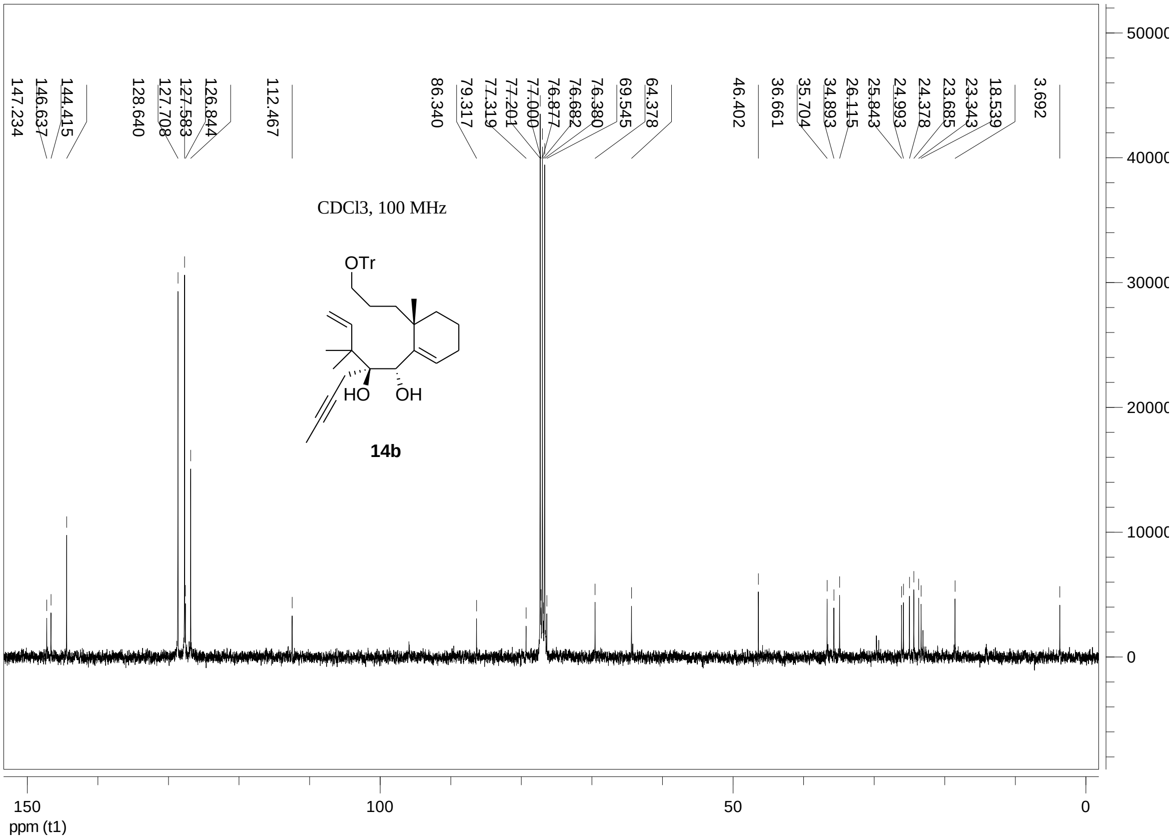
147.234
146.637
144.415
128.640
127.708
127.583
126.844
112.467
86.340
79.317
77.319
77.201
77.000
76.877
76.682
76.380
69.545
64.378
46.402
36.661
35.704
34.893
26.115
25.843
24.993
24.378
23.685
23.343
18.539
3.692
CDCl3, 100 MHz
OTr
HO
OH
14b
150
100
50
0
ppm (t1)

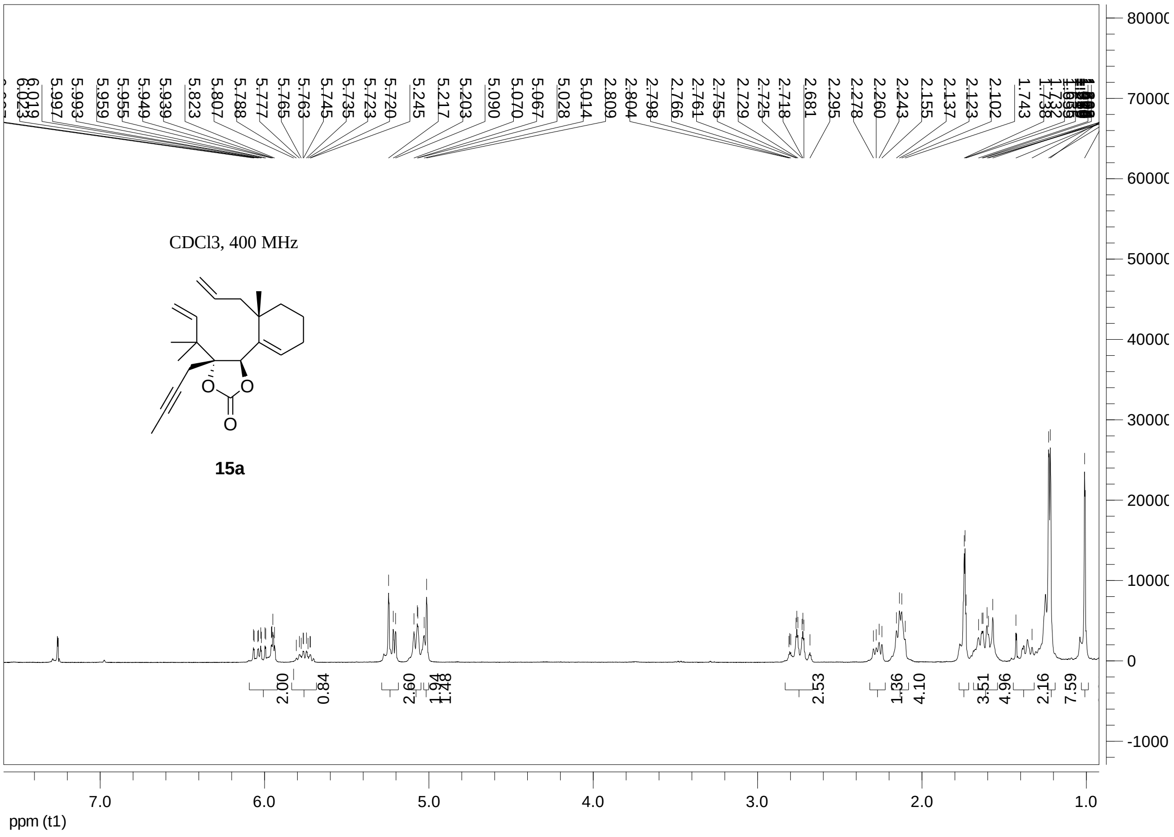
CDCl3, 400 MHz
15a
ppm (t1)

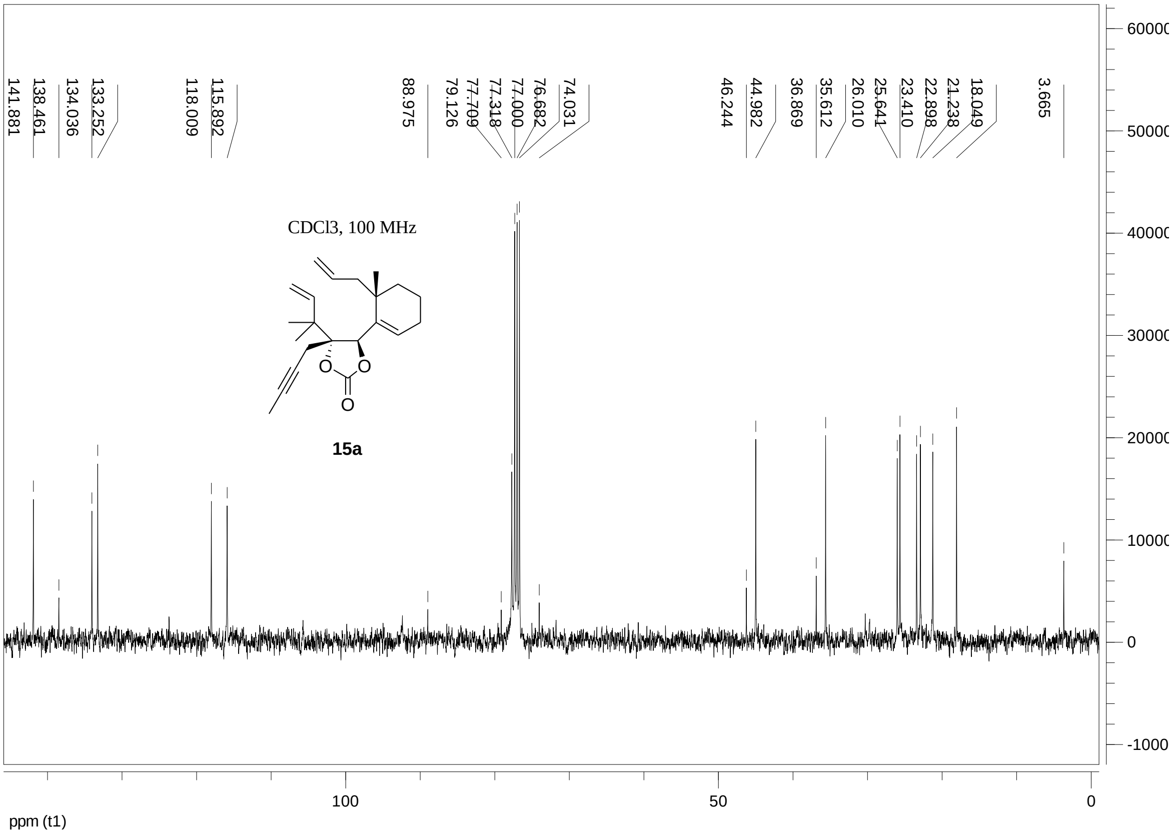
CDCl3, 100 MHz
15a
141.881
138.461
134.036
133.252
118.009
115.892
88.975
79.126
77.709
77.318
77.000
76.682
74.031
46.244
44.982
36.869
35.612
26.010
25.641
23.410
22.898
21.238
18.049
3.665
ppm (t1)

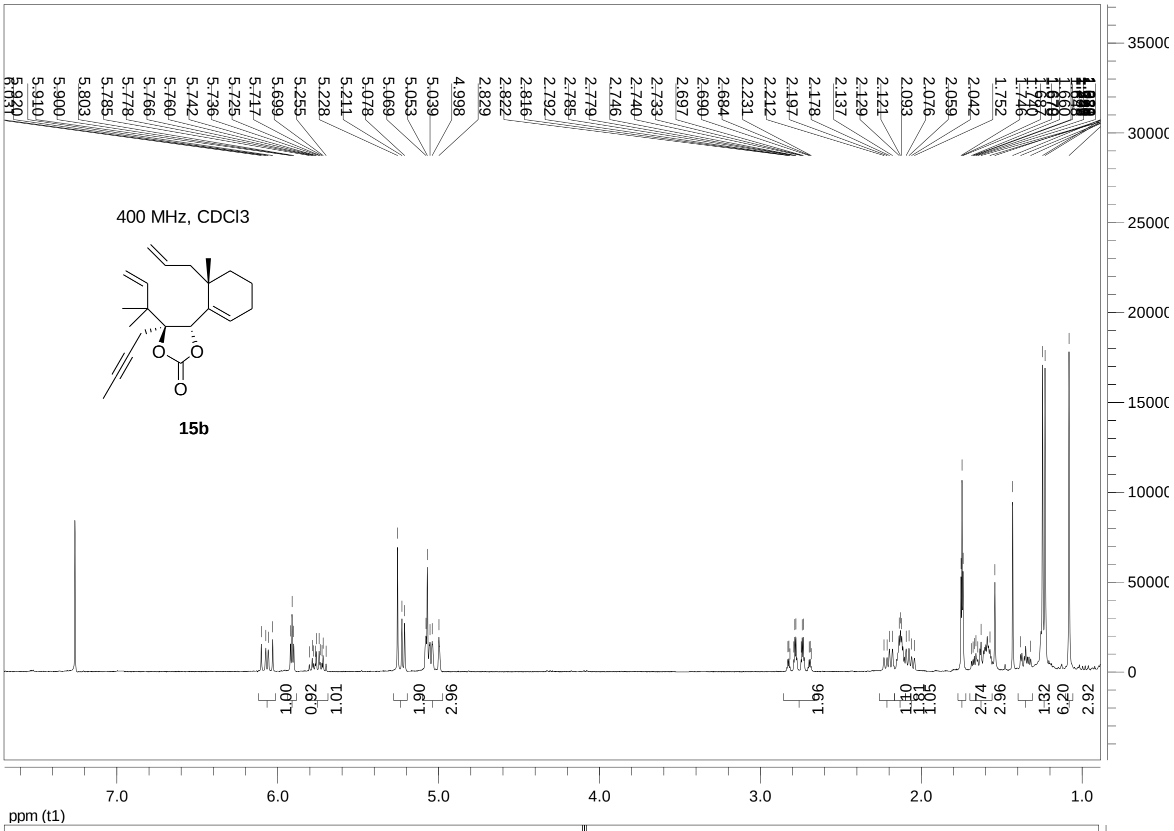
400 MHz, CDCl3
15b
6.031
5.920
5.910
5.900
5.803
5.785
5.778
5.766
5.760
5.742
5.736
5.725
5.717
5.699
5.255
5.228
5.211
5.078
5.069
5.053
5.039
4.998
2.829
2.822
2.816
2.792
2.785
2.779
2.746
2.740
2.733
2.697
2.690
2.684
2.231
2.212
2.197
2.178
2.137
2.129
2.121
2.093
2.076
2.059
2.042
1.752
1.746
1.740
1.679
1.660
1.00
0.92
1.01
1.90
2.96
1.96
1.10
1.81
1.05
2.74
2.96
1.32
6.20
2.92
7.0
6.0
5.0
4.0
3.0
2.0
1.0
ppm (t1)
35000
30000
25000
20000
15000
10000
50000
0

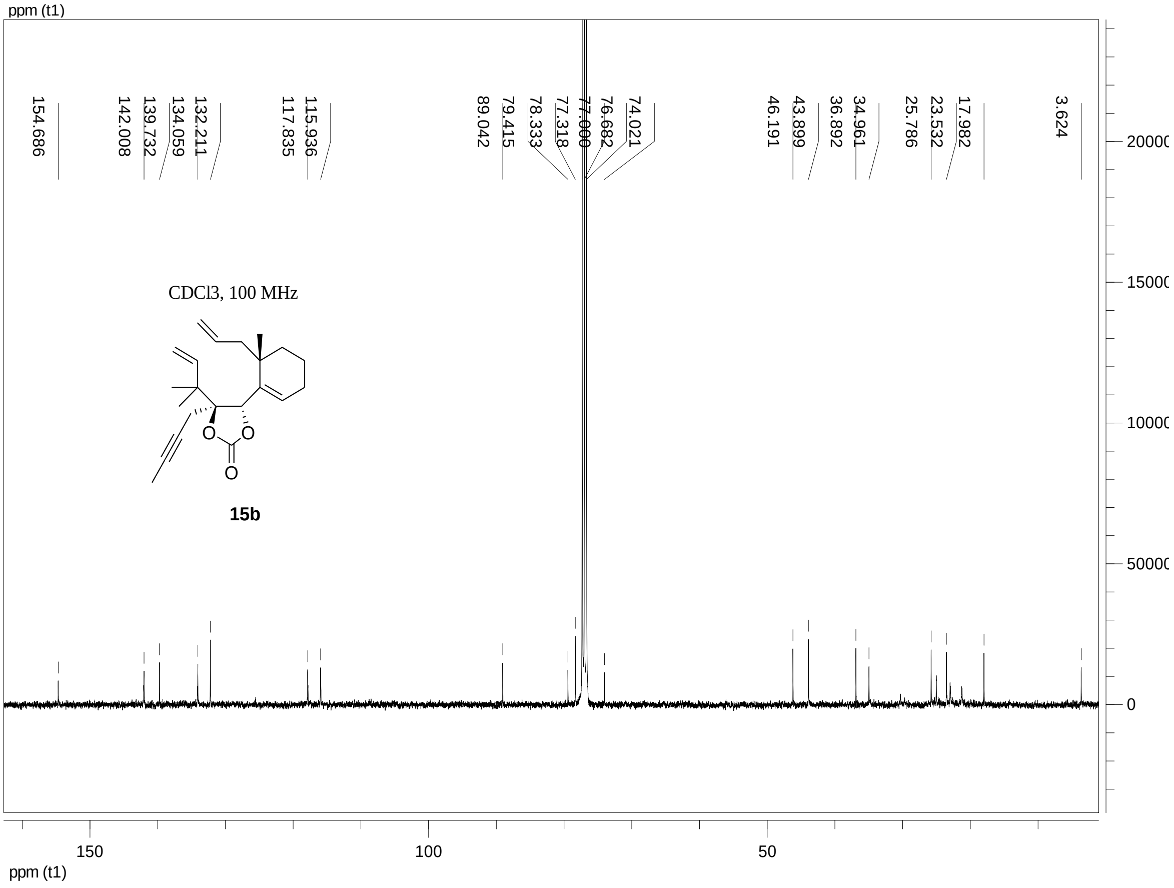
CDCl3, 100 MHz
15b
154.686
142.008
139.732
134.059
132.211
117.835
115.936
89.042
79.415
78.333
77.318
77.000
76.682
74.021
46.191
43.899
36.892
34.961
25.786
23.532
17.982
3.624
150
100
50
ppm (t1)
20000
15000
10000
50000
0

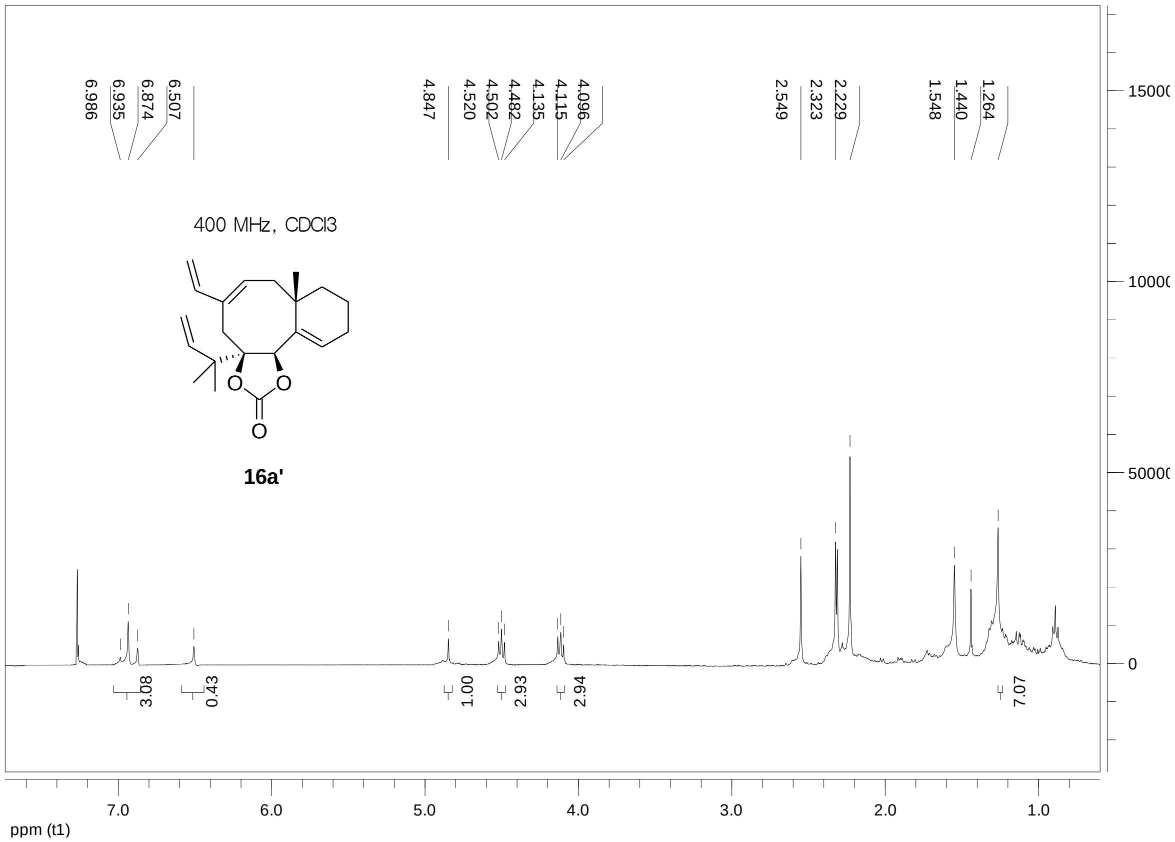
6.986
6.935
6.874
6.507
4.847
4.520
4.502
4.482
4.135
4.115
4.096
2.549
2.323
2.229
1.548
1.440
1.264
400 MHz, CDCl3
16a'
3.08
0.43
1.00
2.93
2.94
7.07
15000
10000
50000
0
7.0
6.0
5.0
4.0
3.0
2.0
1.0
ppm (t1)

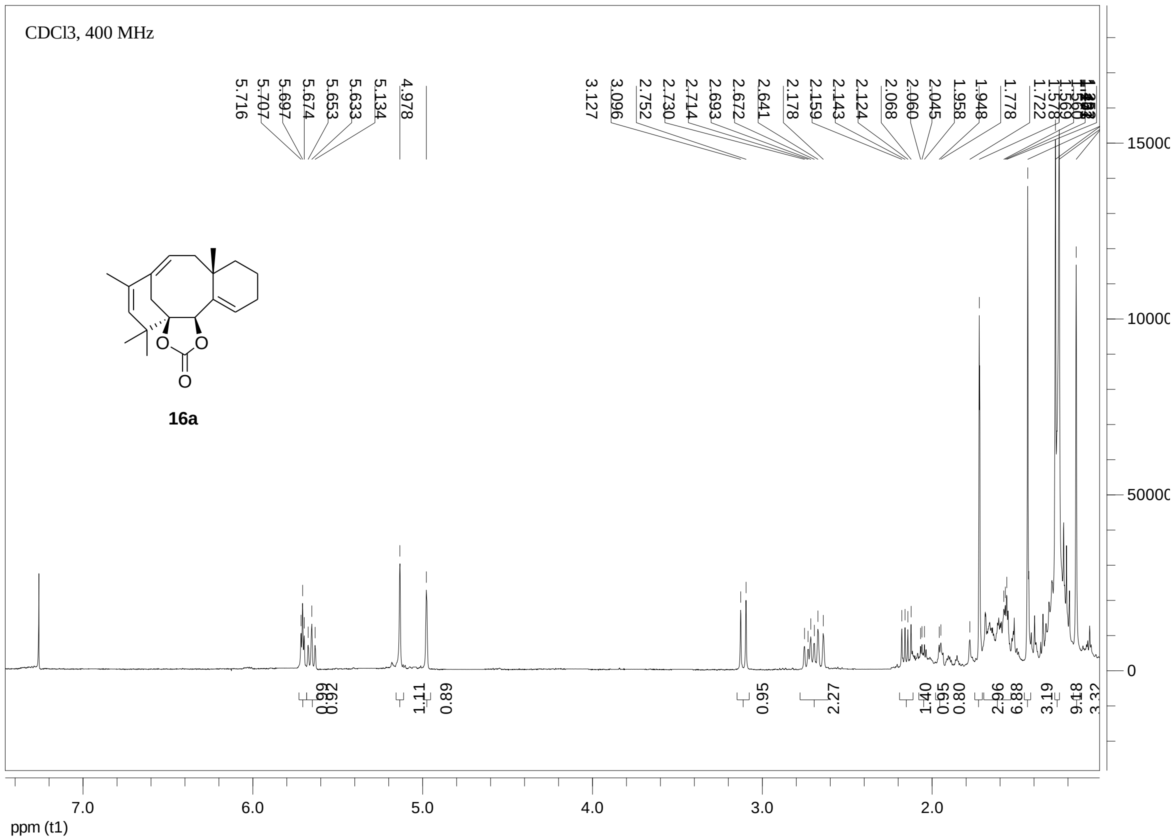
CDCl3, 400 MHz
16a
ppm (t1)

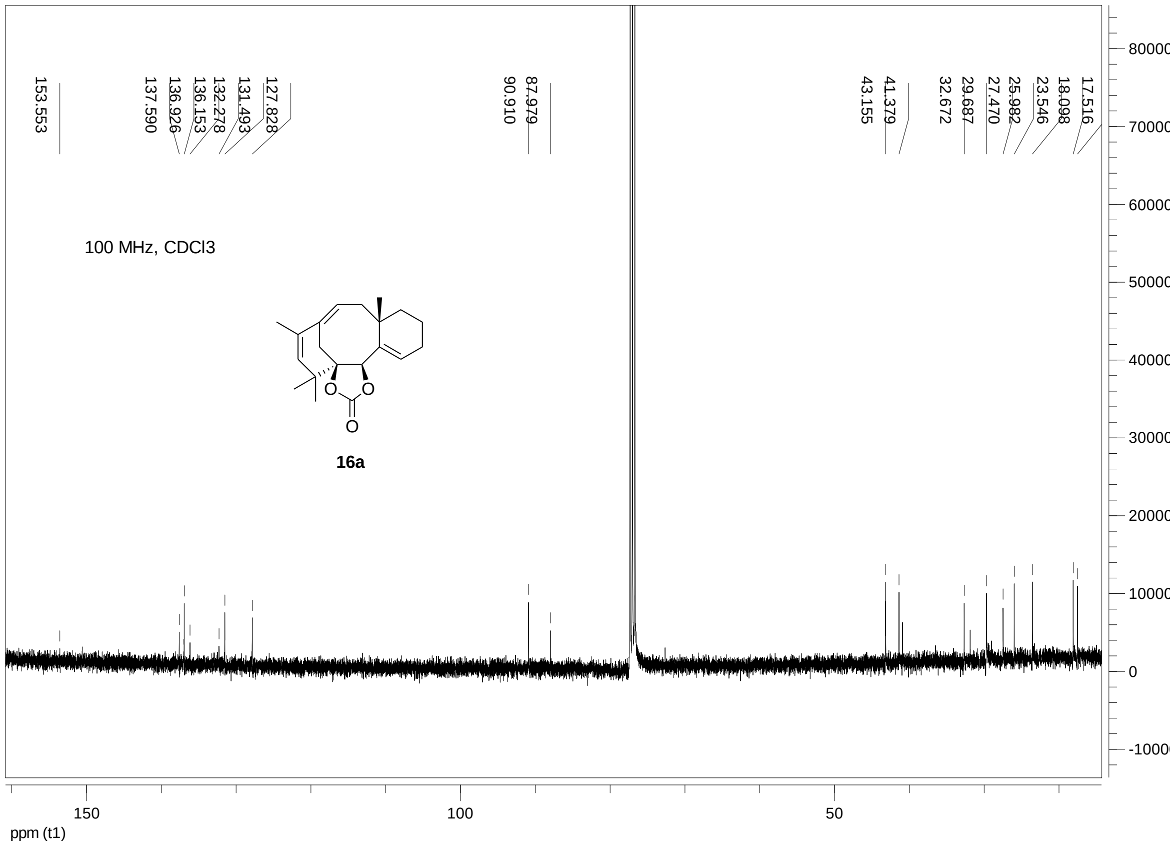
100 MHz, CDCl3
16a
ppm (t1)

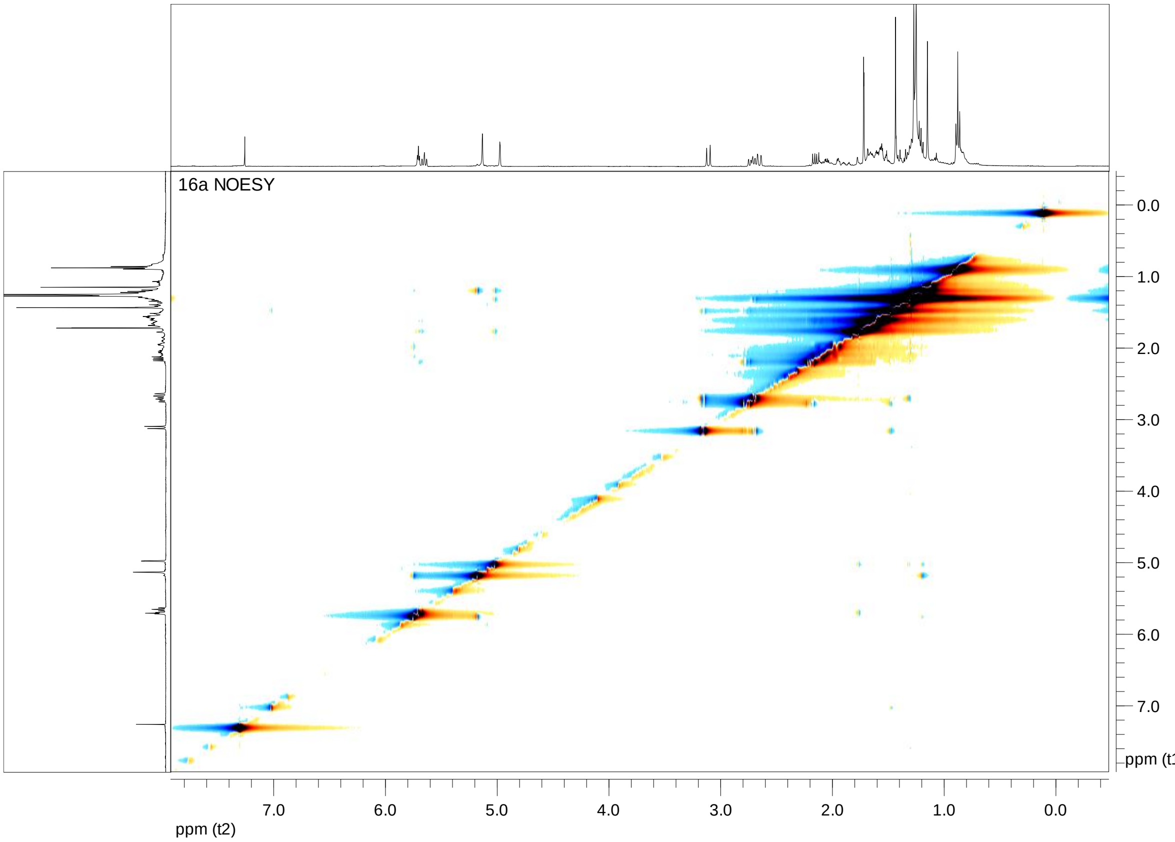
16a NOESY
ppm (t1
ppm (t2)

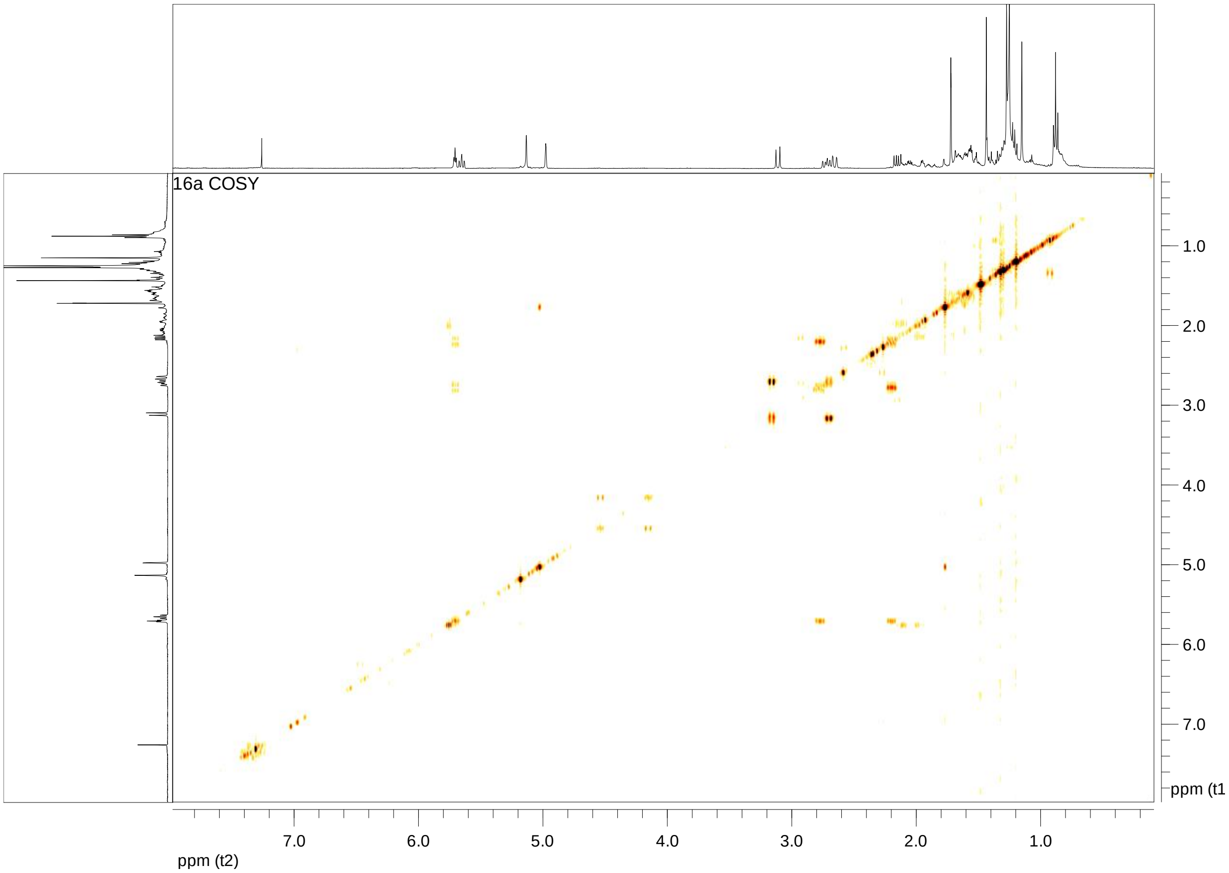
16a COSY
ppm (t1
ppm (t2)

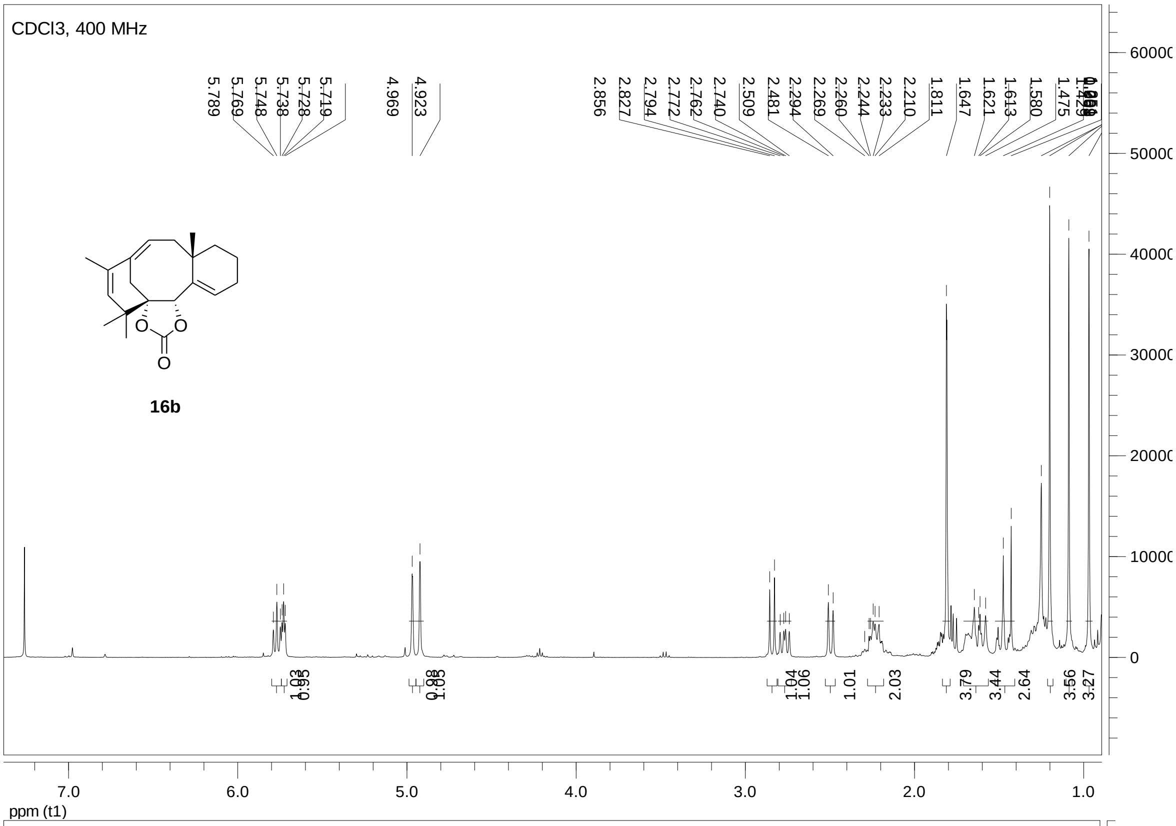

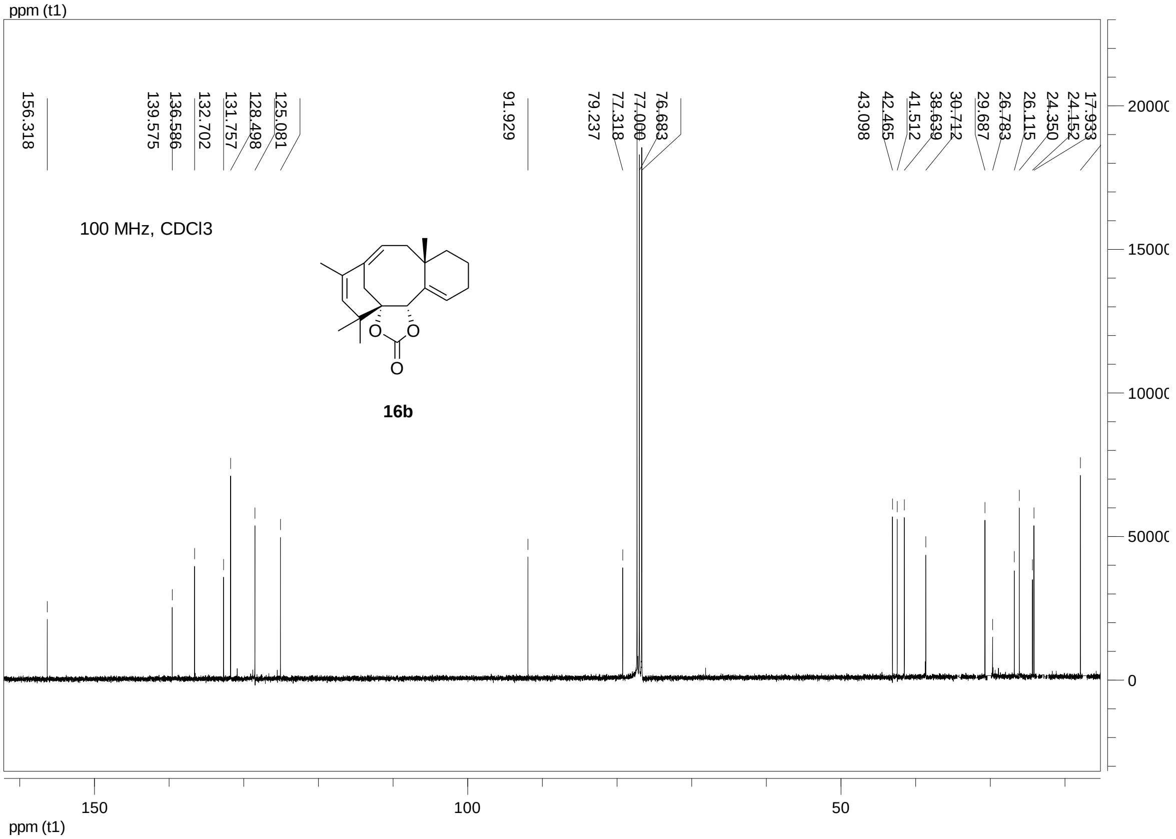

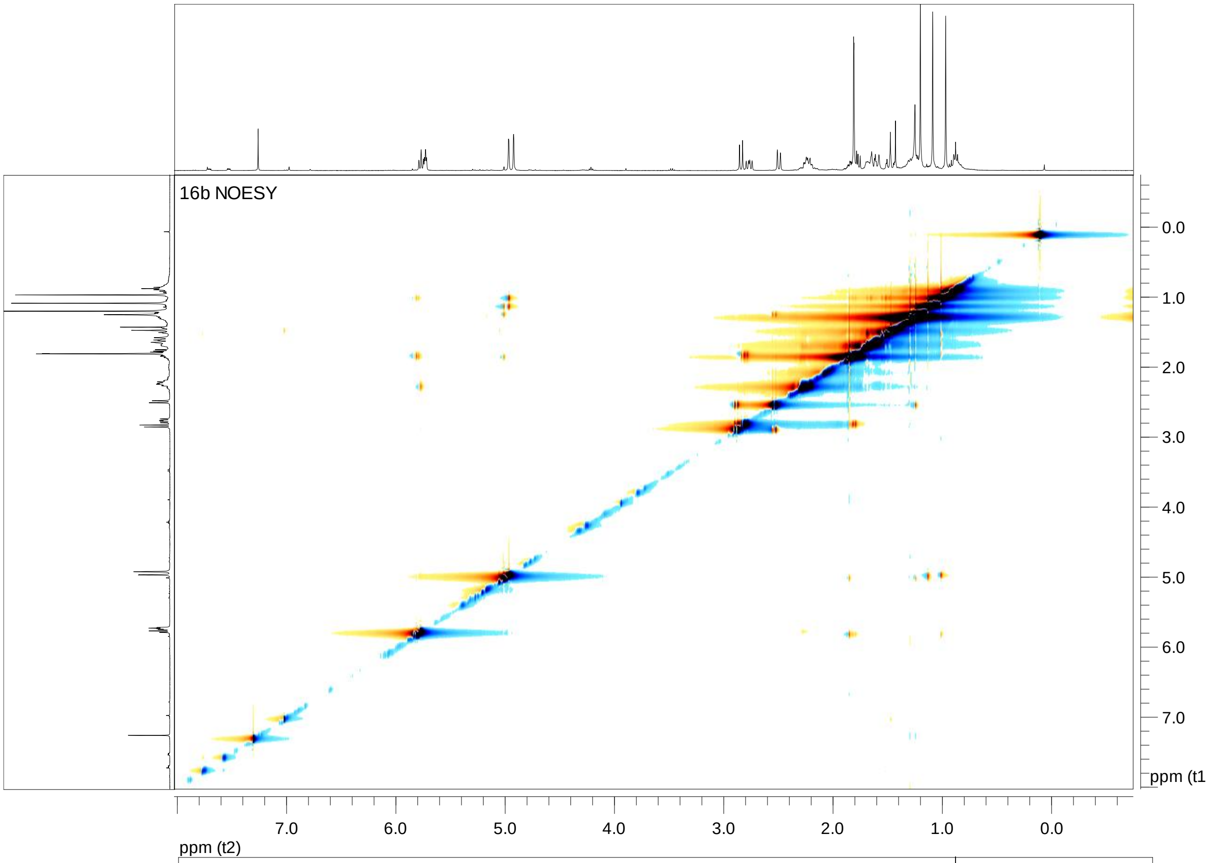
16b NOESY
ppm (t1
0.0
1.0
2.0
3.0
4.0
5.0
6.0
7.0
7.0
6.0
5.0
4.0
3.0
2.0
1.0
0.0
ppm (t2)

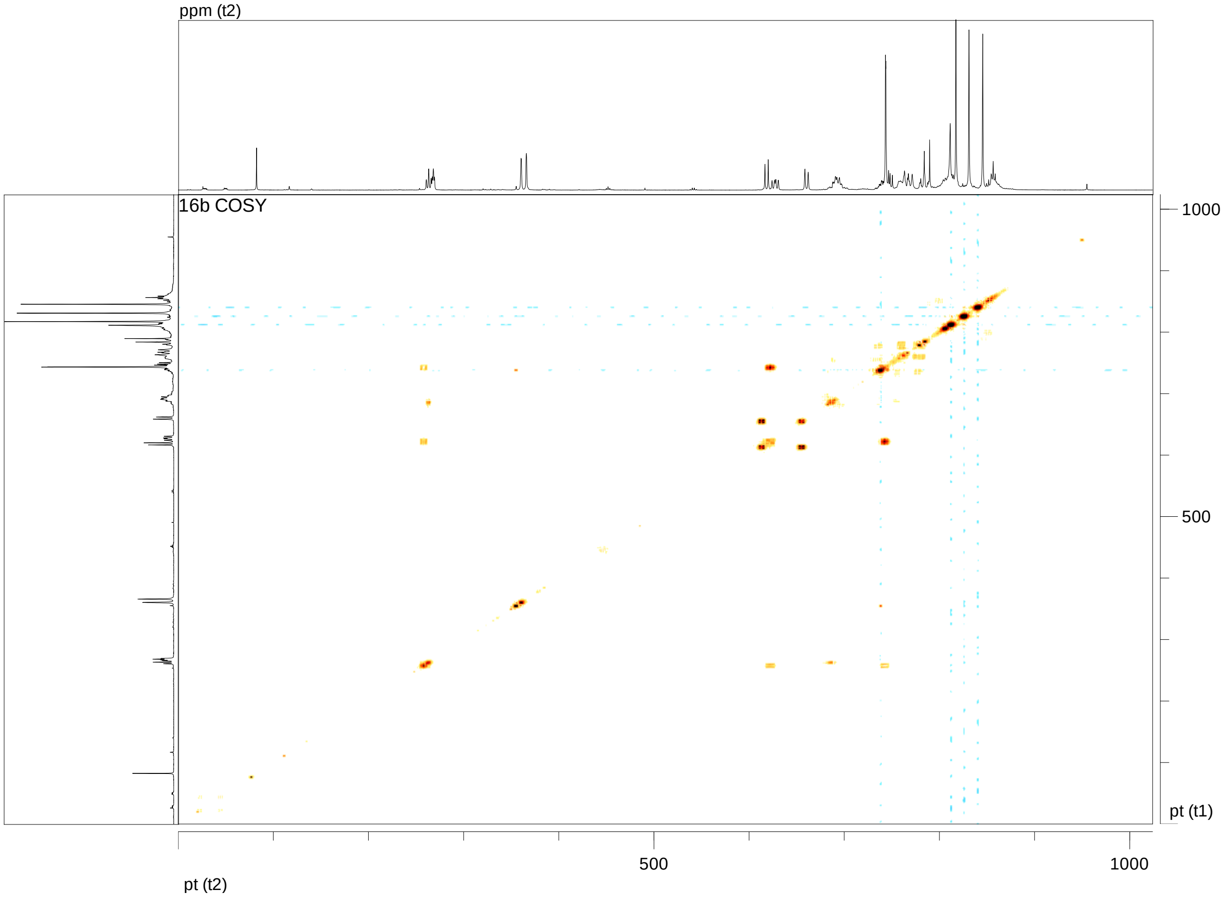
16b COSY
1000
500
pt (t1)
500
1000
pt (t2)

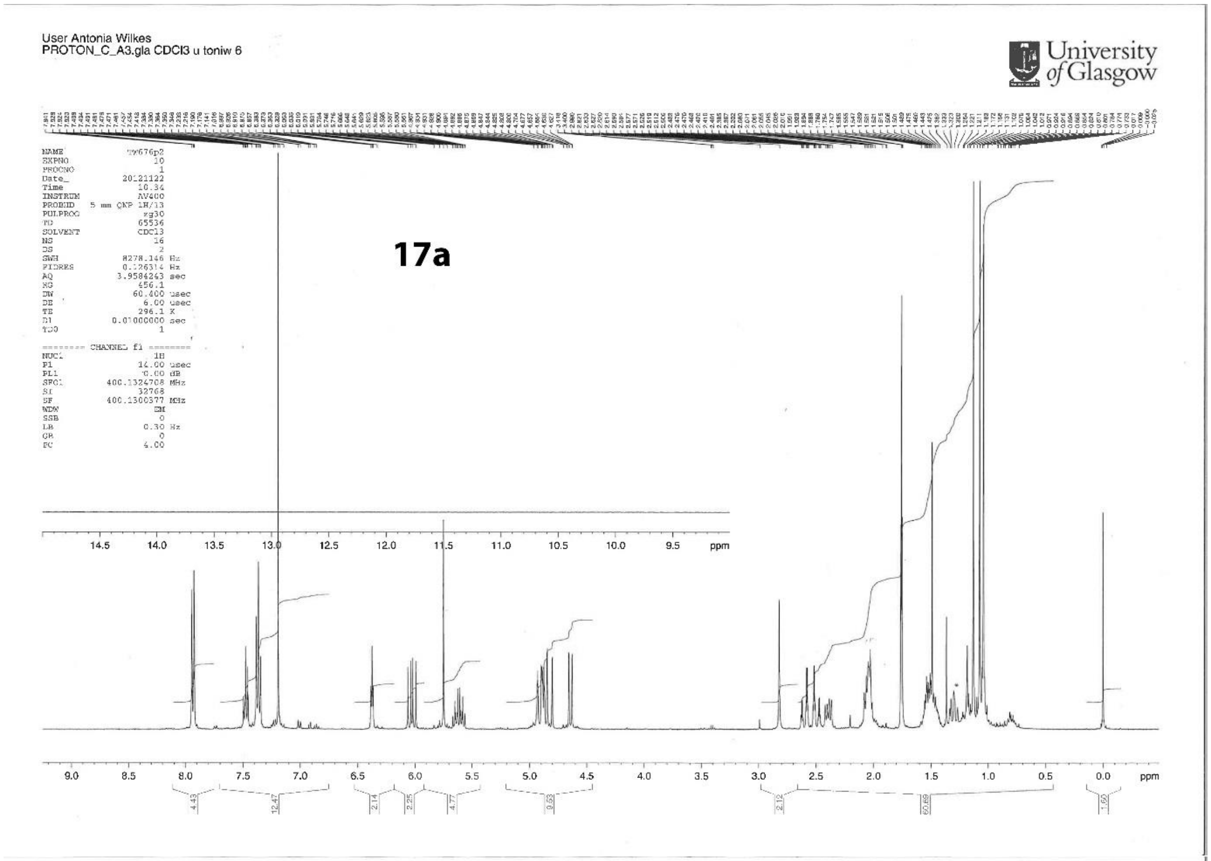
User Antonia Wilkes
PROTON_C_A3.gla CDCl3 u toniw 6
University of Glasgow
17a
NAME TW676p2
EXPNO 10
PROCNO 1
Date_ 20121122
Time 10.34
INSTRUM AV400
PROBHD 5 mm QNP 1H/13
PULPROG zg30
TD 65536
SOLVENT CDCl3
NS 16
DS 2
SWH 8278.146 Hz
FIDRES 0.126314 Hz
AQ 3.9584243 sec
RG 456.1
DW 60.400 usec
DE 6.00 usec
TE 296.1 K
D1 0.01000000 sec
TD0 1
======== CHANNEL f1 ========
NUC1 1H
P1 14.00 usec
PL1 0.00 dB
SFO1 400.1324708 MHz
SI 32768
SF 400.1300377 MHz
WDW EM
SSB 0
LB 0.30 Hz
GB 0
PC 4.00
ppm
4.43
12.47
2.14
2.35
4.77
9.53
2.12
60.69
1.50

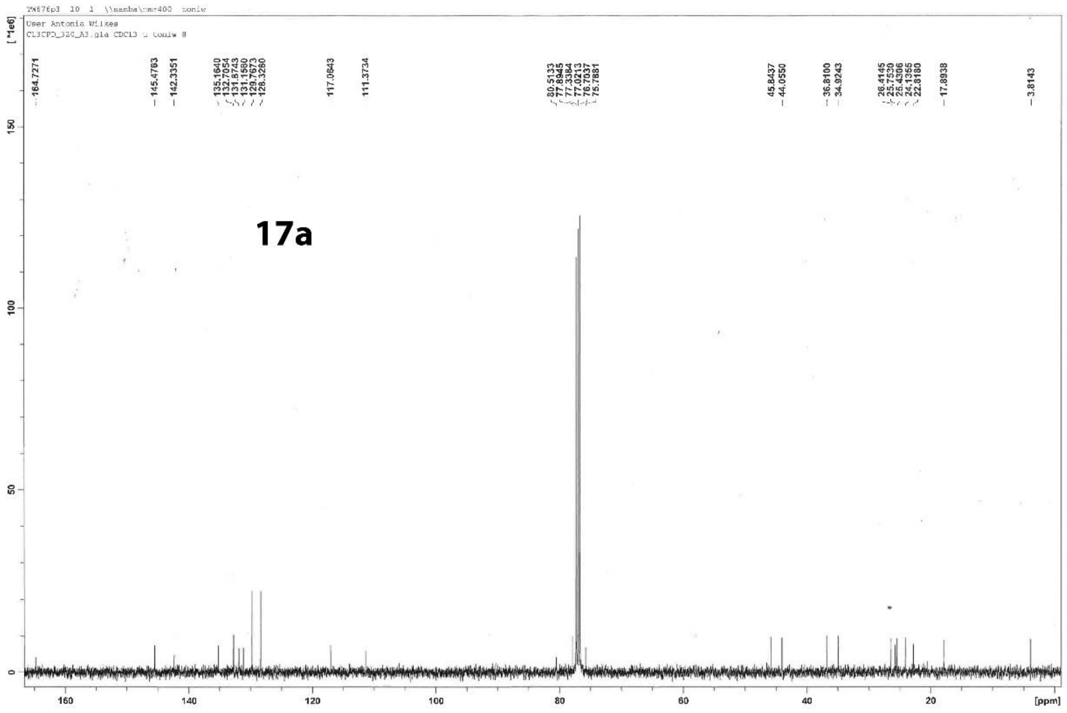
User Antonia Wilkes
17a
164.7271
145.4763
142.3351
135.1640
132.7054
131.8743
131.1560
129.7673
128.3260
117.0843
111.3734
80.5133
77.8945
77.3364
77.0213
76.7037
75.7881
45.8437
44.0550
36.8100
34.9243
26.4145
25.7530
25.4306
24.1355
22.3180
17.8938
3.8143
[ppm]

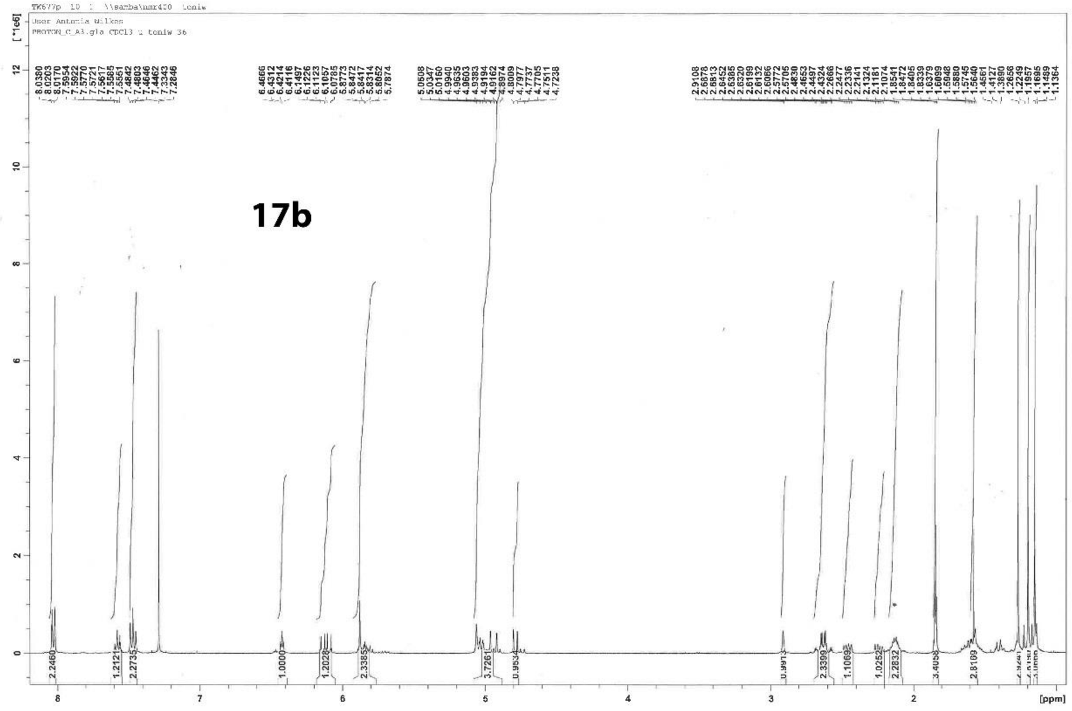
17b
[ppm]

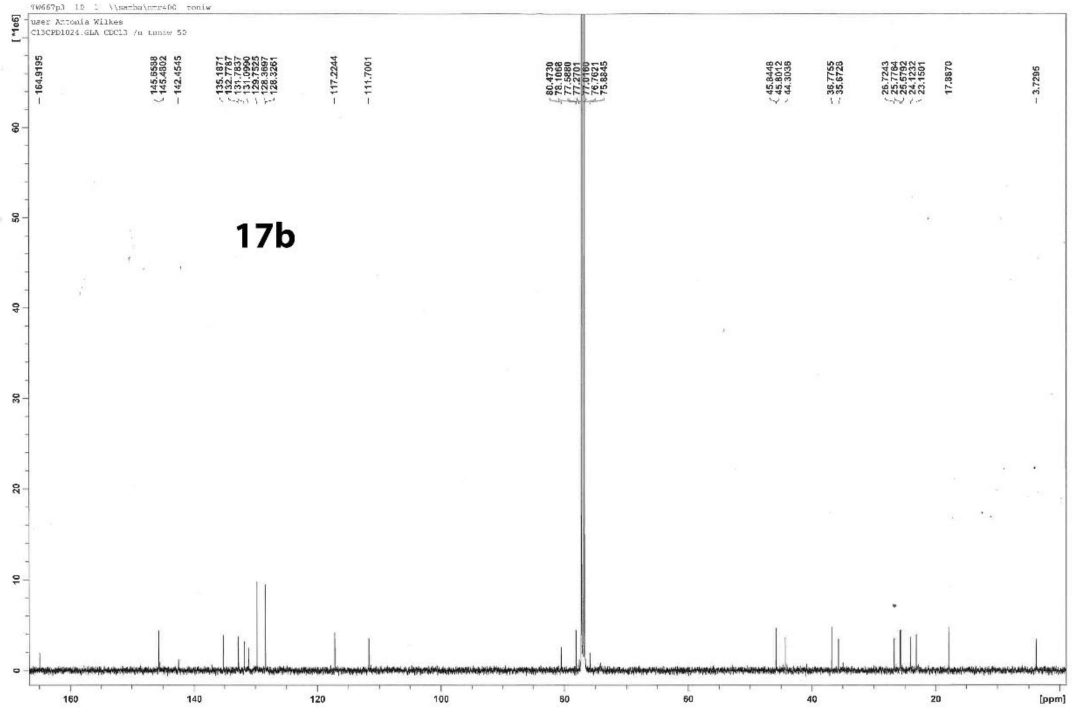
17b
[ppm]

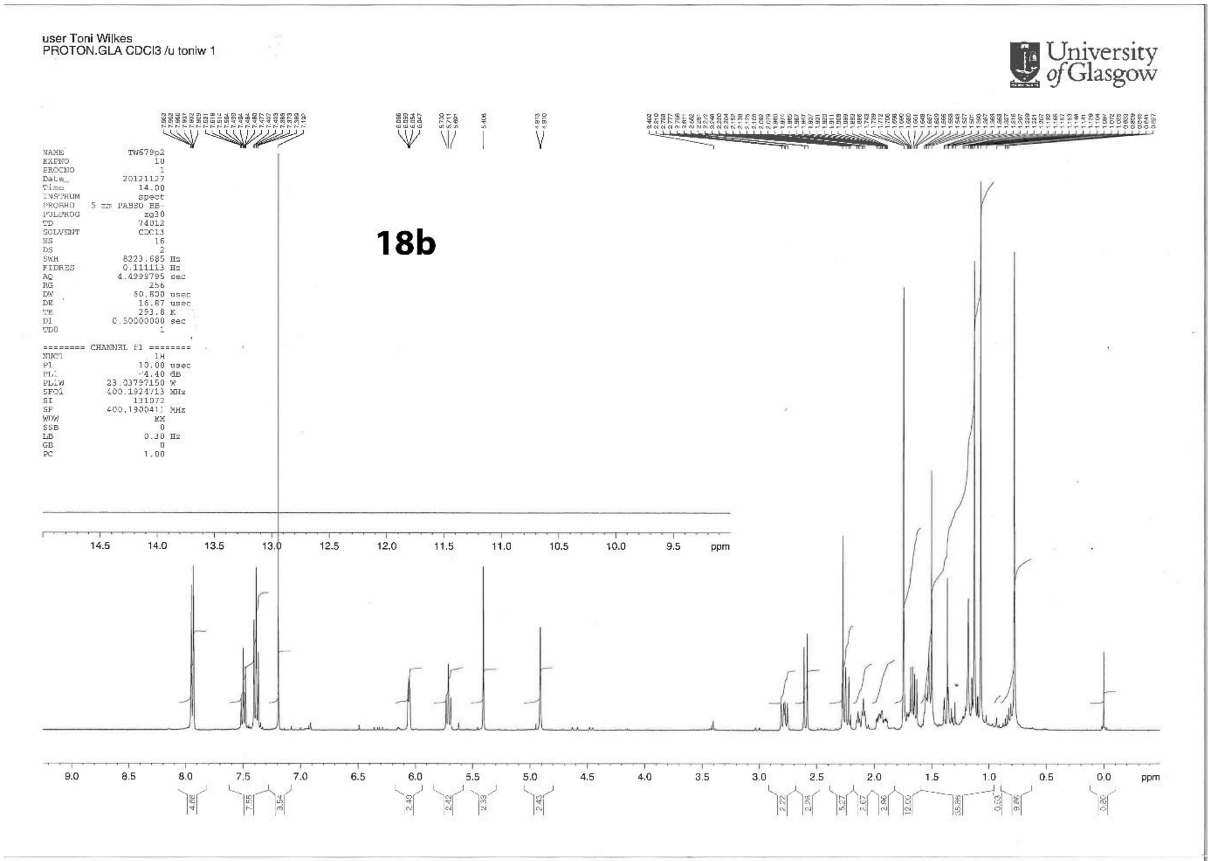
user Toni Wilkes
PROTON.GLA CDCl3 /u toniw 1
University of Glasgow
18b
ppm

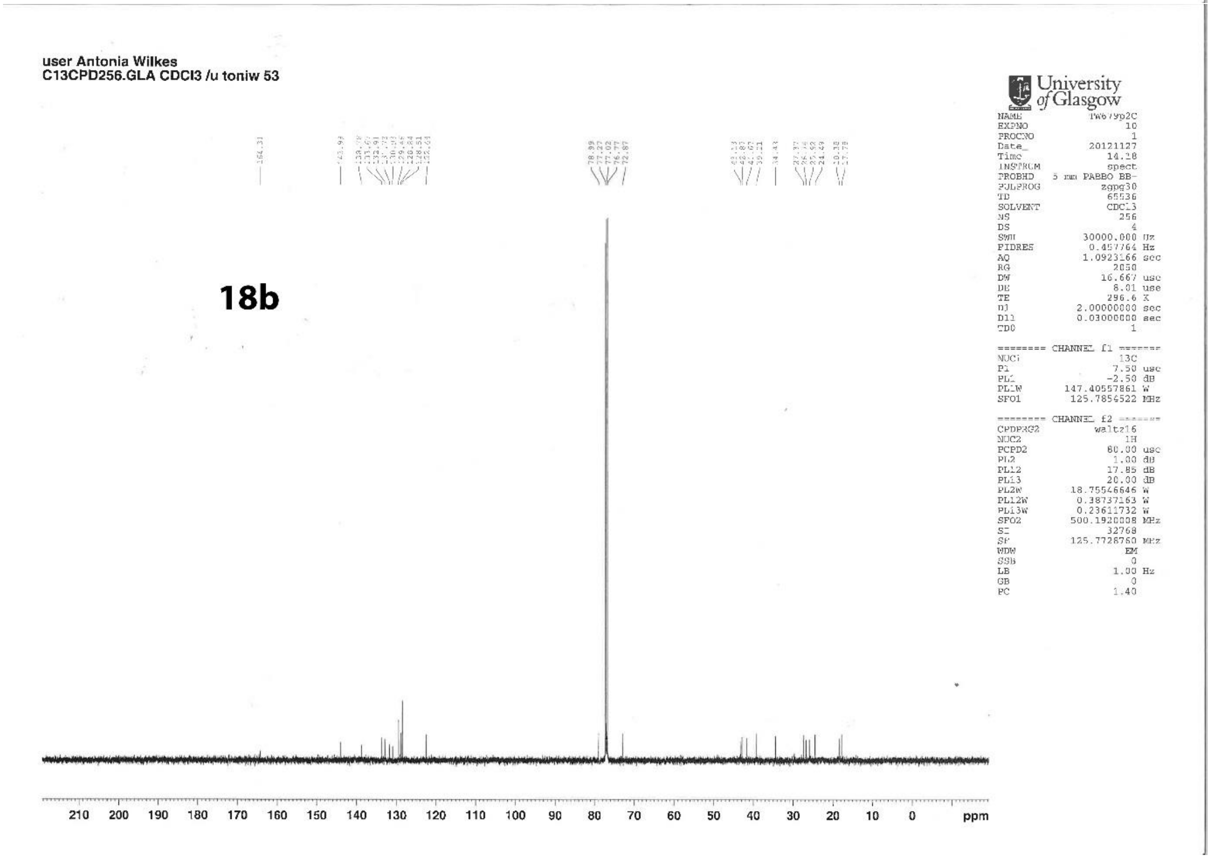
user Antonia Wilkes
C13CPD256.GLA CDCl3 /u toniw 53
University of Glasgow
18b
ppm

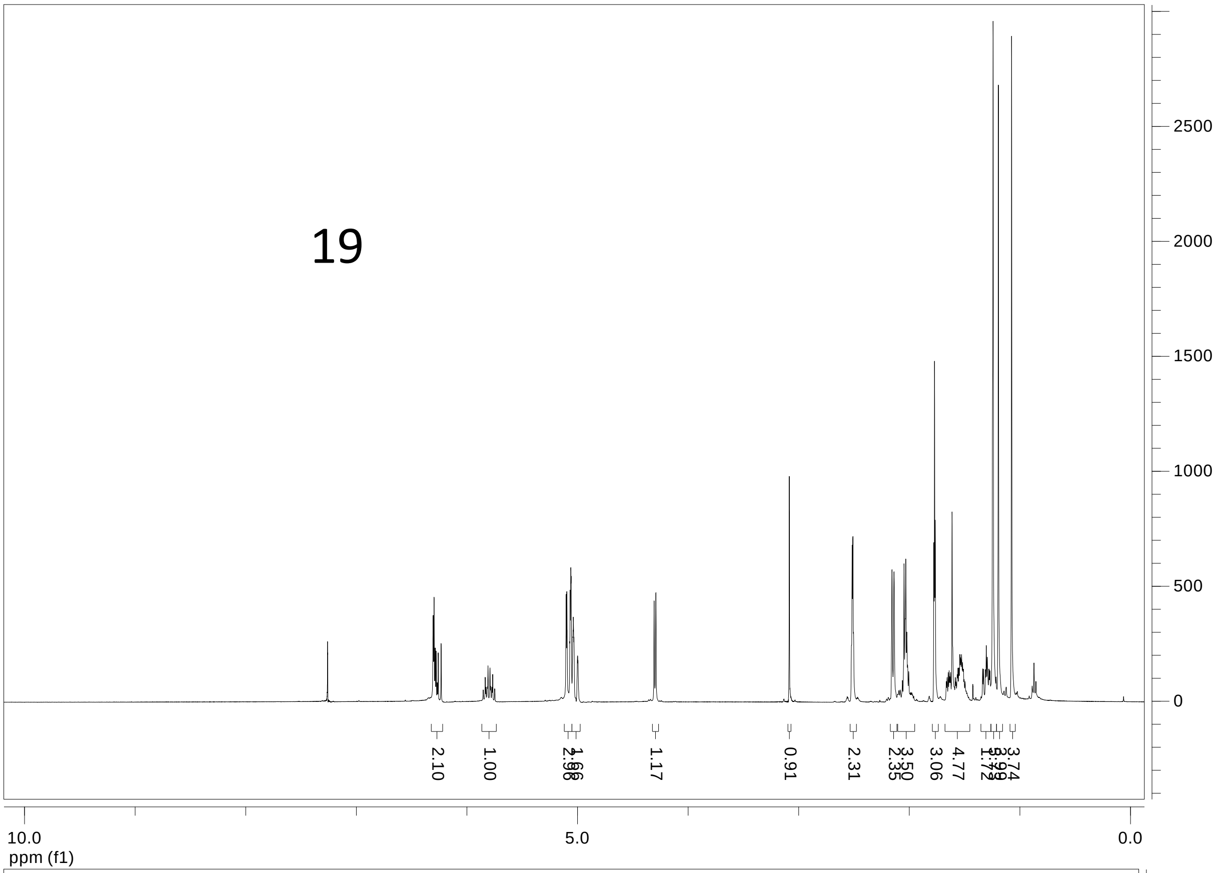
19
2.10
1.00
1.17
0.91
2.31
2.35
3.50
3.06
4.77
3.74
0
500
1000
1500
2000
2500
10.0
5.0
0.0
ppm (f1)

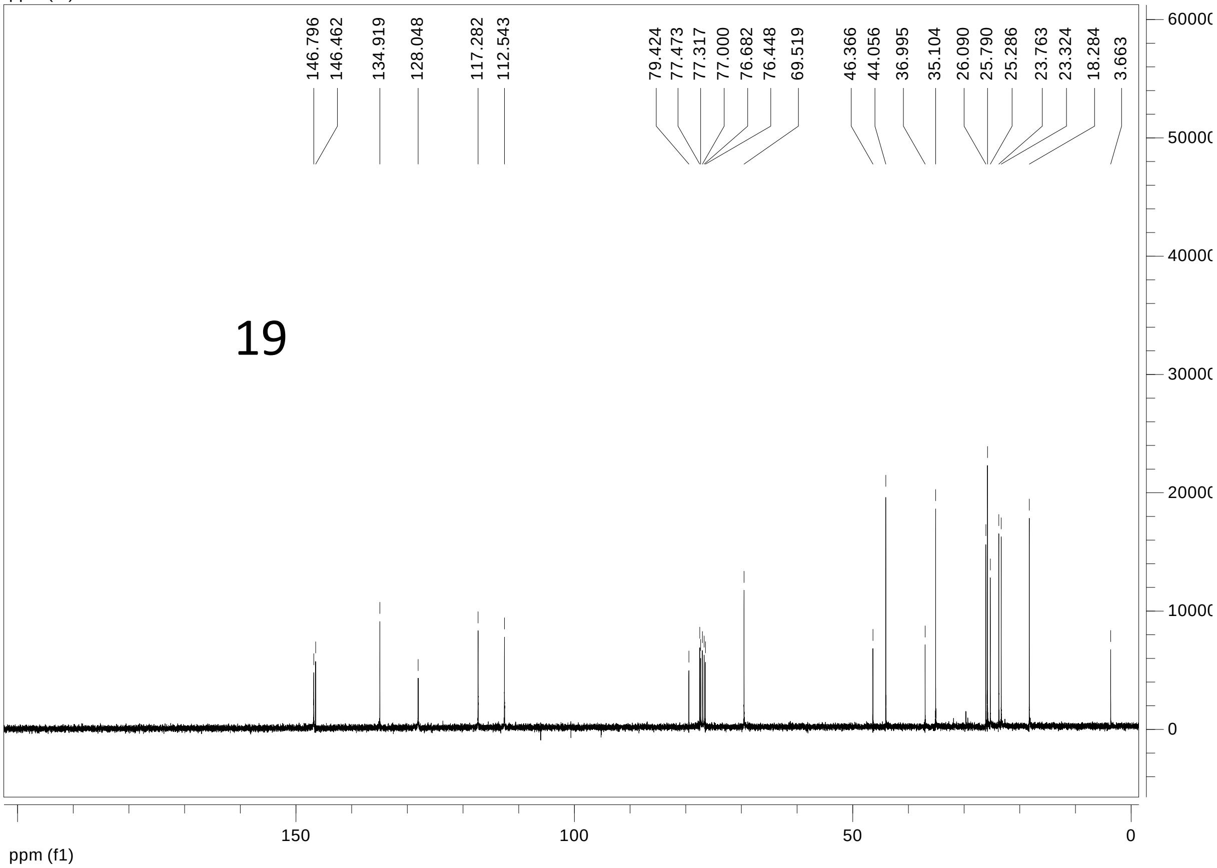
19
146.796
146.462
134.919
128.048
117.282
112.543
79.424
77.473
77.317
77.000
76.682
76.448
69.519
46.366
44.056
36.995
35.104
26.090
25.790
25.286
23.763
23.324
18.284
3.663
0
10000
20000
30000
40000
50000
60000
150
100
50
0
ppm (f1)

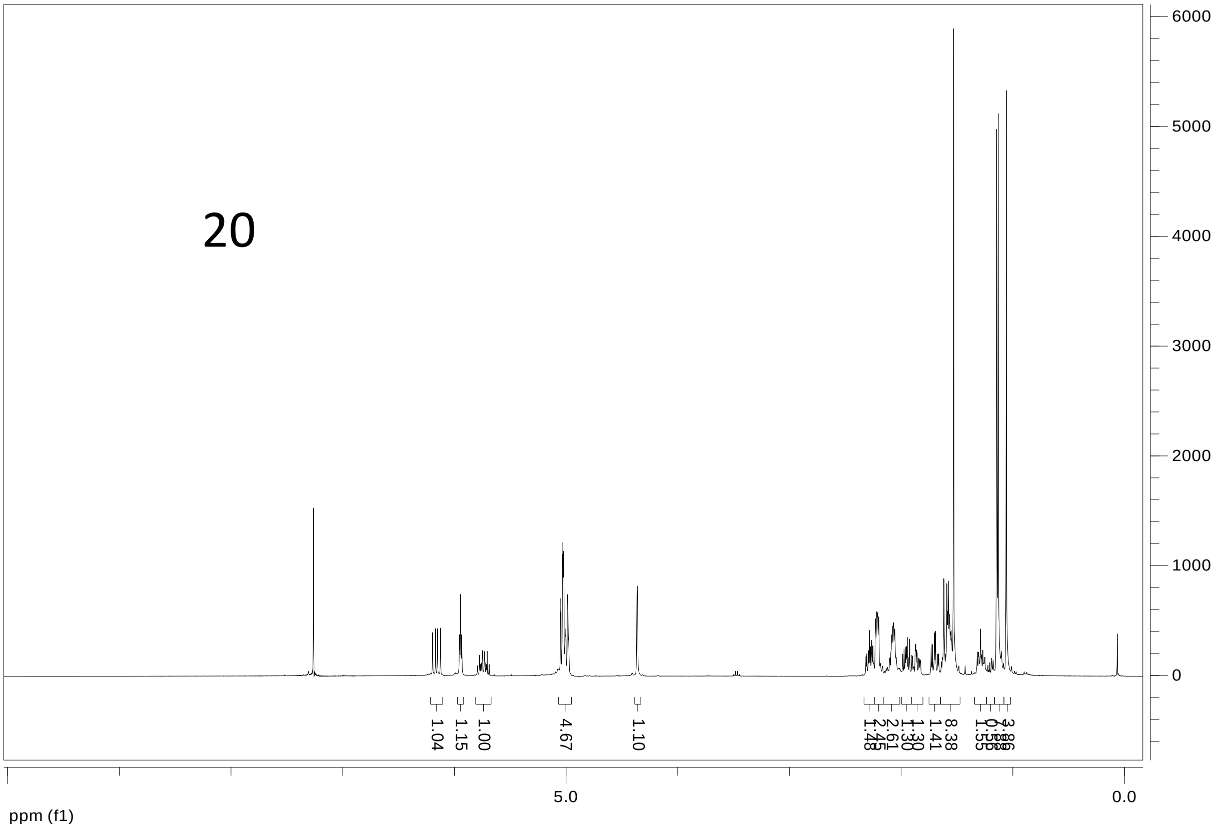
20
1.04
1.15
1.00
4.67
1.10
1.48
1.41
8.38
1.55
5.0
0.0
ppm (f1)
6000
5000
4000
3000
2000
1000
0

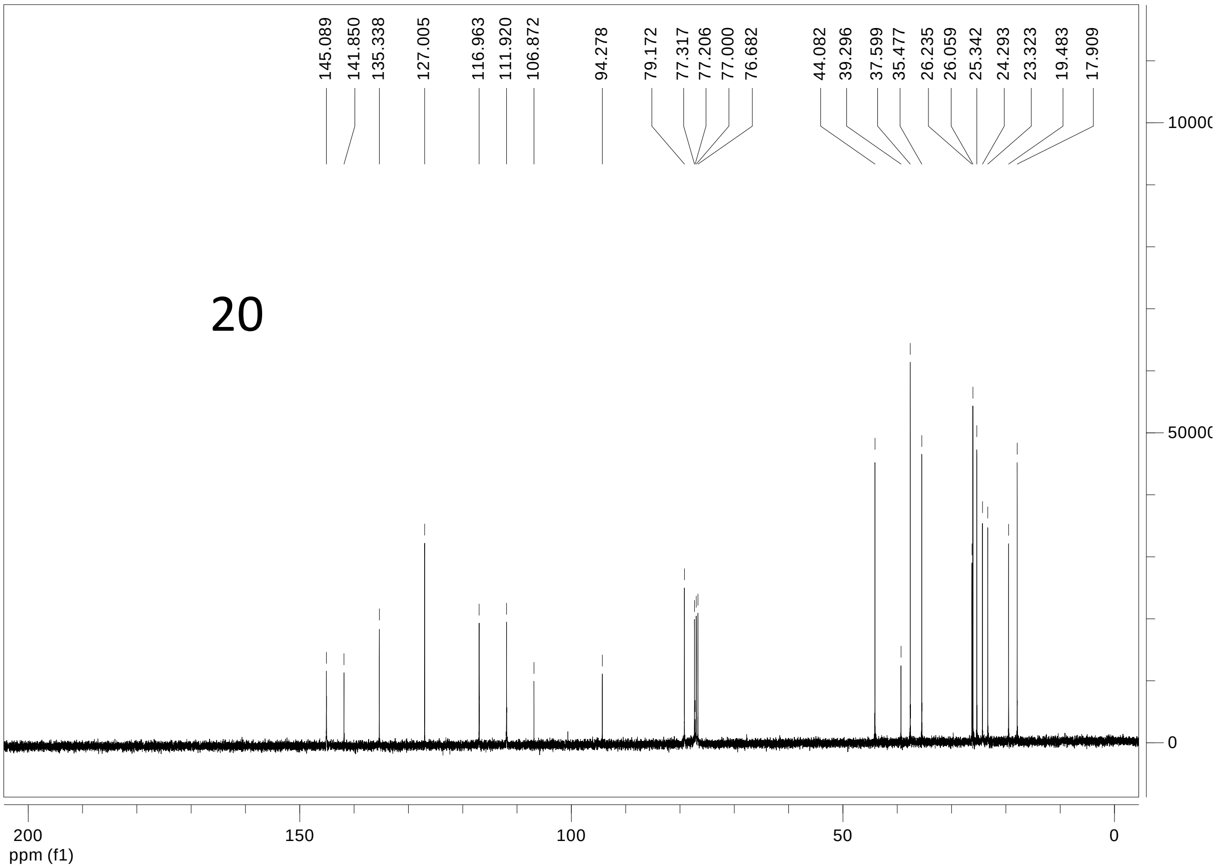
145.089
141.850
135.338
127.005
116.963
111.920
106.872
94.278
79.172
77.317
77.206
77.000
76.682
44.082
39.296
37.599
35.477
26.235
26.059
25.342
24.293
23.323
19.483
17.909
20
200
150
100
50
0
ppm (f1)
0

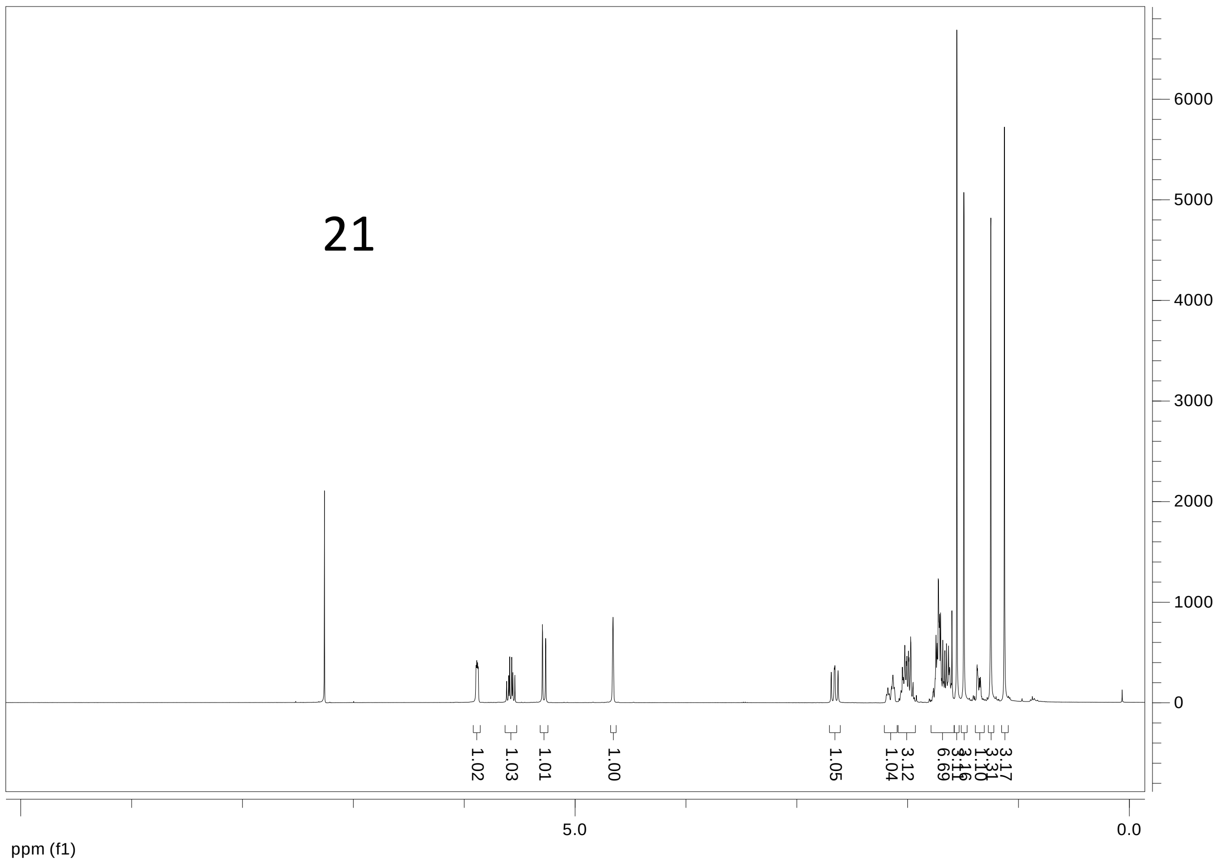
21
1.02
1.03
1.01
1.00
1.05
1.04
3.12
6.69
5.0
0.0
ppm (f1)

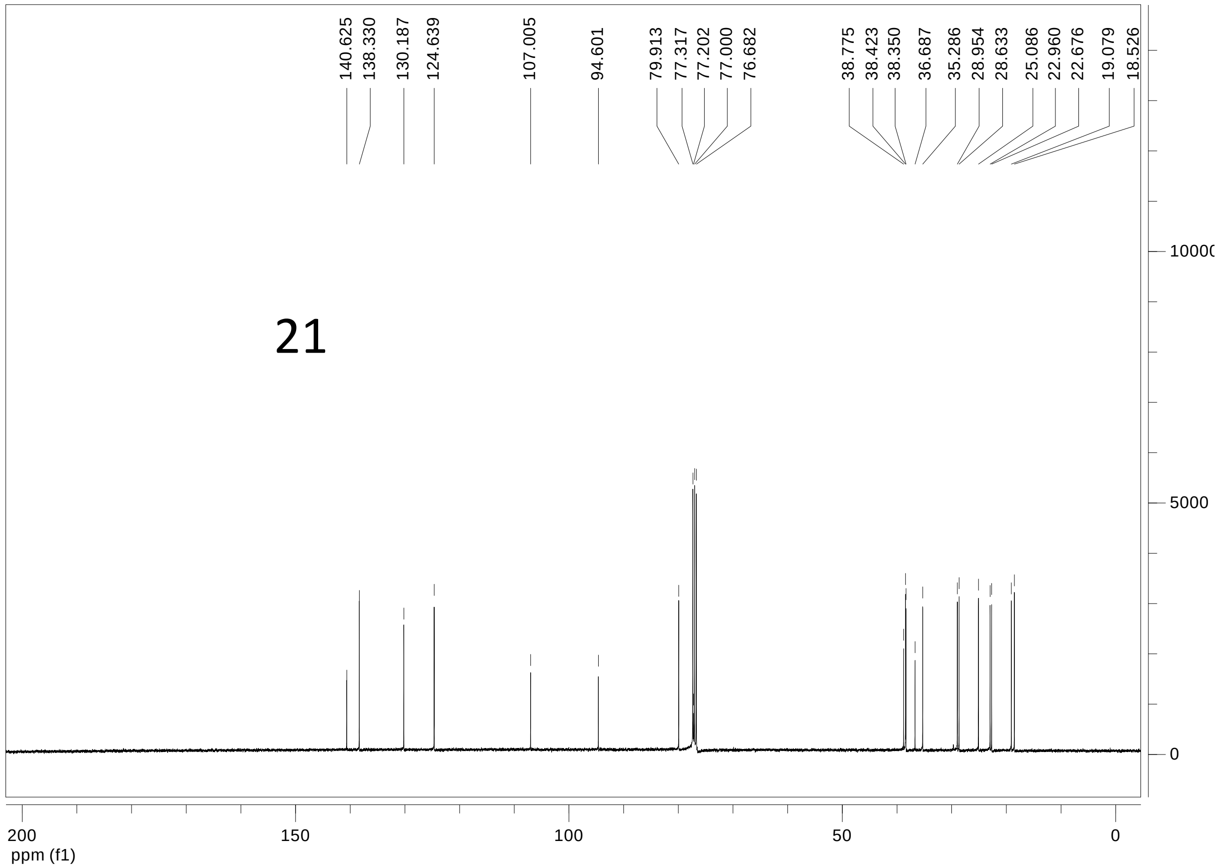
140.625
138.330
130.187
124.639
107.005
94.601
79.913
77.317
77.202
77.000
76.682
38.775
38.423
38.350
36.687
35.286
28.954
28.633
25.086
22.960
22.676
19.079
18.526
21
200
150
100
50
0
ppm (f1)

39

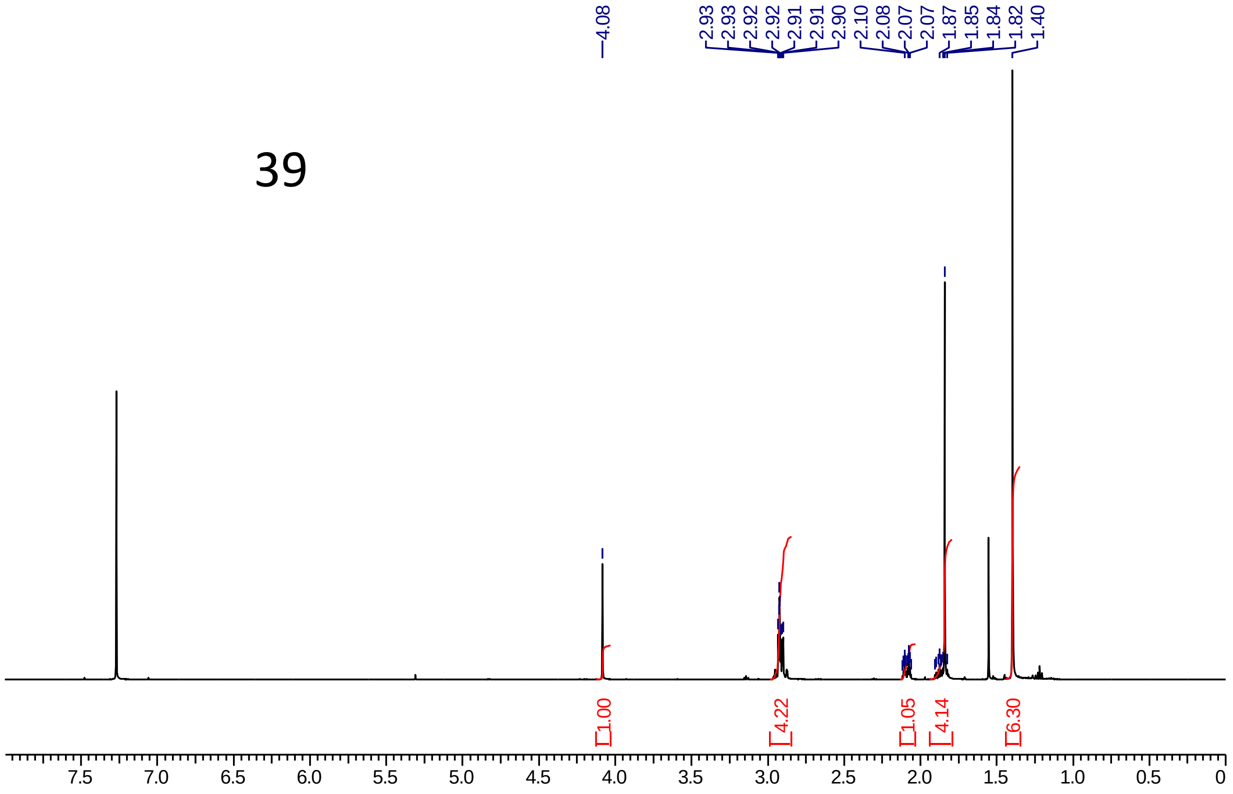

4.08
2.93 2.93 2.92 2.92 2.91 2.91 2.90
2.10 2.08 2.07 2.07
1.87 1.85 1.84 1.82
1.40
1.00
4.22
1.05
4.14
6.30
7.5 7.0 6.5 6.0 5.5 5.0 4.5 4.0 3.5 3.0 2.5 2.0 1.5 1.0 0.5 0

39

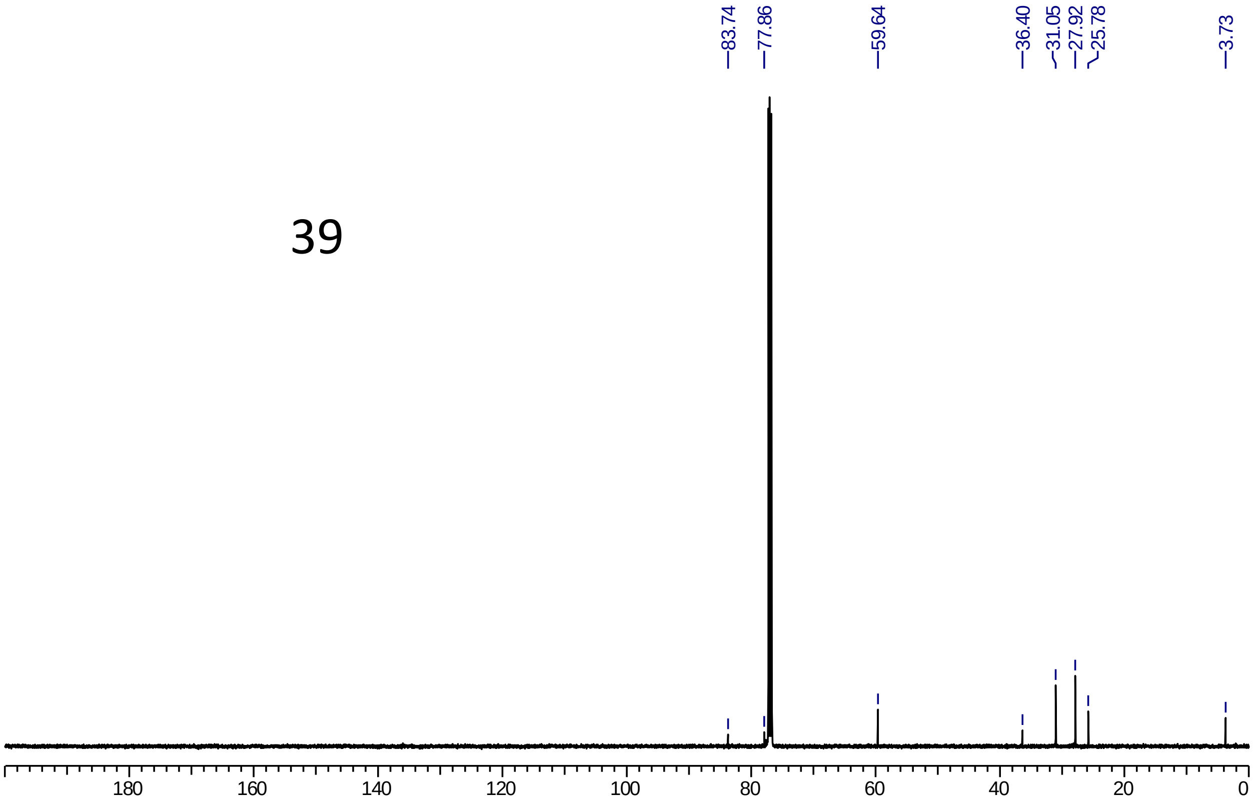

83.74
77.86
59.64
36.40
31.05
27.92
25.78
3.73
180 160 140 120 100 80 60 40 20 0

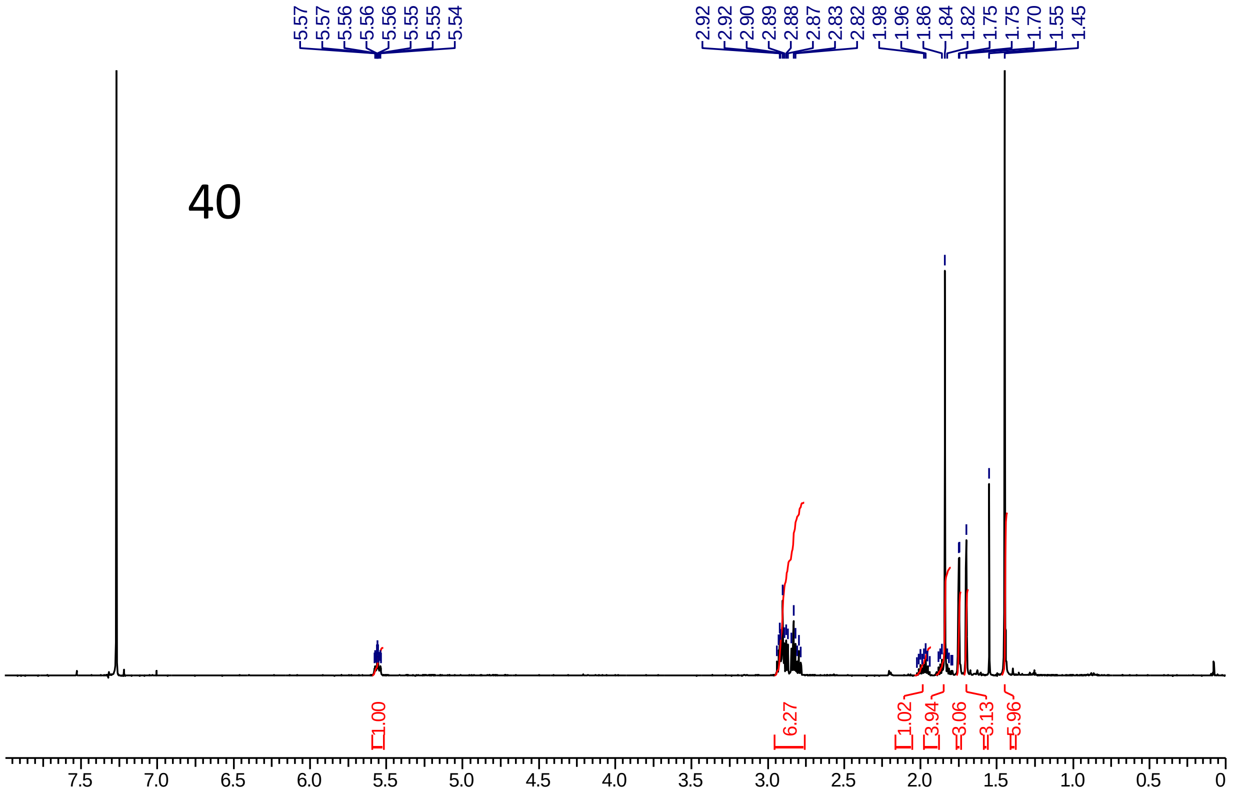
40
5.57
5.57
5.56
5.56
5.56
5.55
5.55
5.54
2.92
2.92
2.90
2.89
2.88
2.87
2.83
2.82
1.98
1.96
1.86
1.84
1.82
1.75
1.75
1.70
1.55
1.45
1.00
6.27
1.02
3.94
3.06
3.13
5.96

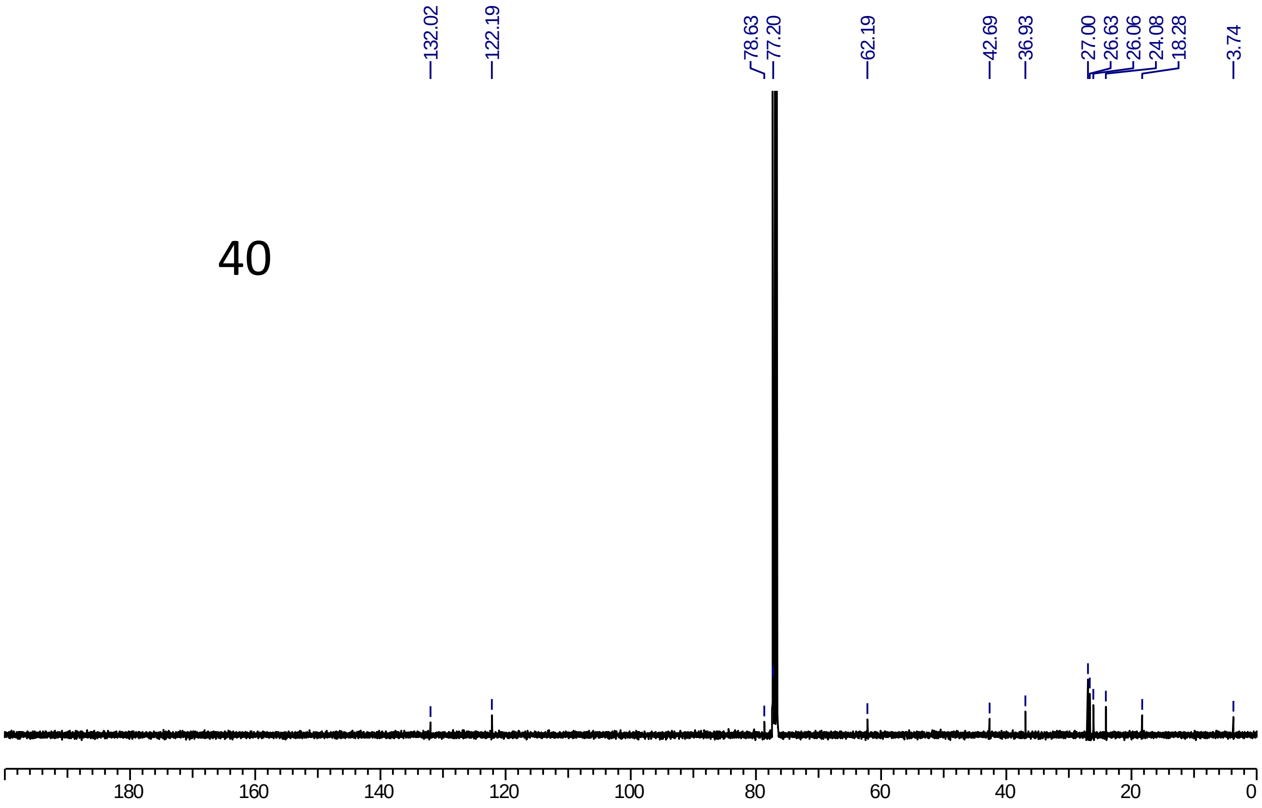
40
132.02
122.19
78.63
77.20
62.19
42.69
36.93
27.00
26.63
26.06
24.08
18.28
3.74

The NMR spectra of compounds **24**, **26-31**, **32a,b-35a,b**, **36**, **41**, **42a,b-43a,b**, **44** and **45** are reported in the Supporting Information of the preliminary account of this work:
A. Letort, R. Aouzal, C. Ma, D.-L. Long, J. Prunet, *Org. Lett*. **2014**, *16*, 3300-3303.
